# Tuning the Photophysical Properties of Acceptor–Donor–Acceptor Di-2-(2-oxindolin-3-ylidene) Malononitrile Materials via Extended π–Conjugation: A Joint Experimental and Theoretical Study

**DOI:** 10.3390/ma16196410

**Published:** 2023-09-26

**Authors:** Shiwei Ren, Amirhossein Habibi, Pingping Ni, Yuexing Zhang, Abderrahim Yassar

**Affiliations:** 1 Zhuhai Fudan Innovation Institute of Fudan University, Guangdong-Macao in-Depth Cooperation Zone in Hengqin, Hengqin 518057, China; shiwei_ren@fudan.edu.cn; 2 Laboratory of Physics of Interfaces and Thin Films, CNRS, Ecole Polytechnique, Institut Polytechnique de Paris, Route de Saclay, 91128 Palaiseau, France; 3 Shandong Provincial Key Laboratory of Monocrystalline Silicon Semiconductor Materials and Technology, Shandong Universities Engineering Research Center of Integrated Circuits Functional Materials and Expanded Applications, College of Chemistry and Chemical Engineering, Dezhou University, Dezhou 253023, China

**Keywords:** acceptor–donor–acceptor (A–D–A) materials, π–conjugation, electron mobility, perovskite solar cells

## Abstract

Many optoelectronic applications require organic semiconductor (OSC) materials with high electron affinity. In this work, a series of novel acceptor–donor–acceptor (A–D–A) materials with low-lying LUMO energy levels were designed and characterized. In this strategy, two acceptor dyes, bis-isatin and di-2-(2-oxindolin-3-ylidene) malononitrile, were connected by various π–bridges (benzene ring, benzo[c][1,2,5]thiadiazole, monothiophene, trithiophene). We varied the length of the π–conjugation of the central core and the linkage position of the acceptor core (4- vs. 6-position of the phenyl ring) to investigate the effect on the optical and electrochemical properties of the materials. We performed density functional theory (DFT) and time-dependent DFT (TD–DFT) studies to gain insight into the dyes’ electronic properties by determining the energy levels. Our findings demonstrate that with increasing acceptor strength and π–conjugation length of the core, the wavelength of the longest absorption maximum as well as their respective extinction coefficients are enhanced, which results in band-gap reduction either by lowering the LUMO and/or raising the HOMO energy level of the molecules. The potential practical utility of these materials as electron-transport materials for perovskite solar cells (PSCs) has been demonstrated.

## 1. Introduction

**Figure 1:**
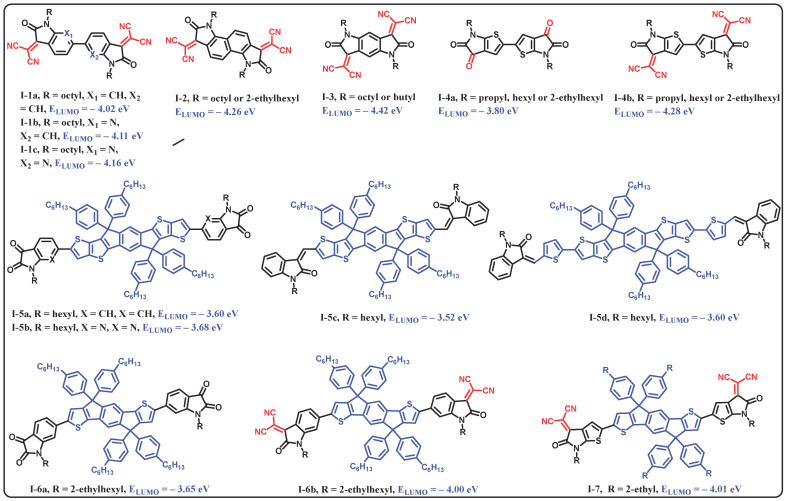
Chemical structures of bis-lactam-based organic materials reported in the literature.

OSC materials with low-lying LUMO energy levels are particularly interesting for many optoelectronic applications, in particular for n-channel organic field-effect transistors (OFETs), as non-fullerene acceptors and electron-transporting materials for perovskite solar cells [1,2,3]. To date, tremendous efforts have been devoted to exploring new building blocks for the fabrication of novel n-type OSCs. One key requirement for developing n-type OSC is the high electron affinity, which facilitates electron injection and stabilizes injected electrons. This is usually expressed in terms of the energy level of the LUMO, which should be sufficiently low-lying (≤−3.5 eV) to facilitate electron injection from the cathode [4]. Modifications of the chemical structure and the nature of the electron-withdrawing building block are mainly used to determine the LUMO energy level. Various building blocks have been employed to construct new n-type OSCs. Among them, bis-lactams are of particular interest owing to their strong electron-withdrawing ability because of their carbonyl groups, which facilitate strong intermolecular interactions [5,6,7]. Based on bis-lactam design strategies, various novel electron-deficient building blocks for high-performance OSCs have been reported [8,9]. Isatin and its dicyanovinyl derivatives belong to this family, and they are good synthetic precursors of many natural products and promising n-type acceptor–donor–acceptor (A–D–A)–type OSCs due to their planar framework with two electron-withdrawing carbonyl groups [10,11,12]. In the past, various D–A-type dyad and triad architectures have been designed and synthesized, and their photophysical properties are largely influenced by how the dyad and triad systems are constructed (A–D, D–A–D, A–D–A, etc.). Through appropriate chemical modification of the isatin core, a significant number of n-type OSCs with a low LUMO energy level have been reported. Kuo and coworkers reported molecular design strategies for three di-2-(2-oxindolin-3-ylidene) malononitrile derivatives (I–1a, 1b, and 1c in Figure 1) with various numbers of nitrogen substituents on the phenyl rings for use as n-type OFET materials [13]. By selectively controlling the number of nitrogen atoms introduced in the phenyl group, they demonstrated that the LUMO energy level could be tuned from −4.02 eV for the unsubstituted derivative to −4.16 eV for the disubstituted derivative. The introduction of the amine functionality not only lowers the LUMO level but also enhances the molecule’s coplanarity, which results in a moderate electron mobility of 0.059 cm^2^ V^−1^ s^−1^. However, the replacement of a phenyl ring with a pyridine had a slight effect on the absorption spectra. The same group later reported the synthesis and characterization of two derivatives of fused angular-shaped naphthalene bis-isatin (I–2) cores [14]. These new derivatives showed a low-lying LUMO energy level of −4.25 eV, lower than that previously reported, making them suitable materials for air-stable n-type OSCs. These naphthalene derivatives, I–2, displayed a broad absorption with two main absorption bands at 300–450 and 550–800 nm, assigned as the π–π* and intramolecular charge transfer (ICT) transitions, respectively. Benzodipyrrole-2,6-dione-3,7-diylidenedimalononitrile, I–3, which was a structural analog to I–2 by replacing the naphthalene core with a phenyl moiety, was recently reported, and its electrochemical behavior was investigated by cyclic voltammetry (CV) [15]. The CV results showed reversible reduction−oxidation processes, and the LUMO levels were estimated to be −4.42 eV. To analyze the effect of acceptor strength on the optoelectronic properties, Mori et al. synthesized I–4a in which the phenyl ring of the isatin core is replaced by a thiophene ring [16]. I–4b exhibited considerably stronger electron deficiency properties than the I–4a parent, with a deep LUMO level of −4.28 eV. Compared with I–4a, the absorption spectra of the thienoisatin derivatives displayed broader and redshifted features with a λ_max_ of 650 nm. The redshift was due to an increased D–A character of I-4b compared to I-4a. All these features make these materials promising for use as n-type OFET materials, with an electron mobility of 0.10 cm^2^ V^−1^ s^−1^. Zhang and coworkers reported two series of A–D–A–type OSC-based isatin derivatives with a rigid indacenodithieno[3,2-b]thiophene moiety acting as a central bridge and both ends capped with strong electron-withdrawing indole (I–5a) or pyrrolo[4,5-b]pyridin-2,3-dione (I–5b) [17]. The replacement of the benzene ring with a pyridine moiety enhances the planarity and improves the structural ordering through noncovalent interactions due to the lower steric requirements of the nitrogen atom compared to the C-H unit, which results in a remarkable enhancement of hole mobility. In a separate study, the same authors showed that changing the end group from I–5c to the thienyl methylene-oxindole unit, I–5d, while keeping the same π–bridge, indacenodithieno[3,2-b]-thiophene, enables a high hole OFET mobility of over 0.27 cm^2^ V^−1^ s^−1^ [18]. A series of A–D–A–type OSCs based on indacenodithiophene as a bridging group were synthesized, and their opto-electrical properties were investigated by Zhao [19,20]. Compound I–6a in chloroform solution showed a major absorption peak at 540 nm, which underwent a considerable redshift for I–6b (675 nm) and I–7 (730 nm) because of the gradual increase in the electron-withdrawing ability of the acceptor moieties of these molecules. This redshift was accompanied by a reduction in the corresponding optical band-gap values from 1.95 eV for I–6a to 1.48 eV for I–6b and 1.43 eV for I–7. The LUMO energy level of these A–D–A–type OSC was found to vary significantly with the strength of the acceptor fraction. The LUMO energy of I–6b was significantly reduced to −4.02 eV. On the other hand, the LUMO energy of I–7 was close to that of I–6b.

In addition to their use as OFET materials, A–D–A–type OSCs with deep LUMO levels could be used as electron-transport materials for perovskite solar cells [21,22,23]. Recent works have shown that the energy level matching between the perovskite’s conduction band and the electron-transporting materials’ LUMO significantly impacts device performance by promoting electron extraction and inhibiting recombination between interfaces. When implemented in a perovskite solar cell, an acceptor material with a LUMO energy of approximately −3.80 eV can facilitate electron transfer from the bottom of the perovskite conduction band to its LUMO level.

Despite these studies, there has been no systematic study on the effect of substitution positions and conjugation lengths of the bridging groups on the photophysical and electrochemical properties of A–D–A–type OSC to understand the parameters required for ideal acceptor materials. We propose that modifying the π-linking units of the A–D–A OSC would tune the energy levels and photophysical properties of the resulting materials since the π-bridge in the A–D–A OSC plays an important role in regulating the absorption and charge-carrier mobility.

**Figure 2:**
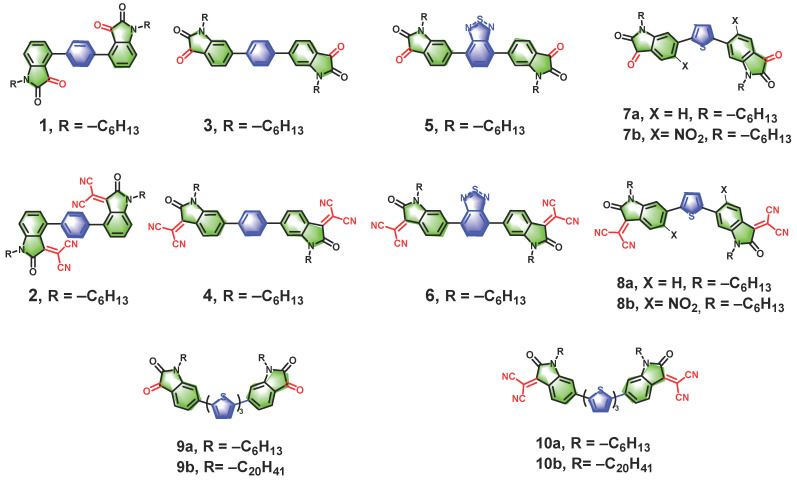
Chemical structures of bis-isatin and di-2-(2-oxindolin-3-ylidene) malononitrile derivatives.

Herein, we present the design and synthesis of a new set of small molecules with A–D–A–type OSC architectures that consist of π-conjugated bridges with different π-spacer lengths as donor units and two isatylidene malononitrile segments as the terminal acceptor groups (Figure 2). A-D-A-based OSCs comprise three structural features, including a donor π-conjugated system, solubilizing side chains, and two electron-withdrawing end groups that can be independently modified to tune the optical, electronic, and morphological properties. The choice of acceptor moieties and end-group plays an important role in modulating the LUMO level, absorption range, and molecular stacking. Most of the reported literature focused on indanone and rhodanine derivatives, herein we investigate a similar molecular design approach with isatin. The isatin moiety possesses four positions available for introducing functional groups. The 4 and 6 positions result in a linearly conjugated chromophore, while the substitutions at the 5 and 7 positions generate nonconjugated materials [24]. The objective is to vary the conjugation length by different π-spacer groups, to alter the linkage position of the phenyl of the isatin core (4-position vs. 6-position), and to analyze how these modifications affect the photophysical and electrochemical properties of these new dyes. DFT and TD–DFT studies were performed to gain insight into their electronic properties by determining the energy levels and maps of the HOMO and LUMO as well as the HOMO–LUMO gap. To demonstrate their ability to act as n-type OSCs, we performed SCLC measurements to probe their electron mobility. Electron-only devices were fabricated by spin-coating, and the trap-limited electron mobility was calculated using Mott-Gurney law. 10a exhibited an intrinsic moderate electron mobility of 8.94 × 10^−4^ cm^2^ V^−1^ s^−1^. The potential practical utility of these materials is demonstrated as electron-transport materials (ETMs) for PSCs, with maximum power conversion efficiencies of 6.24% and 6.94% for 10a and 4/10a, respectively.

## 2. Materials and Methods

### 2.1. Materials and Methods

All chemicals (e.g., 3-bromoaniline, chloral hydrate, N-bromosuccinimide (NBS), sodium sulfate (Na_2_SO_4_), potassium carbonate (K_2_CO_3_), potassium phosphate (K_3_PO_4_), acetic acid (AcOH), etc.), catalysts (1,1′-Bis(diphenylphosphino)ferrocene]dichloropalladium(II) [Pd(dppf)Cl_2_], tris(dibenzylideneacetone)dipalladium [Pd_2_(dba)_3_]) and organic solvents (e.g., toluene, tetrahydrofuran (THF), dimethylformamide (DMF), ethanol (EtOH) etc.) were purchased from Suna Tech Inc. (Suzhou, China) and Sigma-Aldrich (Foxburrow, MA, USA) which were used as received. Detailed synthetic procedures and characterization, NMR, UV–visible spectroscopy, and high-resolution mass spectrometry of new molecules, their intermediates, device fabrication, and current-voltage characteristics are available in the supplementary information (SI).

### 2.2. Theoretical and Computational Details

The geometries of all the molecules were fully optimized with the B3LYP functional and 6-31G(d) basis set with the polarizable continuum model (PCM) using the integral equation formalism variant (IEFPCM) in tetrahydrofuran solvent (THF, ε = 7.4257) [B3LYP/6-31G(d)/THF] [25,26]. The hexyl substituents on the N atoms of the isatylidene segments were replaced with ethyl groups to reduce the calculation cost. Frequency calculations were performed to confirm that the optimized geometries were the true energy minima. Based on the optimized geometries at the B3LYP/6-31G(d)/THF level, molecular orbital energies and electron energies were then calculated with the M06L functional and 6-31G(d) basis set with the IEFPCM solvent model in THF [M06L/6-31G(d)/THF]. Electronic absorption spectra were simulated based on the excited state data calculated with the TD–DFT method at the M06L/6-31G(d)/THF level using B3LYP/6-31G(d)/THF geometries. All calculations were carried out using the Gaussian 16 program [27,28].

## 3. Results

### 3.1. Design, Synthesis, and Characterization of A–D–A–Type OSC

**Scheme 1:**
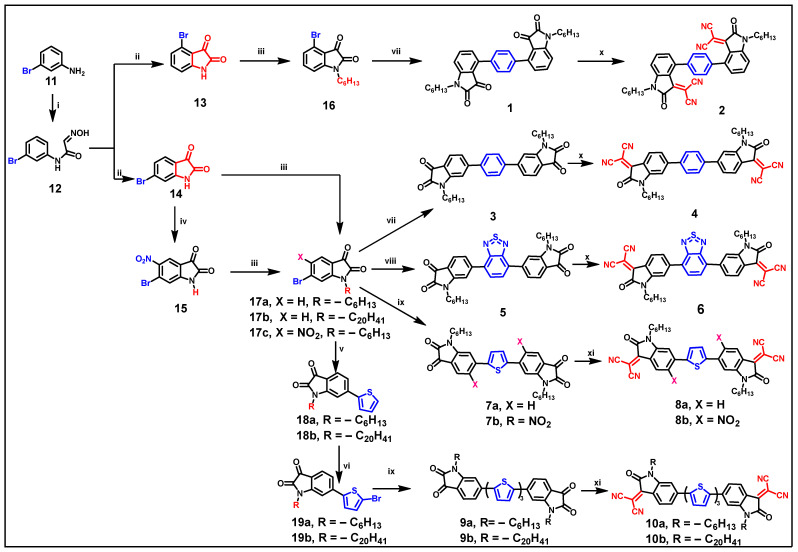
Synthetic route to bis-isatin Compounds 1, 3, 5, 7, and 9 and di-2-(2-oxindolin-3-ylidene) malononitrile derivatives 2, 4, 6, 8, and 10. Reagents and conditions: (i) chloral hydrate, Na_2_SO_4_, H_2_NOH^.^ HCl, 65 °C, 2 h; (ii) H_2_SO_4_, 75 °C, 15 min, 35% over two steps; (iii) K_2_CO_3_, Br-R, R = -C_6_H_13_ or -C_20_H_41_, DMF, 90 °C, 24 h, 80–90%; (iv) NaNO_3_, H_2_SO_4_, 0 °C, 1 h, 87%; (v) 2-tributylstannylthiophene, [Pd(dppf)Cl_2_], DMF, 90 °C, 80%; (vi) AcOH, NBS, THF, 0 °C, 5 h, 82%; (vii) 4,4,5,5-tetramethyl-2-(4-(4,4,5,5-tetramethyl-1,3-dioxolan-2-yl)phenyl)-1,3,2-dioxaborolane, P(o-tyl)_3_, [Pd_2_(dba)_3_], K_3_PO_4_, Toluene, H_2_O, 90 °C, 24 h, 60–70%; (viii) [Pd_2_(dba)_3_], 4-(4,4,5,5-tetramethyl-1,3,2-dioxaborolan-2-yl)-7-(4,4,5,5-tetramethyl-1,3-dioxolan-2-yl) benzo[c][1,2,5]thiadiazole, P(o-tyl)_3_, K_3_PO_4_, toluene, H_2_O, 90 °C, 24 h, 55%; (ix) 2,5-di-tributylstannylthiophene, [Pd(dppf)Cl_2_], DMF, 90 °C, 64–75%; (x) malononitrile, EtOH, reflux, 80–90%; (xi) malononitrile, pyridine, EtOH, rt, 85–90%.

The synthetic route to the target A–D–A–type OSC based on isatin derivatives is shown in Scheme 1. The detailed synthesis and characterization are shown in the Experimental Section of SI. The desired bromoisatins (13, 14) were synthesized through a two-step procedure followed by the synthesis of intermediate 12 using the corresponding commercial 3-bromoaniline, 11, as a starting material. To overcome the solubility issue, alkyl solubilizing side chains, hexyl or long-branched alkyl were introduced to 13 or 14 at the nitrogen atom to produce 16 or 17a–c in excellent yields. 16 and 17a were further reacted with 1,4-benzenediboronic acid bis(pinacol) ester in the presence of [Pd_2_(dba)_3_] as a catalyst and K_3_PO_4_ as a base to afford 1 and 3 in 60% and 70% yields, respectively. Subsequently, bis-isatin Compounds 1 and 3 were treated with malononitrile via a Knoevenagel condensation reaction to generate target products 2 and 4, respectively. Replacement of phenyl with electron-withdrawing benzo[c][1,2,5]thiadiazole (BTD) was targeted to analyze the effect of the π–bridge. The Suzuki–Miyaura reaction of 17a with BTD afforded 5 in 55% yield, and subsequent Knoevenagel condensation with malononitrile provided 6 in 80% yield. Introducing a thiophene ring between the acceptor units was realized under a Stille coupling reaction between 17a and 2,5-di-tributylstannylthiophene in the presence of [Pd(dppf)Cl_2_] as a catalyst and afforded 7a in 64% yield. With the aid of a catalytic amount of pyridine, the Knoevenagel condensation reaction could occur at room temperature and afforded 8a in 89% yield. It was envisaged that the introduction of a nitro group, which could be subsequently employed in a late stage in a double Cadogan cyclization, would be the optimal strategy to prepare a five-ring fused system Y6 analog. The dinitro derivatives 7b and 8b were obtained using a Stille coupling reaction followed by a Knoevenagel condensation with malononitrile. To extend the conjugation length between the two isatin moieties, terthiophene was appended to the isatin. The reaction of 2-tributylstannylthiophene with 17a or 17b under Stille coupling conditions produced asymmetrical dye 18a (or 18b) in 80% yield. Selective bromination at the 5-position of thiophene using NBS afforded intermediate 19a (or 19b). The obtained bromo-thienyl-isatin was then coupled to 2,5-di-tributylstannylthiophene to form 9a. To improve the solubility, analog 9b with a branched alkyl chain was obtained with a similar yield.

**Figure 3:**
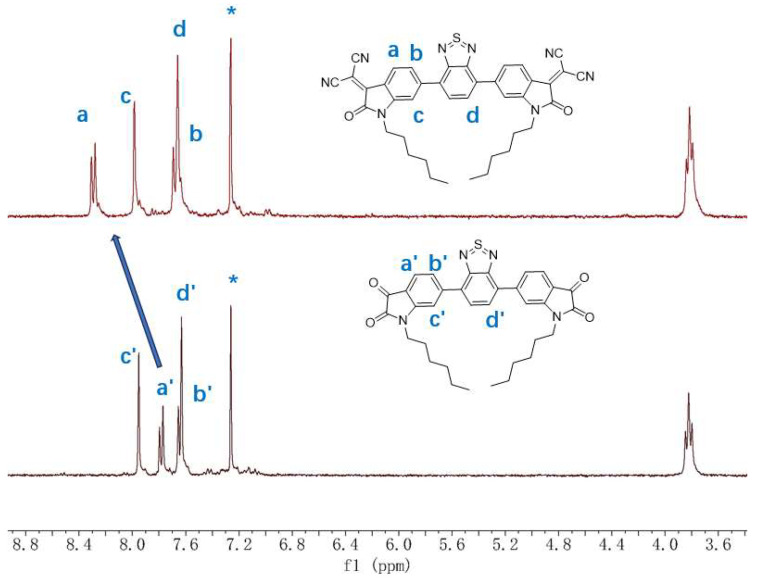
^1^H NMR spectra of Compounds 6 (**top**) and 5 (**bottom**) in CDCl_3_ (*: solvent peak).

All the newly prepared A–D–A–type OSCs based on isatin were fully characterized by ^1^H NMR, ^13^C NMR spectroscopy, and high-resolution mass spectrometry. For instance, Figure 3 provides an example of the ^1^H NMR spectra of A–D–A–type OSC Compound 5 and its malononitrile homolog 6 variants. The ^1^H NMR spectrum displays two doublet and two singlet peaks in the aromatic region, plus one at 3.80 ppm belonging to N-CH_2_, which agrees well with its chemical structure and chemical environment. After the Knoevenagel condensation reaction, the signal of Ha’ of 5 shifted from 7.78 to 8.29 ppm (Ha in 6). The Ha proton produces the most downfield signal owing to the large deshielding effect of the neighboring CN moiety (hydrogen bonding). Nevertheless, no chemical shifts were observed for the other three peaks after the condensation reaction.

### 3.2. Optical Characterization

**Figure 4:**
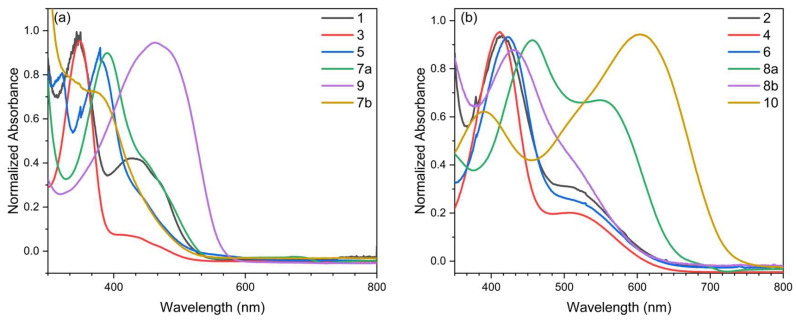
Normalized UV–vis absorption spectra of bis-isatin derivatives 1, 3, 5, 7a, 7b, and 9a,b (**a**) and di-2-(2-oxindolin-3-ylidene) malononitrile derivatives 2, 4, 6, 8a, 8b, and 10a,b (**b**) in THF (concentration range 0.05–0.10 mg/mL).

The photophysical properties of A–D–A–type OSCs based on bis-isatin and di-2-(2-oxindolin-3-ylidene) malononitrile derivatives were investigated by UV–vis spectroscopy, and the corresponding absorption data are listed in Table 1. For comparison purposes between malononitrile derivatives and bis-isatin derivatives, the absorption spectra of 1, 3, 5, 7a, 7b, 9 and their derivatives, 2, 4, 6, 8a, 8b, 10, were normalized to enable direct comparison of substituent effects (Figure 4). We note that we encountered difficulties in reproducing the molar extinction coefficients because these molecules exhibit a strong tendency to aggregate in dilute solution. Their spectra shared a common feature with two absorption bands: a low-energy band in the visible region attributed to the ICT transition arising from the acceptor moieties and a high-energy band in the range of 300–450 nm attributed to the π–π* bridge. The position of these bands was strongly influenced by the molecular structure of the compounds, the conjugation length of the π-bridge, and the nature of the acceptor moiety. The effect of the acceptor end group was demonstrated by comparing the absorption maxima of 7a and 8a. The solution spectrum of 8a exhibits bands for the π→π* transition (457 nm) and ICT (550 nm), while those of 7a are at 393 nm and 440 nm, demonstrating that the introduction of the dicyanovinylene groups resulted in a 100 nm bathochromic shift in absorption (Table 1, entries 7 and 8). A comparison of the absorption spectra of 3 (λ_max_ = 349 nm) with 7a (λ_max_ = 393 nm) revealed that the replacement of the phenyl ring with a thienyl ring caused a redshift of the high-energy band. This observation indicated that the incorporation of a thiophene moiety in A–D–A–type OSC enhanced their ICT properties, as previously reported in the literature [29,30]. This trend was also observed for the malononitrile derivatives, where a bathochromic shift of 43 nm was seen for the high-energy band. As shown in Figure S1b, the absorption spectra of both 1 and 3 exhibit two peaks assigned to the π→π* transition (350 nm) and ICT (450 nm). A comparison of the spectra of Compounds 1 and 3 shows an increase in the ICT band intensity for Compound 1. The growth of the ICT band intensity may be due to better planarity of the structure induced by an electrostatic interaction between the carbonyl of isatin and the phenyl ring forming a hydrogen bond. However, there was little variation between the main absorption peaks of the phenyl ring units at the 4- and 6-positions (Compounds 2 and 4), as shown in Figure S1c, probably because they are both linearly conjugated. This is supported by geometry optimizations where molecule 1 exhibits a more coplanar molecular conformation with a dihedral angle of 46° (Figure S2), while dicyanovinyl analog 2 exhibits a 68° dihedral angle between the phenyl and isatin planes in its most stable conformation. The H-bond (H···O) distance of 2.60 Å is significantly shorter than the sum of the van der Waals radii of O and H atoms (2.72 Å) [31]. Additionally, the possible steric hindrance of the chemical structure has always limited the introduction of a large aromatic ring at the 4-position of isatin. The maximum absorption peak of material 5 undergoes a corresponding blue shift compared to material 7a with thiophene as a bridging group. We believe that this is related to the weak electron-withdrawing ability of the BTD group and the change in the torsion angle associated with its insertion [32,33]. The absorption spectra of 10a and 10b (Figure S1d) showed almost identical absorptions in the visible range, indicating that changes in the alkyl group on the nitrogen atom hardly influenced the electronic structure of the malononitrile derivatives. For both series of bis-isatin and malononitrile derivatives, the absorption spectra revealed that the absorption peaks arising from the aromatic bridges were gradually redshifted to longer wavelengths with increasing conjugation length. For instance, 10a exhibited a broad and strong absorption spectrum with a λ_max_ of 605 nm. This redshift could be attributed to the extended π-conjugation length compared with that of 8a. Variation of the π-conjugated bridge units had a notable impact on the absorption profiles of the new bis-isatin and malononitrile derivatives. An intriguing feature observed was that 8b displayed a blueshift of the maximum absorption compared to 8a (Figure 4b, purple line vs. green line). This blueshift could be attributed to the steric hindrance of the two nitro groups (-NO_2_). This is consistent with studies by Jean et al., who reported that the decreased intensities and hypsochromic shifts in the absorption spectra of noncoplanar conjugated molecules sterically hindered by bulky nitro groups, such as 2,2′,4,4′-tetra-nitro-5,5′-dimethyl-3,3′-bithienyl; 3,3′-dinitro-5,5′-diacetyl-2,2′-bithienyl; and 3,3′-dinitro-5,5′-dicarbometh-oxy-2,2′-bithienyl, are a result of steric hindrance on the ortho-substituted groups [34]. A similar trend in the absorption features is observed when comparing the absorption bands of 7a and 7b (Figure 4a green line vs. yellow line). The optical gaps of the bis-isatin derivatives were approximately 2.25 eV, while the optical gaps of the di-2-(2-oxindolin-3-ylidene) malononitrile series were lowered to approximately 1.90 eV, with the lowest optical gap of 1.63 eV for Compound 10.

### 3.3. Electrochemical Study

**Figure 5:**
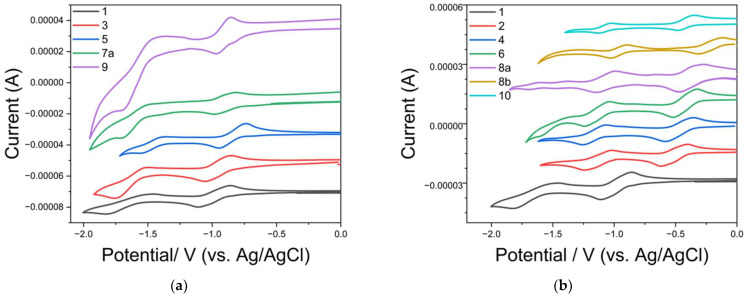
Cyclic voltammograms of bis-isatin Compounds 1, 3, 5, 7a, 9a, and 9b (**a**) and malononitrile derivatives 2, 4, 6, 8a, 8b, 10a, and 10b (**b**) in dichloromethane (concentration range of 10^−4^ to 10^−3^ M).

The electrochemical behavior of the A–D–A-type OSC based on bis-isatin and malononitrile derivatives was evaluated using CV using tetrabutylammonium perchlorate as the supporting electrolyte in dichloromethane, and the electrochemical data are listed in Table 2. The energy levels of HOMO (E_HOMO_) and LUMO (E_LUMO_) were estimated from the onset potential of the first oxidation and reduction peak vs. Fc/Fc^+^, assuming an ionization energy of 4.80 eV for ferrocene. The CVs of the bis-isatin derivatives showed one oxidation wave and two well-reversible reduction waves. The reduction potential was found to be dependent on the bis-isatin structure. The CV of di-2-(2-oxindolin-3-ylidene) malononitrile derivatives under the same conditions also showed two sets of reduction but shifted to more positive values than that of the parent compound, bis-isatin (Figure 5). For example, Compound 2 exhibited E_red1_ = −0.61 V (vs. Ag/AgCl) compared to 1 (−1.11 V), thus leading to a shift of 0.50 V. This suggests that the LUMO energy levels were predominantly controlled by the nature of the acceptor units. Regardless of the nature of the end-group substituents, both reduction potentials followed the trend Ph < BTD < Th. For instance, in the first reduction process of the A–D–A–type malononitrile family, BTD and thiophene were easier to reduce than phenyl, corresponding to peaks of −0.52 V, −0.46 V, and −0.58 V, respectively. Nevertheless, the difference in the reduction potential was less than 0.15 V, and this small change was indicative of a minor contribution of the flanking aryl groups to the LUMO (Figure 5). On the other hand, Compounds 7a and 9a,b (or Compounds 8a and 10a,b) with one thiophene and terthiophene unit as a π-bridge group presented similar LUMO energy levels. Changing the conjugate length of the π–bridge had a more significant effect on the HOMO energy level. 8b functionalized with -NO_2_ and -CN groups exhibited a low-lying LUMO energy level. The LUMO energy level could be tuned from −3.54 eV (7a) to −4.23 eV (8b) because nitro was one of the strong electron-accepting groups.

As shown in Table 2, the LUMO energy levels of the A–D–A-type OSC based on di-2-(2-oxindolin-3-ylidene) malononitrile derivatives (∼−4.0 eV) were lower than those of bis-isatin derivatives, where values of approximately −3.50 eV were measured. This is in accordance with the fact that the strong electron-withdrawing ability of the dicyanovinylene group strongly affects the LUMO energies. The HOMO levels of bis-isatin and di-2-(2-oxindolin-3-ylidene) malononitrile derivatives were estimated to be slightly above −5.50 and −5.80 eV, respectively (except Compounds 8b, 9, and 10). Changing the linkage pattern of the malononitrile derivatives (4-position to 6-position) had a small effect on the HOMO and LUMO energy levels, with a slightly higher HOMO energy level of 0.05 eV and a slightly lower LUMO energy level of approximately 0.02 eV for 4 compared to that of 2. The HOMO energy level of 8a was 0.07 eV higher than that of 4, which was caused by the stronger electron-donating ability of thiophene. In addition, the HOMO energy level of 7a was significantly higher than that of 5 (−5.35 vs. −5.53 eV, Table 2) in the bis-isatin series, which also indicated the strong electron donor characteristics of thiophene. The HOMO level of Compound 10 bearing either linear or branched alkyl side chains (10a or 10b) was much higher than that of one thiophene or one phenyl unit bridge compound due to the nature of the long conjugation donor chain. The resulting HOMO–LUMO gaps decreased to 1.20 eV. Finally, a change in the alkyl chain length does not affect the UV–visible and electrochemical properties; both series, 10a and 10b, exhibit identical HOMO/LUMO energy levels, suggesting that the influence of the alkyl chains on the frontier orbital energy level is negligible.

### 3.4. Theoretical Studies

#### 3.4.1. Molecular Orbitals

Figure S3 in SI shows the molecular orbital maps of all the molecules. Both the HOMO and LUMO are fully delocalized on the two di-2-(2-oxindolin-3-ylidene) malononitrile terminal acceptor groups and the π–conjugated bridge donor segments. The HOMOs mainly occupy the double bonds along the long-axis direction of the molecules, while the LUMOs are mostly distributed on single bonds perpendicular to the long-axis direction. For bis-isatin compounds, the distribution of HOMO and LUMO decreases from the center π–bridge to terminal A segments. In contrast, the HOMO and LUMO of malononitrile derivatives appear to distribute more on the acceptor than on the π–bridge. Table 3 summarizes the calculated HOMO and LUMO energy levels. For bis-isatin compounds, the HOMO energies have the order of 4 ≈ 6 < 8a < 10a, while the LUMO energies show a trend of 6 < 8a < 4 ≈ 10a (Table 3). The resulting HOMO–LUMO gaps decrease in the order of 4 > 6 > 8a > 10a. It is a common rule that both HOMO and LUMO increase with polymer/oligomer length, and the relatively higher HOMO and LUMO energies of 10a than 8a are easily understandable. The higher HOMO and LUMO energies of 8a than 6 indicate that the thiophene moiety has a larger electron-donating ability than the BTD group.

**Figure 6:**
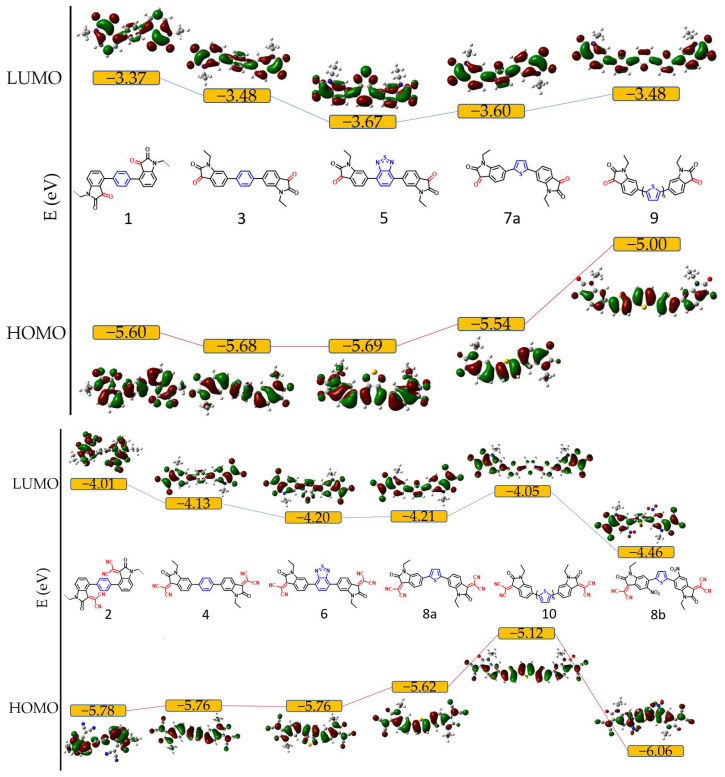
Evolution of the energy levels of the frontier molecular orbitals of five bis-isatin derivatives (**top**) and six di-2-(2-oxindolin-3-ylidene) malononitrile derivatives (**bottom**). Lines connecting the dots (different compounds) to better visualize the trends.

Figure 6 reports a survey of the calculated orbital energy levels and maps of the frontier molecular orbitals of bis-isatin and di-2-(2-oxindolin-3-ylidene) malononitrile derivatives. The higher energy levels of HOMO and LUMO of 2 than 4 may be due to the larger steric hindrance between the phenyl ring and dicyano methylene group of the isatin moiety in 2 than in 4. Substituting one carbonyl function of the isatin moiety with stronger electron-accepting dicyano methylene C(CN)_2_ in malononitrile derivatives largely decreases both the HOMO and LUMO energies and results in a significantly smaller HOMO–LUMO gap (Figure 6). Compound 8b shows lower HOMO and LUMO energies than 8a due to the introduced electron-withdrawing NO_2_ group. However, while the strong electron-accepting C(CN)_2_ in malononitrile derivatives reduces the LUMO energy more significantly than the HOMO energy and thus induces a decreased optical band gap, the nitro group in 8b reduces the HOMO energy more significantly than the LUMO energy and results in a larger HOMO–LUMO gap for 8b than 8a.

#### 3.4.2. Electronic Transitions

**Figure 7:**
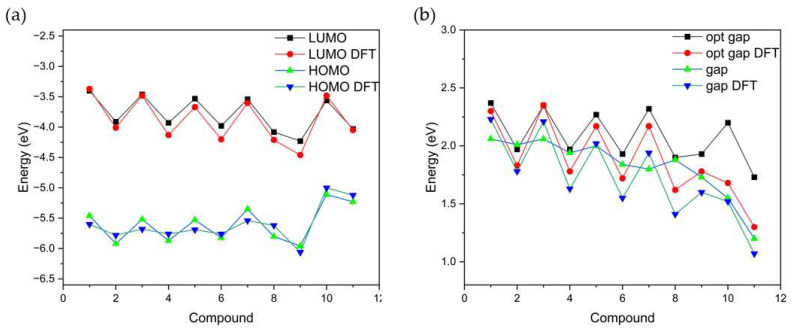
Comparison between calculated and experimental results of 11 bis-isatin and di-2-(2-oxindolin-3-ylidene) malononitrile derivatives for (**a**) HOMO and LUMO energy; (**b**) energy gap calculated by minus LUMO and HOMO energy (gap) and optical gap (opt gap) from the calculated excitation energy of the first excited state.

The computed energy levels are close to those obtained experimentally in the THF solution, indicating that M06L provides a good estimate of the HOMO–LUMO gap. The aforementioned changes in the orbital shapes and energies can be related to the differences in the electronic transitions involved in the lowest-energy and highest-energy excited states of these molecules. The calculated optical gap (excitation energy of the first excited state) has the same trend as the HOMO–LUMO gaps, but the optical gaps have larger values due to the orbital coupling energy. The lowest-energy bands are due to the electronic transition from HOMO to LUMO and could be assigned to the ICT transition between the π–conjugated bridge and end-group acceptor. ICT-type transitions generally have a weak intensity; however, when the π–conjugation length increases, the intensity of the lowest-energy band increases due to the contribution from the π–π* transition of the donor. Figure 7a shows the calculated HOMO and LUMO energies for all molecules compared to the experimental results, and further comparison of the optical band gap and electrochemically obtained energy band gaps with the simulated values is placed in Figure 7b. The calculated energy values of the HOMO and LUMO agree with the experimental data within less than 0.15 eV for the entire series of molecules, which is not the case for the optical band-gap energy. Underestimation is more severe for the larger molecules 9 and 10. The error, however, is not dramatic considering the size of the molecules. The high-energy band is attributed to the π–π* transition for both π–bridge and acceptor groups.

**Figure 8:**
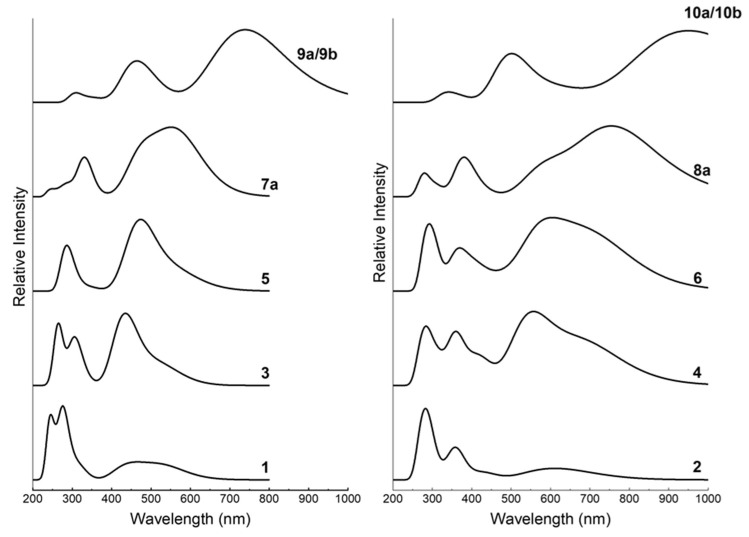
TD–DFT-simulated UV/vis absorption spectra with a half-width at half height of 2000 nm of the bis-isatin and di-2-(2-oxindolin-3-ylidene) malononitrile derivatives.

DFT-simulated UV–vis data (Figure 8) support the above observations that these lower energy transitions, which are assigned as ICT, have the maximum contribution in the visible region. In short, TD–DFT calculations were carried out to investigate the electronic effects of conjugation length and the substitution position of the end group on molecular orbitals and electronic absorption spectra. The calculated molecular orbital energies and gap correspond well with the corresponding experimental data. The deviation between the calculated and experimental orbital energies is less than 0.15 eV for most cases. In addition, the calculated trends for orbital energies among different compounds are the same as the experimental ones, as shown in Figure 7.

### 3.5. Electrical Characterization

**Figure 9:**
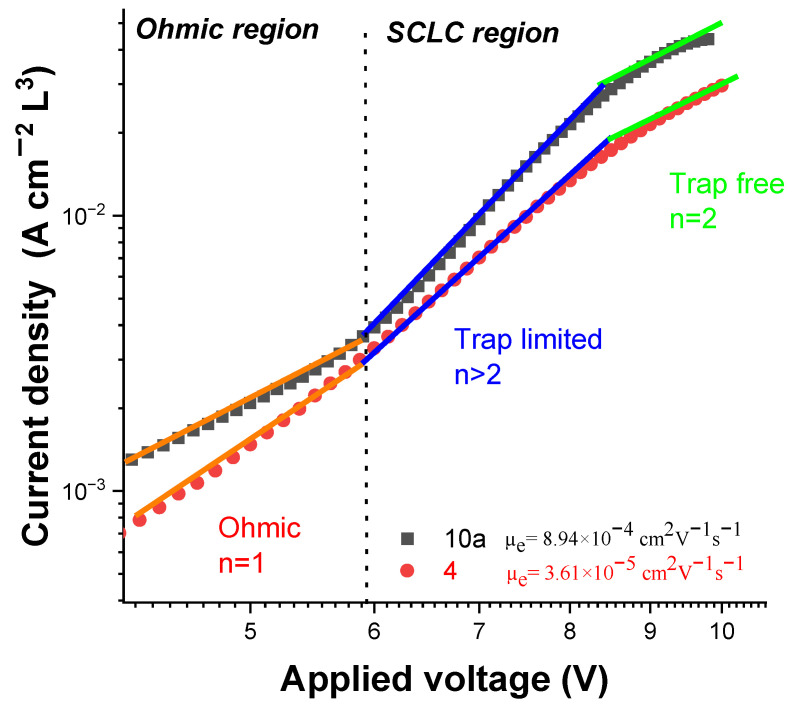
SCLC measurements of the electron-only devices for 10a and 4 semiconductors with ohmic and trap-free space charge limited current regions.

Further electronic characterization was performed by investigating the electron-transport ability of these materials. To assess their inherent charge transport properties, the electron mobilities of 4 and 10a, which differ by the length of the π–bridge between the malononitrile centers, were measured by the SCLC method and compared with those reported in the literature. The following configurations were utilized for the electron-only device: ITO/SnO_2_/active layer (10a or 4)/LiF/Al. As shown in Figure 9, the 10a or 4 molecule-based devices exhibited SCLC behavior with a clear slope variation from 4–10 V. From these *J-V* curves, the charge-carrier mobility can be extracted using the Mott-Gurney equation:

(1) $$J_{SCLC}=\frac{9}{8}\frac{\varepsilon_{0}\epsilon_{r}}{L^{3}}\mu V^{2}$$

where ε_0_ is the permittivity of the vacuum, ε_r_ is the relative permittivity of the organic compound, μ is the charge-carrier mobility, V is the voltage, and L is the thickness of the tested layer. As plotted in Figure 9, the current density-voltage characteristic curves and SCLC fittings for both films reveal the SCLC region (>6 V). This allows us to extract an average electron mobility of µ_e_ = 3.51 × 10^−4^ cm^2^ V^−1^ s^−1^ and µ_e_= 3.61 × 10^−5^ cm^2^ V^−1^ s^−1^ for 10a and 4, respectively. The highest electron mobility of 10a is 8.94 × 10^−4^ cm^2^ V^−1^ s^−1^. When compared to other A–D–A–type OSC small molecules from the literature, the mobilities of 10a and 4 are on the same order of magnitude, where bis-lactam-based materials had an OFET mobility of 2.30 × 10^−4^ cm^2^ V^−1^ s^−1^ [35].

**Figure 10:**
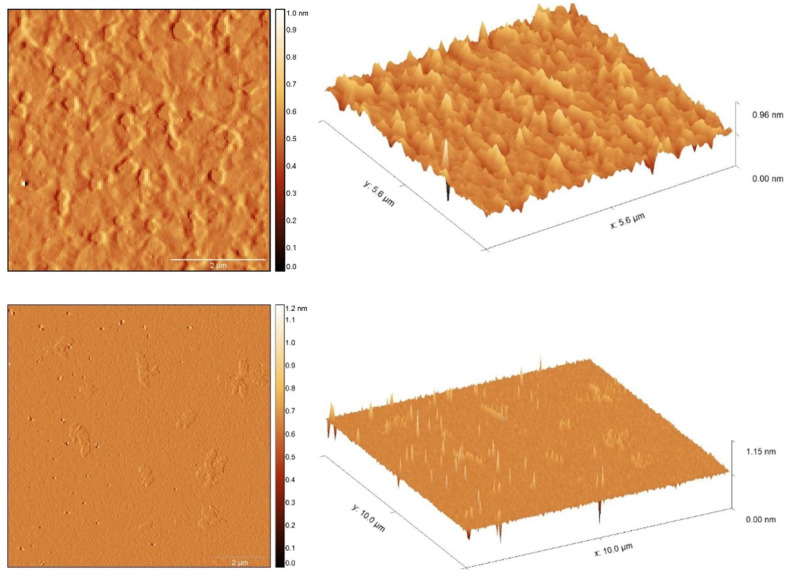
AFM topographic images of (**top**) 4 and (**bottom**) 10a thin films on silicon wafers.

Based on these results, several observations can be drawn. First, the finding that the higher mobility observed in films of 10a (A–D–A) cannot be explained only by the downshifted LUMO energy level, from −3.93 eV for 4 to −4.03 eV for 10a, which could facilitate electron injection but also by a better planarity of 10a compared to 4. A better coplanarity of the π–system due to the introduction of thiophene units may induce strong π–π stacking interactions in the solid state, leading to improved charge transport and a higher electron mobility value. Second, the finding that the electron mobility of 4 is only one order of magnitude lower than the electron mobility of 10a means that the molecular design, extending the π-conjugation core, does not greatly affect the electrical properties. As reported in the literature, charge transport, morphology, and molecular aggregation in thin films are intimately related. Although high electron mobilities have been obtained for bis-lactam-based organic materials, these values were extracted from OFET measurements. The high mobility OFET can be explained in terms of the anisotropic nature of transport in OSCs, i.e., SCLC measurements are dominated by vertical transport, while OFET measurements by horizontal transport of carriers. The surface morphologies of 10a and 4 films were examined by atomic force microscopy (AFM). As shown in Figure 10, both films exhibited a relatively smooth surface with a root mean square of 0.96 nm for molecule 4 and 1.15 nm for molecule 10a, indicating their good film-forming ability and more uniform thin film microstructures.

### 3.6. Photovoltaic Properties

**Figure 11:**
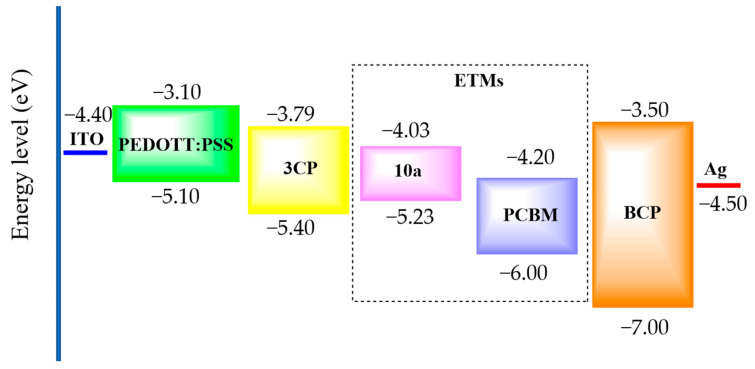
Energy band diagram of malononitrile derivative 10a material used as an ETM in the perovskite solar cell.

To demonstrate the functionality of the new acceptor materials as an active layer in optoelectronic devices, we implemented them as an electron-transport layer (ETL) in perovskite solar cells with the configuration ITO/PEDOT:PSS/triple-cation perovskite/ETM/BCP/Ag (ETM: PCBM or 10a) with 7 nm of bathocuproine (BCP) as a hole blocking layer in the ETL/Ag interface (Figure 11). We chose 10a since it has a suitable LUMO energy level of −4.03 eV as that of PCBM (−4.20 eV), which lies well below the conduction band edge of triple-cation perovskite, facilitating efficient charge extraction from the perovskite to the ETL, and has good electron mobility.

**Figure 12:**
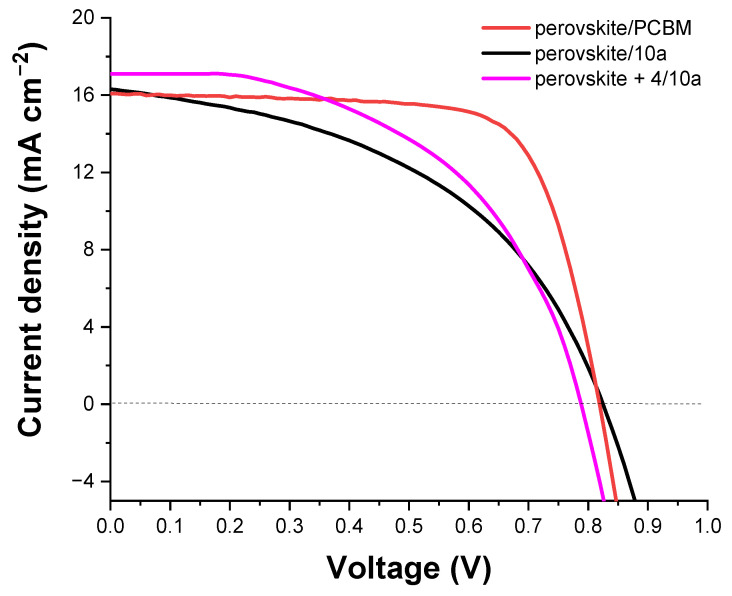
*J–V* characteristics of solar cells employing 10a (black), 4 (passive treatment)/10a (pink), and PCBM (red) as the ETL and triple-cation perovskite FA0.85Cs0.15Pb(I0.8Br0.2)3 as the absorbing layer.

The 10a solutions were optimized to a concentration of 5 mg/mL and tested for different spin-coating deposition speeds. Figure 12 shows the *J–V* curve of champion devices with 10a, 4/10a, and PCBM as an ETM. Table 4 summarizes the corresponding PV parameters.

**Figure 13:**
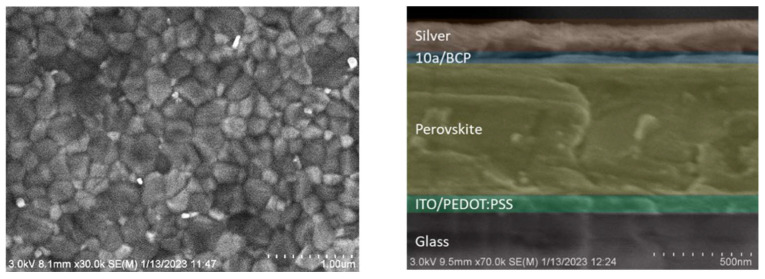
Top-view SEM images of the perovskite thin film deposited on PEDOT:PSS (**left**) and cross-sectional image of the perovskite solar cell device (**right**).

The surface morphology of the perovskite layer was examined by SEM and AFM (top-view SEM in Figure 13 and AFM images in Figure S4 in SI). The distribution of grain sizes seems more homogeneous for the perovskite film cast on ITO/PEDOT:PSS and exhibits large grain sizes in the 400–500 nm range (Figure 13a). Figure 13b displays a cross-sectional SEM image of the completed device (ITO/PEDOT:PSS/perovskite/10a/BCP/Ag) and shows the film thicknesses and morphologies of each layer. Therefore, it can be seen that a 680 nm thick perovskite layer with a smooth and flat surface is in intimate contact with the 10a layer of 55 nm and BCP of 5 nm thickness. An almost 100 nm thick ITO/PEDOT:PSS layer (the PEDOT:PSS layer is not very recognizable) is in close contact with the perovskite layer, without defects at the interface.

As per literature reports, device architecture (p-i-n), employing PCBM as ETL, ITO/PEDOT:PSS/triple-cation perovskite/PCBM/BCP/Ag shows a PCE of approximately 11% [36,37]. Our best control device with a PCBM layer delivered a PCE of 9.44%, with a V_OC_ of 0.81 V, a J_SC_ of 16.70 mA cm^−2^, and an FF of 73%, which is inferior to that reported in other studies [38,39]. The 10a-based device showed a PCE of 6.24% with a J_SC_ of 16.30 mA cm^−2^, a V_OC_ of 0.82 V, and an FF of 47%. 10a showed low performance with low FF compared to the PCBM one, which could be related to several factors, such as a higher mobility of PCBM (µ_PCBM_ = 1.22 × 10^−3^ cm^2^ V^−1^ s^−1^) compared to that of 10a (µ_10a_ = 1.09 × 10^−4^ cm^2^ V^−1^ s^−1^). One of the reasons for the low values of Voc and FF for both devices could be related to nonradiative recombination. Generally, it has been agreed that the nonradiative recombination losses arise from defects (charge-carrier traps) either in the bulk perovskite or at the interfaces [40]. Therefore, an optimization effort to passivate the defects on the perovskite surfaces and at the grain boundaries is necessary to enhance efficiency. Previous work has demonstrated that suitable small molecules, such as D–A–D with malononitrile as the acceptor moiety, can effectively passivate the perovskite via Pb–N interactions [41,42]. To verify the passivation effect, we prepared samples: pristine perovskite film and perovskite complexed with 4 via a solvent annealing process. Compared with a control cell, the devices incorporating derivative 4 exhibited improved performance with PCE = 6.94%, Voc = 0.79 V, Jsc = 17.11 mA cm^−2^, and FF = 51%. Thus, passivation treatment improved the Jsc and FF and confirmed less nonradiative recombination in the perovskite films. The overall improvement in the PCE should be ascribed to the ability of derivative 4 to passivate various trap sites on the crystal surface and at the grain boundaries of perovskite to minimize nonradiative charge recombination. In summary, this unoptimized device was operational with a PCE of approximately 7%. Although this initial device displays a lower PCE compared with a reference device using PCBM (PCE of 9%), this work highlights the potential of these materials to act as ETLs in solar cells.

## 4. Conclusions

In this study, we provide a wide range of systematic structure-property relationships that offer new perspectives on the design of electron-acceptor materials. Appropriate chemical modifications of both π–bridges and end-capped groups can effectively manipulate the optical, electrochemical, and energy levels of these molecules. When the isatin ring was replaced by the isatylidene malononitrile ring, a bathochromic shift of 80–110 nm at the longest wavelength was observed, which reduced the optical band gap. The redshift induced by thiophene is more pronounced than that induced by the BDT or phenyl moiety, suggesting that thiophene has stronger intramolecular CT properties. The LUMO energy level of the malononitrile materials obtained by electrochemical measurements is approximately −4.00 eV, which is approximately 0.5 eV lower than that of bis-isatin derivatives. Changing the bridging group affects both the HOMO and LUMO energy levels of the materials, unlike the traditional view in which the donor unit mainly affects the HOMO energy level of the materials. This is also reflected in the DFT calculations, where both HOMO and LUMO are fully delocalized on the two malononitrile terminal acceptor groups and the π–conjugated bridge donor segments. Meanwhile, the HOMO or LUMO energy levels of the materials obtained by DFT calculations are very close to the experimental data, with many data deviations within 0.1 eV. Material 10a has a low LUMO energy level, near −4.0 eV, as well as a small band gap of approximately 1.20 eV. The introduction of a nitro group in dicyano bis-isatin significantly stabilizes the LUMO energy level and thus may contribute to the air stability of the material. The ability of these new A–D–A OSC to transport electrons is confirmed by SCLC measurements. Furthermore, their utility as electron-transport materials is demonstrated in PSCs.

## Figures and Tables

**Table 1:** Optical properties analysis data of synthesized bis-isatin and di-2-(2-oxindolin-3-ylidene) malononitrile derivatives 1–10.

Compounds	λ_max_ ^sol^ (nm) ^a^	λ_onset_ ^sol^ (nm) ^a^	E_g_^opt^ (eV) ^b^
1	430, 347	530	2.34
2	514, 415	628	1.97
3	434, 349	532	2.33
4	515, 414	630	1.97
5	440, 380	546	2.27
6	533, 423	651	1.90
7a	440, 393	553	2.24
7b	375	526	2.35
8a	550, 457	670	1.85
8b	432, 520	642	1.93
9a,b	462	586	2.11
10a,b	605, 383	758	1.63

^a^ λ_max_ in THF solution, ^b^ the optical energy gap is estimated from the absorption onset in THF solution E_g_^opt^ = 1240/λ_onset_.

**Table 2:** Cyclic voltammetry results of bis-isatin and di-2-(2-oxindolin-3-ylidene) malononitrile derivatives 1–10.

	E_red_	E_red_^onset^	E_LUMO_	E_HOMO_	E_g_^cv^
	(V) ^1^	(V)	(eV) ^2^	(eV) ^2^	(eV) ^3^
1	−1.11	−0.87	−3.40	−5.46	2.06
2	−0.61	−0.36	−3.91	−5.92	2.01
3	−1.03	−0.81	−3.46	−5.52	2.06
4	−0.58	−0.34	−3.93	−5.87	1.94
5	−0.93	−0.74	−3.53	−5.53	2.00
6	−0.52	−0.29	−3.98	−5.82	1.84
7a	−0.94	−0.73	−3.54	−5.35	1.81
8a	−0.46	−0.19	−4.08	−5.80	1.88
8b	−0.30	−0.04	−4.23	−5.96	1.73
9a,b	−0.95	−0.71	−3.56	−5.11	1.55
10a,b	−0.47	−0.24	−4.03	−5.23	1.20

^1^ reduction peck; ^2^ HOMO/LUMO energy levels were calculated from the onset of oxidation and reduction waves using an offset of −4.80 eV for saturated Ag/AgCl, E_LUMO_ = −4.80 eV – [(Eredonset) – E_1/2_(ferrocene)] and E_HOMO_ = −4.80 eV – [(E_ox_^onset^) – E_1/2_(ferrocene)], ^3^ E_g_^cv^= electrochemical gap.

**Table 3:** Calculated energies of the HOMO and the LUMO (E_HOMO_^DFT^ and E_LUMO_^DFT)^, HOMO–LUMO energy gap (E_g_^DFT^), optical gap (E_g-opt_^DFT^, excitation energy of the first excited state.

	E_g-opt_^DFT^	E_LUMO_^DFT^	E_HOMO_^DFT^	E_g_^DFT^
	(eV)	(eV)	(eV)	(eV)
1	2.30	−3.37	−5.60	2.23
2	1.83	−4.01	−5.78	1.77
3	2.35	−3.48	−5.68	2.20
4	1.78	−4.13	−5.76	1.63
5	2.17	−3.67	−5.69	2.02
6	1.72	−4.20	−5.76	1.56
7a	2.17	−3.60	−5.54	1.94
8a	1.62	−4.21	−5.62	1.41
8b	1.78	−4.46	−6.06	1.60
9a,b	1.68	−3.48	−5.00	1.52
10a,b	1.30	−4.05	−5.12	1.07

At the M06L/6-31G(d) level with the IEFPCM solvent model in THF based on the optimized geometries at the B3LYP/6-31G(d) level with the IEFPCM solvent model in THF.

**Table 4:** PV parameters of PSCs using PCBM or 10a, 4 (passive treatment)/10a as ETMs.

ETM	J_sc_ [mA cm^−2^]	V_oc_ [V]	FF [%]	PCE [%]
PCBM	16.70	0.81	73	9.44
	(15.85)	(0.80)	(72)	(9.05)
10a	16.30	0.82	47	6.24
	(14.50)	(0.78)	(46)	(5.34)
4/10a	17.11	0.79	51	6.94
	(17.53)	(0.81)	(50)	(6.78)

Data in parentheses are the average over 8 for PCBM, 6 for 10a, and 5 for 4/10a.

## Data Availability

Not applicable.

## Supplementary Materials

The following supporting information can be downloaded at: https://www.mdpi.com/article/10.3390/ma16196410/s1, Figure S1: Normalized UV–vis absorption spectra of (a) Compound 7a and 8a in THF; (b) bis-isatin Compounds 1 and 3 in THF; (c) di-2-(2-oxindolin-3-ylidene) malononitrile Compounds 2 and 4 in THF; (d) di-2-(2-oxindolin-3-ylidene) malononitrile Compounds 10a and 10b in THF; Figure S2: The lowest-energy configuration of Compound 1 and 2 obtained by relying on DFT at the B3LYP-D3/def2tzvp level; Figure S3: HOMO/LUMO maps of bis-isatin and di-2-(2-oxindolin-3-ylidene) malononitrile derivatives; Figure S4: AFM image of PSCs device (a) and SCLC device for measuring the electron mobility (b); Figures S5–S22: Mass spectra.
