# Supplementary material for: Tuning the Photophysical Properties of Acceptor–Donor–Acceptor Di-2-(2-oxindolin-3-ylidene) Malononitrile Materials via Extended π–Conjugation: A Joint Experimental and Theoretical Study
Source: Materials (Basel). 2023 Sep 26;16(19):6410. doi: 10.3390/ma16196410 (PMC10573274; doi:10.3390/ma16196410)
Supplement: Supplementary file 1 [file materials-16-06410-s001.zip › materials-2628229-supplementary.pdf]

Supplementary Information

Tuning Photophysical Properties of Acceptor–Donor–Acceptor Di-2-(2-oxindolin-3-ylidene) Malononitrile Materials via Extended π–Conjugation: A Joint Experimental and Theoretical Study

Shiwei Ren, Amirhossein Habibi, Pingping Ni, Yuexing Zhang, Abderrahim Yassar

Table of contents

1. General procedures and experimental details
2. Synthetic procedures and characterization
3. Figure of S1
4. Figure of S2
5. Figure of S3
6. Device fabrication
7. Figure of S4
8. Mass spectra (Figure S5-S22)
9. ^{1}H and ^{13}C NMR spectra

1. General procedures and experimental details

Chemical reagents were purchased from Aldrich, Alfa Aesar and used as received. Reactions with air-sensitive materials were carried out under an argon atmosphere. Flash chromatography was performed using silica gel (Merck, Kieselgel 60, 230–240 mesh). Analytical thin layer chromatography (TLC) was performed using aluminum-coated Merck Kieselgel 60 F254 plates. Known compounds, **13**, **14**, **15**, **16**, **17a**, **c**, were synthesized following the protocol reported in the literature and they gave identical spectroscopic data to those previously described in reference. Otherwise, all new compounds gave satisfactory spectroscopic data, ^{1}H NMR, ^{13}C NMR spectroscopy and high-resolution mass spectrometry in accordance to their proposed structures. ^{1}H-NMR and ^{13}C-NMR spectra were collected on a Bruker ARX 300 NMR spectrometer with deuterated solvents and tetramethylsilane (δ = 0 ppm) as the internal standard. Coupling constants (J) are denoted in Hz and chemical shifts (δ) in parts per million (ppm). Multiplicities are denoted as: s = singlet, d = doublet, t = triplet, m = multiplet, br = broad singlet. Mass spectrometry was performed by the "Fédération de Recherche" ICOA/CBM (FR2708) platform of Orléans in France. High-resolution ESI mass spectra (HRMS) were performed on a Bruker maXis Q-TOF in the positive ion mode. The analytes were dissolved in a suitable solvent at a concentration of 1 mg/mL and diluted 500 times in methanol (≈ 2 ng/mL). The diluted solutions (0.2 µL) were delivered to the ESI source by a Dionex Ultimate 3000 RSLC chain used in FIA (Flow Injection Analysis) mode at a flow rate of 200 µL/min with a mixture of CH_3CN/H_2O+0.1% of HCO_2H (65/35). ESI conditions were as follows: capillary voltage was set at 4.5 kV; dry nitrogen was used as nebulizing gas at 0.6 bar and as drying gas set at 200°C and 7.0 L/min. ESI-MS spectra were recorded at 1 Hz in the range of 50-3000 m/z. Calibration was performed with ESI-TOF Tuning mix from Agilent and corrected using lock masses at m/z 299.294457 (methyl stearate) and 1221.990638 (HP-1221). Data were processed using Bruker Data Analysis 4.4 software. UV-vis spectra were recorded on a Varian Cary model 500 UV-Vis-NIR spectrophotometer using standard quartz cells of 1 cm width and solvents of spectroscopic grade. Electrochemical measurements were carried out in dichloromethane solution with tetrabutylammonium perchlorate using a Metrohm Autolab PGSTAT12 Potentiostat. A three-electrode system was used with glassy carbon as a working electrode, a Pt wire as the counter electrode and reference electrode of saturated Ag/AgCl.

2. Synthetic procedures and characterization

***4-bromoindoline-2,3-dione (13)*:**

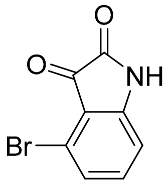

13 was synthesized following a two-step protocol published in reference (1). Step one: synthesis of isonitrosoacetanilide. Chloral hydrate (4.5 g, 27 mmol, 1.12 eq) and Na_2SO_4 (12 g, 84 mmol, 3.5 eq) were dissolved in water (60 ml) in a 250 ml three-neck flask and warmed to 35 ℃. A warm solution of the ***11*** (4.2 g, 24 mmol) in 20 ml of water, and an aqueous solution of concentrated HCl (2.6 ml) was added, a white precipitate of the amine sulfate was formed, followed by a warm solution of hydroxylamine hydrochloride (5.5g, 79 mmol, 3.3 eq) in 25 ml water. The mixture was stirred and heated at 80 ℃ for 2 h and then allowed to cool to room temperature. Filtered the yellow cream product and washed it with water. Dry overnight afforded the intermediate ***12***. The crude product was directly used in the next step without further purification. Step two: synthesis of 4-bromoindoline-2,3-dione. Conc sulfuric acid (50 ml) was heated in a 100 ml flask at 60 ℃. The dry isonitrosoacetanilide was added in small portion with stirring over 30 mins so that the temperature did not exceed 70 ℃. The mixture was then heated to 80 ℃ for 10 mins, allowed to cool to room temperature. The reaction mixture is cooled on ice and left to stand overnight. The orange-red precipitate was filtered off and washed with water. The crude product was a mixture of ***13*** and ***14***. The mixture was dissolved in a solution of NaOH (0.7 g) in 7.0 ml of water at 60 ℃ and then acidified with AcOH (3.0 ml). Then, the reaction mixture was standed for 1 h and cooled to room temperature. The precipitate was collected by filtration and washed with water to afford desired products ***13*** as an orange powder (43% yield over two steps).

^{1}H NMR (DMSO-d_6, 300 MHz, ppm) δ 11.19 (br, 1H, N-H), 7.46 (t, *J* = 8.0 Hz, 1H, Ar-H), 7.23 (d, *J* = 8.0 Hz, 1H, Ar-H), 6.89 (d, *J* = 7.8 Hz, 1H, Ar-H).

***6-bromoindoline-2,3-dione (14)*:**

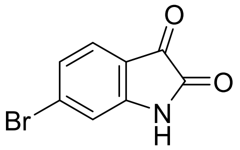

14 was synthesized following a two-step protocol published in reference (1). Step one: synthesis of isonitrosoacetanilide. Conc H_2SO_4 (50 ml) was heated in a 100 ml flask at 60 ℃. The dry isonitrosoacetanilide was added in small portion with stirring over 30 mins so that temperature did not exceed 70 °C. The mixture was then heated to 80 °C for 10 mins, then cooled to room temperature. The reaction mixture was cooled on ice and left to stand overnight. The orange-red precipitate was filtered off and washed with water. The crude product was a mixture of 4-bromoindoline and 6-bromoindoline. Then the mixture was dissolved in a solution of NaOH (0.7 g) in 7.0 ml of water at 60 °C and then acidified with acetic acid (3.0 ml). The solution was standed for 1 h to allow precipitation of solid. The resulting solid was filtered and the combined filtrate was acidified with concentrated HCl solution (6.0 mL) and standed for 2 h, then the precipitate was filtered off and washed with water. The obtained solid was dried overnight under vacuum to afford the desired product ***14*** as an orange-red powder (35% yield over two steps).

^{1}H NMR (300 MHz, DMSO-*d*$_6$) δ 11.14 (br, 1H, N---H), 7.41 (dd, *J* = 7.9, 2.5 Hz, 1H, Ar-H), 7.24 (d, *J* = 8.7 Hz, 1H, Ar-H), 7.06 (s, 1H, Ar-H).

***6-bromo-5-nitroindoline-2,3-dione (15)*:**

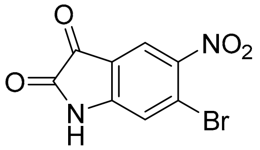

15 was synthesized using the protocol published in reference (1). To a solution of $NaNO_3$ (188 mg, 2.21 mmol) in concentrated H_2SO_4 (4 mL) was added dropwise a solution of ***14*** (499 mg, 2.21 mmol) in concentrated H_2SO_4 (3 mL) at 0 °C. After 2h, the mixture was cooled, the reaction mixture was then poured into ice water (50 mL), and the precipitate was collected by filtration and washed with water three times to afford ***15*** as a yellow solid (598 mg, 82%).

^{1}H NMR (300 MHz, DMSO-*d*$_6$) δ 11.57 (br, 1H, N-H), 8.15 (s, 1H, Ar-H), 7.29 (s, 1H, Ar-H).

***4-bromo-1-hexylindoline-2,3-dione (16)*:**

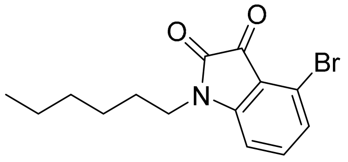

16 was synthesized using the protocol published in reference (1). In an oven-dried three-necked, round-bottomed flask equipped with a magnetic stir bar, compound ***13*** (1.0 g, 4.42

mmol) and freshly dried potassium carbonate (K_2CO_3, 0.73 g, 5.28 mmol, 1.2 eq) were dissolved in 15 ml of anhydrous dimethylformamide (DMF) under argon atmosphere. The mixture was heated for 30 mins to slight reflux and a solution of 0.74 ml (5.27 mmol, 1.2 eq) of 1-bromohexane was added dropwise via syringe. After complete dropwise addition the reaction was stirred and heated at 100 °C for 18 h. The progress of the reaction was monitored by thin layer chromatography (TLC). The DMF was removed under vacuum distillation and ethyl acetate was added. The organic phase was washed with brine 3 times and dried over anhydrous Na_2SO_4. The solvents were evaporated on a rotary evaporator and the residue was chromatographed on silica gel (chloroform/hexane = 1:2) to afford ***16*** as an orange-red solid (1.22g, 89% yield).

^{1}H NMR ($CDCl_3$, 300 MHz, ppm) δ 7.40 (td, *J* = 8.0, 1.5 Hz, 1H, Ar-H), 7.24 (d, *J* = 7.8, 1H, Ar-H), 6.84 (dd, *J* = 7.9, 1.4 Hz, 1H, Ar-H), 3.72 (t, *J* = 7.4, 2H, N-CH_2), 1.74-1.61 (m, 2H, -CH_2), 1.45-1.25 (m, 6H, -CH_2), 0.90 (t, *J* = 6.4 Hz, 3H, -CH_3).

^{13}C NMR ($CDCl_3$, 75 MHz, ppm) δ 180.8, 156.9, 152.4, 138.8, 127.9, 120.9, 115.7, 109.4, 40.2, 31.2, 27.0, 26.4, 22.4, 13.9.

***6-bromo-1-hexylindoline-2,3-dione (17a)*:**

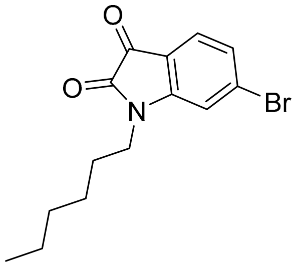

17a was prepared from ***14*** by a similar synthetic procedure analogous to that of **16**. Compound ***17a*** was an orange-red solid (81% yield).

^{1}H NMR ($CDCl_3$, 300 MHz) δ 7.45 (d, *J* = 7.9 Hz, 1H, Ar-H), 7.27 (d, *J* = 7.7 Hz, 1H, Ar-H), 7.05 (s, 1H, Ar-H), 3.68 (t, *J* = 7.4 Hz, 2H, N-CH_2), 1.72-1.61 (m, 2H, -CH_2), 1.40-1.30 (m, 6H, -CH_2), 0.88 (t, 3H, *J* = 6.3 Hz, -CH_3).

^{13}C NMR (75 MHz, CDCl3) δ 182.4, 157.9, 151.8, 133.5, 126.8, 126.4, 116.2, 113.8, 40.5, 31.3, 27.1, 26.5, 22.5, 13.9.

***6-bromo-1-hexyl-5-nitroindoline-2,3-dione (17c)*:**

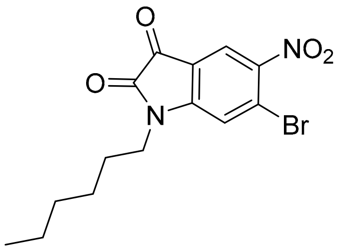

17b was synthesized using by a similar synthetic procedure analogous to that of **16**. Compound ***17c*** was a yellow solid. (680 mg, 87%).

^{1}H NMR ($CDCl_3$, 300 MHz) δ 8.19 (s, 1H, Ar-H), 7.26 (s, 1H, Ar-H), 3.77 (t, J = 7.4 Hz, 2H, N-CH_2), 1.75-1.67 (m, 2H, -CH_2), 1.40-1.35 (m, 6H, -CH_2), 0.90 (t, 3H, J = 6.3 Hz, -CH_3).

***6-bromo-1-(2-octyldodecyl) indoline-2,3-dione (17b)*:**

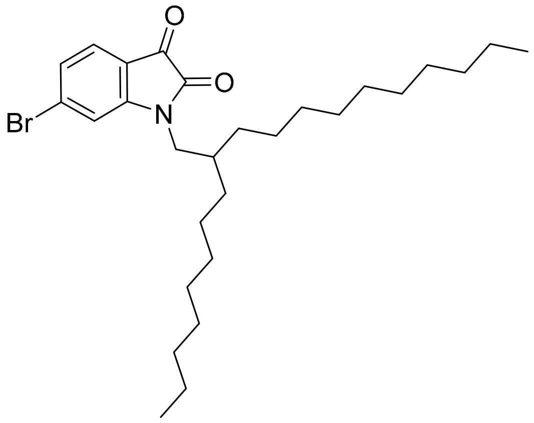

In an oven-dried round-bottomed flask equipped with a magnetic stir bar, compound ***14*** (1.39 g, 6.18 mmol) and freshly dried K_2CO_3 (1.70 g, 12.36 mmol, 2 eq) were dissolved in 50 ml of freshly distilled DMF under argon atmosphere. The mixture was warmed at 90 °C for 30 mins and then 2.68 g (7.41 mmol, 1.2eq) of 9-(bromomethyl)nonadecane was added one portion via syringe. The reaction mixture was stirred for overnight at 90 °C. The solution was cooled to room temperature and DMF was removed under vacuum distillation. Then ethyl acetate was added and the organic layer was washed 3 times with water. The organic phase was dried over anhydrous Na_2SO_4 and the solvents were removed by rotational evaporation, the residue was then chromatographed on silica gel (chloroform/hexane = 1:5) to afford ***17b*** as an orange-red solid (2.65g, 85% yield).

^{1}H NMR ($CDCl_3$, 300 MHz) δ 7.46 (dd, J = 8.0, 2.2 Hz, 1H, Ar-H), 7.27 (d, J = 7.3 Hz, 1H, Ar-H), 7.02 (s, 1H, Ar-H), 3.58 (d, J = 6.7 Hz, 2H, N-CH_2), 1.89-1.76 (m, 1H, N-CH_2-CH_1), 1.40-1.18 (m, 32H, -CH_2), 0.93 – 0.80 (m, 6H, -CH_3).

^{13}C NMR ($CDCl_3$, 75 MHz,) δ 182.3, 158.3, 152.3, 133.5, 126.8, 126.3, 116.2, 114.0, 44.9, 35.9, 31.9,

31.9, 31.4, 29.9, 29.6, 29.6, 29.5, 29.4, 29.3, 26.3, 22.7, 22.7, 14.1.

HRMS (ESI) calculated for $C_{28}H_{45}BrNO_2^+$: 506.2628; Found: 506.2633.

1-hexyl-6-(thiophen-2-yl) indoline-2,3-dione (18a):

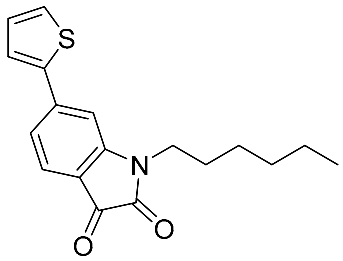

The compound ***17a*** (1 g, 3.22 mmol) and 2-(tributylstannyl) thiophene (1.68 g, 4.5 mmol, 1.4 eq), were loaded under argon into flask, then an anhydrous DMF (5 mL) was added. After stirring under argon at rt during 0.5 h, Pd(dppf)Cl_2 (71 mg, 0.09 mmol, 2%) was added and the solution was warmed to 100 °C. The resulting mixture was stirred at 110 °C overnight. The DMF was removed under vacuum distillation, and the residue was extracted with ethyl acetate. The organic phase was combined and washed with brine, water, and then dried with anhydrous Na_2SO_4. The solvents were removed by rotational evaporation, the residue was chromatographed on silica gel (chloroform/hexane = 1:2) to afford ***18a*** as a red solid (0.81g, 80% yield).

^{1}H NMR ($CDCl_3$, 300 MHz) δ 7.60 (d, *J* = 7.9 Hz, 1H, Ar-H), 7.51-7.45 (m, 2H, Ar-H), 7.34 (d, *J* = 7.8 Hz, 1H, Ar-H), 7.17 (t, *J* = 3.9 Hz, 1H, Ar-H), 7.05 (s, 1H, Ar-H), 3.76 (t, *J* = 7.6 Hz, 2H, N-CH_2), 1.78-1.67 (m, 2H, -CH_2), 1.42-1.28 (m, 6H, -CH_2), 0.91-0.86 (m, 3H, -CH_3).

^{13}C NMR ($CDCl_3$, 75 MHz) δ 182.4, 158.7, 151.8, 144.0, 142.6, 128.7, 128.1, 126.2, 126.1, 120.7, 116.2, 106.8, 40.2, 31.4, 27.3, 26.6, 22.5, 14.0.

HRMS (ESI) calculated for $C_{18}H_{20}NO_2S^+$: 314.1206; Found: 314.1209.

1-(2-octyldodecyl)-6-(thiophen-2-yl) indoline-2,3-dione *(18b):*

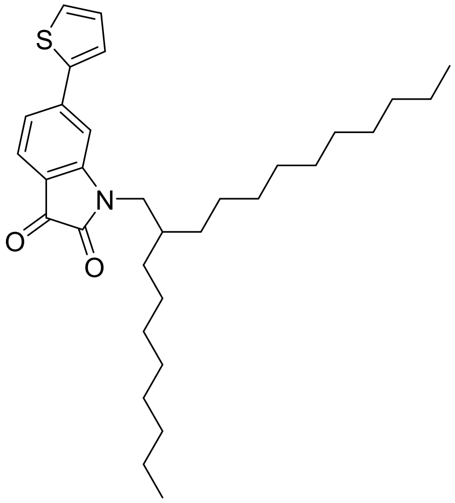

18b was prepared from ***17c*** by a similar synthetic procedure analogous to that of **18a**. Chromatography on silica gel (chloroform/hexane = 1:3) afford to ***18b*** as a red solid (1.15 g, 82% yield).

^{1}H NMR ($CDCl_3$, 300 MHz) δ 7.61 (d, *J* = 7.9 Hz, 1H, Ar-H), 7.52-7.44 (m, 2H, Ar-H), 7.35 (d, *J* = 7.7 Hz, 1H, Ar-H), 7.17 (td, *J* = 3.9, 2.0 Hz, 1H, Ar-H), 7.06 (s, 1H, Ar-H), 3.64 (d, *J* = 7.3 Hz, 2H, N-CH_2), 1.93-1.83 (m, 1H, N-CH_2-CH_1), 1.32-1.20 (m, 32H, -CH_2), 0.93-0.88 (m, 6H, -CH_3).

^{13}C NMR ($CDCl_3$, 75 MHz) δ 182.4, 159.0, 152.2, 143.9, 142.6, 128.7, 128.1, 126.2, 125.9, 120.5, 116.2, 107.1, 44.6, 36.2, 31.9, 31.9, 31.6, 29.9, 29.7, 29.6, 29.4, 29.3, 26.5, 22.7, 14.2.

HRMS (ESI) calculated for $C_{32}H_{48}NO_2S^+$: 510.3402; Found: 510.3400.

6-(5-bromothiophen-2-yl)-1-hexylindoline-2,3-dione (19a):

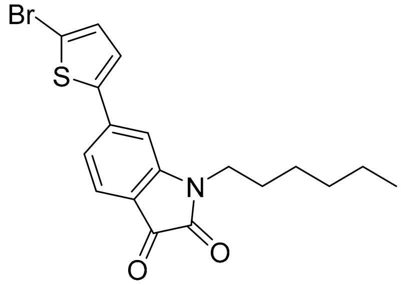

In a round-bottom flask, under argon, the ***18a*** (50 mg, 0.16 mmol) in THF (3 mL) was added portionwise NBS (32 mg, 0.17 mmol, 1.05 eq) at 0 °C. Then, a catalytic amount of AcOH was added and the suspension was warmed up to room temperature and stirred for 5 h. Water was then added and the solution was extracted three times with dichloromethane. The combined organic layers were dried over anhydrous Na_2SO_4. The solvent was then removed under reduced pressure and the crude product was further purified by chromatographed on silica gel (chloroform/hexane = 1:2 to 1:1) to afford ***19a*** as an orange solid (51mg, 82% yield).

^{1}H NMR ($CDCl_3$, 300 MHz) δ 7.60 (d, *J* = 7.9 Hz, 1H, Ar-H), 7.26-7.21 (m, 2H, Ar-H), 7.12 (d, *J* = 3.7 Hz, 1H, Ar-H), 6.94 (s, 1H, Ar-H), 3.75 (t, *J* = 7.7 Hz, 2H, N-CH_2), 1.75-1.65 (m, 2H, -CH_2), 1.42-1.28 (m, 6H, -CH_2), 0.92-0.85 (m, 3H, -CH_3).

^{13}C NMR ($CDCl_3$, 75 MHz) δ 182.3, 158.6, 151.8, 143.8, 142.9, 131.6, 126.3, 126.2, 120.3, 116.5, 115.3, 106.4, 40.3, 31.4, 27.3, 26.6, 22.5, 14.0.

HRMS (ESI) calculated for $C_{18}H_{19}B_rNO_2S^+$: 392.0315; Found: 392.0314.

***6-(5-bromothiophen-2-yl)-1-(2-octyldodecyl) indoline-2,3-dione (19b)*:**

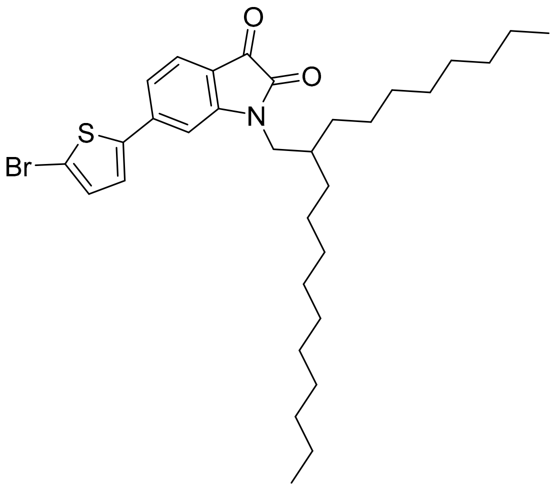

This compound was prepared from ***18b*** by a similar synthetic procedure analogous to that of **19a**. Chromatography on silica gel (chloroform/hexane = 1:4 to 1:3) afford to ***19b*** as an orange-red solid (310 mg, 81% yield).

^{1}H NMR ($CDCl_3$, 300 MHz) δ 7.60 (d, *J* = 7.9 Hz, 1H, Ar-H), 7.26-7.25 (m, 2H, Ar-H), 7.12 (d, *J* = 4.0 Hz, 1H, Ar-H), 6.94 (s, 1H, Ar-H), 3.63 (d, *J* = 7.3 Hz, 2H, N-CH_2), 1.93-1.82 (m, 1H, N-CH_2-CH_1), 1.44-1.20 (m, 32H, -CH_2), 0.87 (t, *J* = 7.2 Hz, 6H, -CH_3).

^{13}C NMR ($CDCl_3$, 75 MHz) δ 182.3, 158.9, 152.3, 143.9, 142.9, 131.6, 126.2, 126.1, 120.1, 116.5, 115.3, 106.7, 44.6, 36.2, 31.9, 31.9, 31.6, 29.9, 29.7, 29.6, 29.6, 29.4, 29.3, 26.5, 22.7, 22.7, 14.1.

HRMS (ESI) calculated for $C_{32}H_{47}BrNO_2S^+$:588.2505; Found: 588.2505.

***4,4′-(1,4-phenylene) bis (1-hexylindoline-2,3-dione) (1)*:**

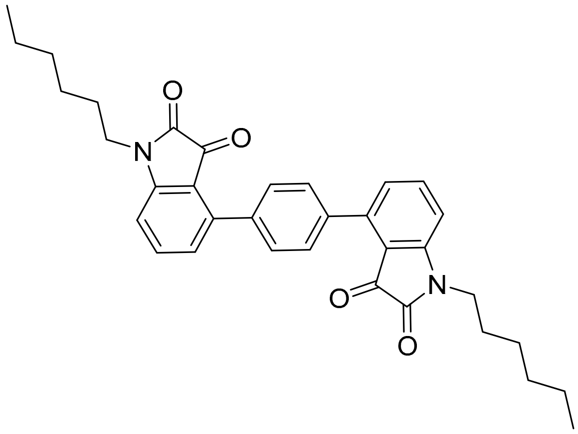

In a round-bottom flask, compound ***16*** (364 mg, 1.17 mmol), 1,4-Benzenediboronic acid bis (pinacol) ester (193 mg, 0.58 mmol, 0.5 eq), P(o-tyl)$_3$ (50 mg, 0.17mmol, 10%) and K_3PO_4 (747 mg, 3.52 mmol, 3 eq) were loaded under argon. A solution of toluene (5 mL) and water (0.5 ml) was then added to the flask. After stirring in rt for 0.5 h, $Pd_2(dba)_3$ (50 mg, 0.05 mmol, 4%) was added and the resulting mixture was heated to 100 °C for overnight. Then, the solvents were removed under vacuum and the reaction crude was extracted with ethyl acetate. The organic phase was combined and washed with brine, water, and then dried with anhydrous Na_2SO_4. After removing the solvent by rotational evaporation, the residue was chromatographed on silica gel (chloroform/hexane = 1:2 to 3:1) to afford ***1*** as an orange-red solid (160 mg, 52% yield).

^{1}H NMR ($CDCl_3$, 300 MHz) δ 7.65-7.58 (m, 6H, Ar-H), 7.17 (dd, *J* = 8.0, 2.0 Hz, 2H, Ar-H), 6.88 (dd, *J* = 8.0, 2.0 Hz, 2H, Ar-H), 3.76 (t, *J* = 7.2, 4H, N-CH$_2$), 1.77-1.70 (m, 4H, -CH$_2$), 1.42-1.29 (m, 12H, -CH$_2$), 0.90 (t, *J* = 6.9, 6H, -CH$_3$).

^{13}C NMR ($CDCl_3$, 75 MHz) δ 182.3, 157.8, 151.9, 142.7, 137.7, 136.7, 128.9, 125.7, 113.87, 109.0, 40.3, 31.4, 27.2, 26.6, 22.5, 14.0.

HRMS (ESI) calculated for $C_{34}H_{37}N_2O_4^+$: 537.2748; Found:537.2747.

***2,2′-(1,4-phenylenebis(1-hexyl-2-oxoindoline-4-yl-3-ylidene)) dimalononitrile* (*2*)**:

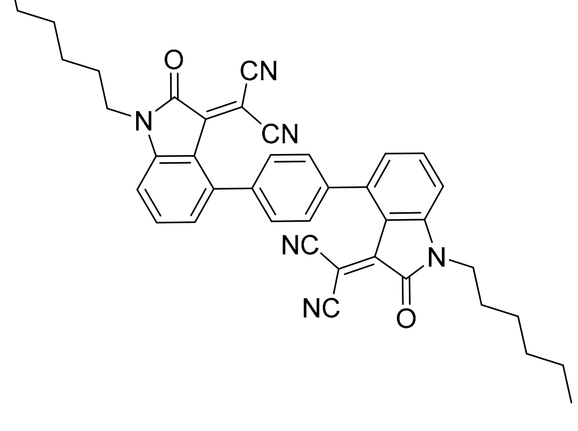

The compound ***1*** (60 mg, 0.11 mmol) and malononitrile (22 mg, 0.33 mmol, 3 eq) were dissolved in 3.5 ml of anhydrous EtOH. The resulting mixture was warmed to 80 °C and stirred for overnight. Then, the solvent was removed by rotational evaporation and ethyl acetate was added. The organic phase was washed 3 times with water, dried over anhydrous Na_2SO_4. After removing the solvent by rotational evaporation, the residue was chromatographed on silica gel (dichloromethane/hexane = 2:1) to afford **2** as a deep red solid (56 mg, 80% yield).

^{1}H NMR ($CDCl_3$, 300 MHz) δ 7.59-7.51 (m, 6H, Ar-H), 7.05 (d, *J* = 7.9 Hz, 2H, Ar-H), 6.82 (d, *J* = 8.0 Hz, 2H, Ar-H), 3.75 (t, *J* = 7.5 Hz, 4H, N-CH_2), 1.75-1.68 (m, 4H, -CH_2), 1.39-1.29 (m, 12H, -CH_2), 0.91 (t, *J* = 6.9, 6H, -CH_3).

^{13}C NMR ($CDCl_3$, 75 MHz) δ 163.1, 147.2, 144.0, 140.0, 136.9, 129.8, 126.5, 116.1, 112.0, 111.0, 108.5, 85.7, 40.7, 31.3, 27.2, 26.6, 22.5, 14.0.

HRMS (ESI) calculated for $C_{40}H_{37}N_6O_2^+$: 633.2976; Found: 633.2973.

***6,6′-(1,4-phenylene) bis(1-hexylindoline-2,3-dione) (3)*:**

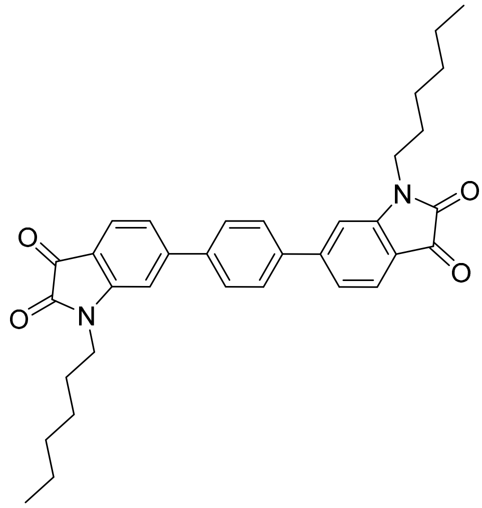

A mixture of ***17a*** (210 mg, 0.64 mmol), 1,4-Benzenediboronic acid bis (pinacol) ester (96 mg, 0.29 mmol, 0.45 eq), $P(o\text{-}tyl)_3$ (29 mg, 0.09 mmol, 14%) and K_3PO_4 (410 mg, 1.93 mmol, 3 eq) in a solution of toluene (5 mL) and water (0.5 mL), was loaded under argon. The reaction mixture was stirred for 0.5 h, $Pd_2(dba)_3$ (29 mg, 0.03 mmol, 4%) was then added and refluxed for overnight. The solvents were removed under vacuum distillation. Ethyl acetate was then added, the organic layer was separated and dried over Na_2SO_4. The crude product was chromatographically purified on silica gel eluting with (chloroform/hexane = 1:2 to 4:1) to provide ***3*** as a red solid (109 mg, 70% yield).

^{1}H NMR ($CDCl_3$, 300 MHz) δ 7.76 (s, 4H, Ar-H), 7.71 (d, *J* = 7.8 Hz, 2H, Ar-H), 7.36 (dd, *J* = 7.8, 1.4 Hz, 2H, Ar-H), 7.06 (s, 2H, Ar-H), 3.79 (t, *J* = 7.3 Hz, 4H, N-CH_2), 1.80-1.70 (m, 4H, -CH_2), 1.42-1.29 (m, 12H, -CH_2), 0.89 (t, *J* = 6.9 Hz, 6H, -CH_3).

^{13}C NMR ($CDCl_3$, 75 MHz) δ 182.9, 158.5, 151.7, 150.4, 140.4, 128.1, 126.0, 122.4, 116.7, 108.6, 40.3, 31.4, 27.4, 26.6, 22.5, 14.0.

HRMS (ESI) calculated for $C_{34}H_{37}N_2O_4^+$: 537.2748; Found:537.2748.

***2,2′-(1,4-phenylenebis(1-hexyl-2-oxoindoline-6-yl-3-ylidene)) dimalononitrile (4)*:**

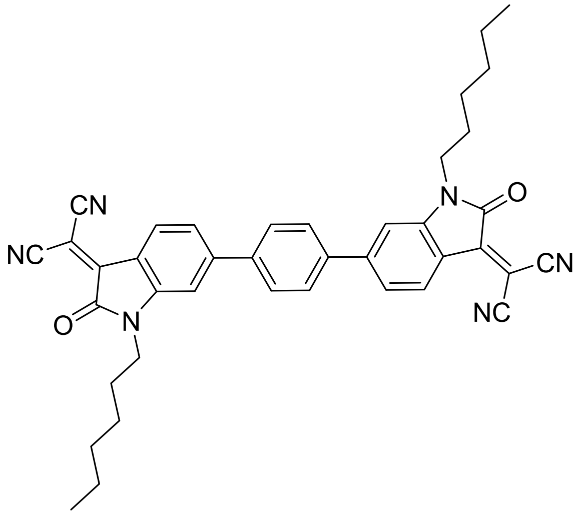

This compound was prepared from **3** by a similar synthetic procedure analogous to that of **2**. The crude was purified by chromatography on silica gel (dichloromethane/ petroleum ether = 2:1) to afford **4** as a red solid (50mg, 88% yield).

^{1}H NMR ($CDCl_3$, 300 MHz) δ 8.22 (dd, *J* = 8.2, 2.5 Hz, 2H, Ar-H), 7.78 (s, 4H, Ar-H), 7.39 (d, *J* = 8.2 Hz, 2H, Ar-H), 7.06 (s, 2H, Ar-H), 3.79 (t, *J* = 7.6 Hz, 4H, N-CH_2), 1.78-1.66 (m, 4H, -CH_2), 1.42-1.28 (m, 12H, -CH_2), 0.89 (t, *J* = 6.9 Hz, 6H, -CH_3).

^{13}C NMR ($CDCl_3$, 75 MHz) δ 162.7, 149.5, 148.6, 147.4, 140.3, 128.1, 127.4, 122.4, 117.7, 112.5, 108.0, 81.9, 40.6, 31.4, 27.4, 26.6, 22.5, 14.0.

HRMS (ESI) calculated for $C_{40}H_{37}N_6O_2^+$: 633.2976; Found: 633.2973.

***6,6′-(benzo[c] [1,2,5] thiadiazole-4,7-diyl) bis (1-hexylindoline-2,3-dione) (5)*:**

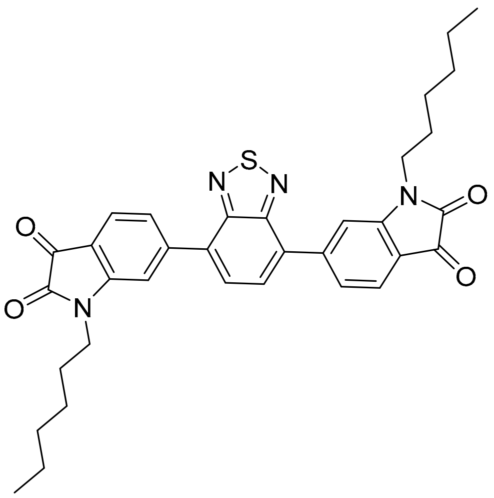

A mixture of compound ***17a*** (300 mg, 0.97 mmol), 2,1,3-Benzothiadiazole-4,7-diboronic acid bis(pinacol) ester (170 mg, 0.44 mmol, 0.45 eq), P(o-tyl)$_3$ (40 mg, 0.13 mmol, 14%), K_3PO_4 (617 mg, 2.91 mmol, 3 eq), toluene (2.5 mL) and water (0.5 ml) were loaded in a flask and purged with argon. After stirring in rt for 0.5 h, Pd$_2$(dba)$_3$ (40 mg, 0.04 mmol, 4%) was added and refluxed for overnight. Solvents were removed, the crude was extracted with ethyl acetate and dried over anhydrous Na_2SO_4. The crude was chromatographed on silica gel (chloroform/hexane = 3:2) to afford **5** as a red solid (145 mg, 55% yield).

^{1}H NMR ($CDCl_3$, 300 MHz) δ 7.95 (s, 2H, Ar-H), 7.78 (d, *J* = 7.9 Hz, 2H, Ar-H), 7.64 (d, *J* = 7.8 Hz, 2H, Ar-H), 7.62 (s, 2H, Ar-H), 3.82 (t, *J* = 7.3 Hz, 4H, N-CH_2), 1.84-1.74 (m, 4H, -CH_2), 1.44-1.28 (m, 12H, -CH_2), 0.89 (t, *J* = 7.1 Hz, 6H, -CH_3).

^{13}C NMR ($CDCl_3$, 75 MHz) δ 183.1, 158.3, 153.5, 151.2, 146.6, 133.3, 128.7, 125.6, 124.3, 117.2, 111.4, 40.4, 31.4, 27.3, 26.6, 22.5, 14.0.

HRMS (ESI) calculated for $C_{34}H_{35}N_4O_4S^+$:595.2373; Found: 595.2374.

***2,2'-(benzo[c][1,2,5]thiadiazole-4,7-diylbis(1-hexyl-2-oxoindoline-6-yl-3 ylidene))dimalononitrile (6)*:**

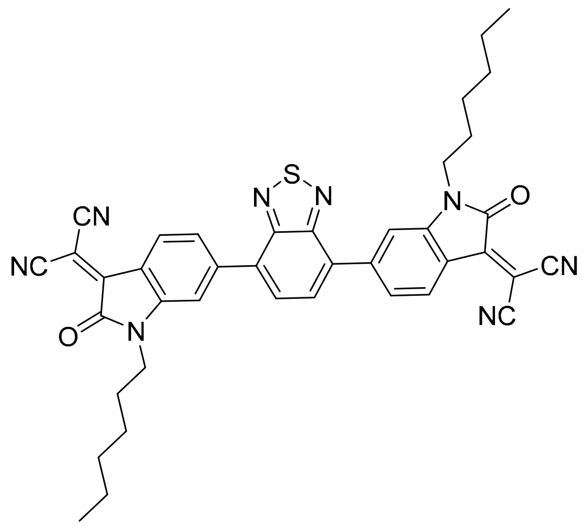

This compound was prepared from ***5*** by a similar synthetic procedure analogous to that of **2**. The crude was purified by chromatography on silica gel (dichloromethane/petroleum ether = 3:1) afforded ***6*** as a red solid (27 mg, 80% yield).

^{1}H NMR ($CDCl_3$, 300 MHz) δ 8.29 (d, *J* = 8.1 Hz, 2H, Ar-H), 7.98 (s, 2H, Ar-H),7.68 (d, *J* = 7.9 Hz, 2H, Ar-H), 7.65 (s, 2H, Ar-H), 3.81 (t, *J* = 7.3 Hz, 4H, N-CH_2), 1.82-1.68 (m, 4H, -CH_2), 1.44-1.29 (m, 12H, -CH_2), 0.89 (t, *J* = 7.2 Hz, 6H, -CH_3).

^{13}C NMR ($CDCl_3$, 75 MHz) δ 162.6, 153.5, 148.7, 146.9, 145.8, 133.1, 128.7, 127.1, 124.2, 118.3, 112.4, 110.9, 82.5, 40.7, 31.4, 27.3, 26.6, 22.5, 14.0.

HRMS (ESI) calculated for $C_{40}H_{35}N_8O_2S^+$:691.2600; Found: 691.2598.

6,6'-(thiophene-2,5-diyl) bis(1-hexylindoline-2,3-dione) (7a)**:**

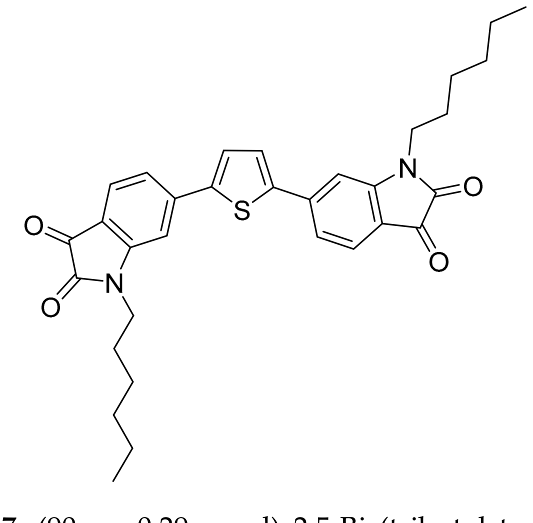

A mixture of compound ***17a*** (90 mg, 0.29 mmol), 2,5-Bis(tributylstannyl)thiophene (88 mg, 0.13 mmol, 0.45 eq), and anhydrous dimethylformamide (2 mL) was loaded in a flask and purged with argon. After stirred in rt for 0.5 h, Pd(dppf)Cl_2 (15 mg, 0.02 mmol, 6%) was added and refluxed overnight. DMF was removed under vacuum distillation, the residue was extracted

with ethyl acetate, dried over Na_2SO_4 and chromatographed on silica gel (dichloromethane /hexane = 1:1) to afford ***7a*** as a red solid (44 mg, 64% yield).

^{1}H NMR ($CDCl_3$, 300 MHz) δ 7.65 (d, *J* = 7.7 Hz, 2H, Ar-H), 7.55 (s, 2H, Ar-H), 7.39 (d, *J* = 7.9 Hz, 2H, Ar-H), 7.08 (s, 2H, Ar-H), 3.79 (t, *J* = 7.4 Hz, 4H, N-CH_2), 1.80-1.70 (m, 4H, -CH_2), 1.44-1.30 (m, 12H, -CH_2), 0.90 (t, *J* = 7.1 Hz, 6H, -CH_3).

^{13}C NMR ($CDCl_3$, 75 MHz) δ 182.3, 158.5, 151.8, 144.5, 142.9, 127.3, 126.3, 120.7, 116.8, 106.6, 40.3, 31.4, 27.3, 26.6, 22.5, 14.0.

HRMS (ESI) calculated for $C_{32}H_{35}N_2O_4S^+$:543.2310; Found: 543.2312.

2,2′-(thiophene-2,5-diylbis(1-hexyl-2-oxoindoline-6-yl-3-ylidene)) dimalononitrile (8a):

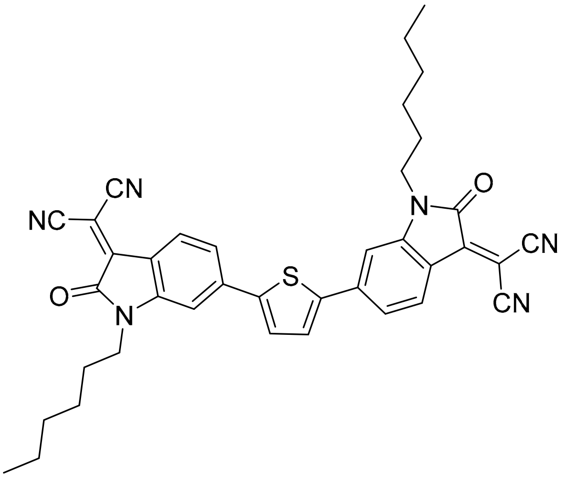

The compound ***7a*** (20 mg, 0.03 mmol) and malononitrile (8 mg, 0.11 mmol, 3 eq) were dissolved in 3.0 ml of anhydrous EtOH. Followed by the addition of 2 drops of pyridine by syringe. C. The mixture was then stirred overnight at room temperature. On completion, the solvent was removed by rotational evaporation and chloroform was added. The organic phase was washed 3 times with water, dried over anhydrous Na_2SO_4. The crude was purified by chromatography on silica gel (dichloromethane/petroleum ether = 1:2) to afford ***8a*** as a deep-red solid (17 mg, 89% yield).

^{1}H NMR ($CDCl_3$, 300 MHz) δ 8.15 (d, *J* = 8.5 Hz, 2H, Ar-H), 7.59 (s, 2H, Ar-H),7.40 (d, *J* = 8.4 Hz, 2H, Ar-H), 7.05 (s, 2H, Ar-H), 3.77 (t, *J* = 6.9 Hz, 4H, N-CH_2), 1.78-1.68 (m, 4H, -CH_2), 1.42-1.30 (m, 12H, -CH_2), 0.90 (t, *J* = 7.1 Hz, 6H, -CH_3).

^{13}C NMR ($CDCl_3$, 75 MHz) δ 162.8, 147.9, 147.4, 144.9, 142.0, 127.6, 120.6, 117.9, 112.6, 110.8, 106.0, 81.4, 40.6, 31.3, 27.4, 26.5, 22.5, 14.0.

HRMS (ESI) calculated for $C_{38}H_{35}N_6O_2S^+$:639.2542; Found: 639.2537.

6,6′-([2,2′:5′,2′′-terthiophene]-5,5′′-diyl) bis(1-hexylindoline-2,3-dione) (9a):

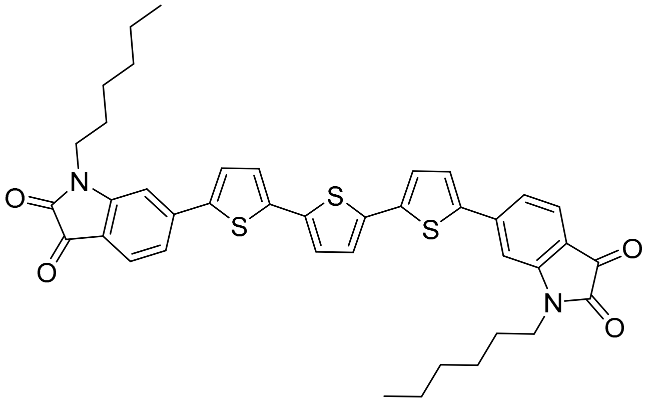

This compound was prepared from ***19a*** (150 mg, 0.38 mmol), 2,5-Bis(tributylstannyl)thiophene (105 mg, 0.15 mmol, 0.40 eq), DMF (5 mL) and Pd(dppf)Cl_2 (15 mg, 0.02 mmol, 5%) as described for **7a**. The crude was purified on silica gel (dichloromethane/hexane = 1:1 to 2:1) to afford ***9a*** as a red solid (83 mg, 75% yield).

^{1}H NMR ($CDCl_3$, 300 MHz) δ 7.62 (dd, *J* = 8.2, 3.7 Hz, 2H, Ar-H), 7.45 (d, *J* = 3.7 Hz, 2H, Ar-H), 7.32 (dd, *J* = 7.5, 2.9 Hz, 2H, Ar-H), 7.24 (d, *J* = 3.1 Hz, 2H, Ar-H), 7.22 (s, 2H, Ar-H), 7.03 (s, 2H, Ar-H), 3.78 (t, *J* = 7.6 Hz, 4H, N-CH_2), 3.78-3.72 (m, 4H, -CH_2), 1.42-1.32 (m, 12H, -CH_2), 0.95-0.84 (m, 6H, -CH_3).

^{13}C NMR ($CDCl_3$, 75 MHz) δ 182.2, 158.7, 151.8, 143.3, 141.4, 139.3, 136.4, 127.1, 126.2, 125.5, 125.3, 120.2, 116.3, 106.2, 40.2, 31.4, 27.4, 26.6, 22.5, 14.0.

HRMS (ESI) calculated for $C_{40}H_{39}N_2O_4S_3^+$:707.2059; Found: 707.2066.

6,6′-([2,2′:5′,2′′-terthiophene]-5,5′′-diyl)bis(1-(2-octyldodecyl)indoline-2,3-dione) (9b):

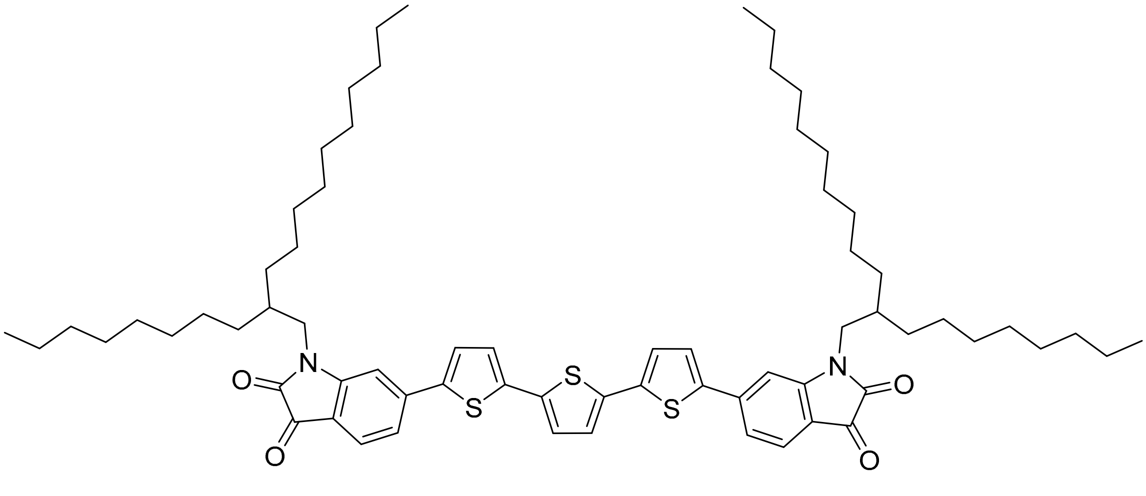

This compound was prepared from ***19b*** (42 mg, 0.07 mmol), 2,5-Bis(tributylstannyl) thiophene (20 mg, 0.03 mmol, 0.42 eq), DMF (2.5 mL) and Pd(dppf)Cl_2 (8 mg, 0.01 mmol, 14%) as described for **7a**. The crude was purified on silica gel (ethyl acetate/hexane = 1:3 to 4:1) to afford ***9b*** as a red solid (15 mg, 50% yield).

^{1}H NMR (CDCl$_3$, 300 MHz) δ 7.62 (dd, *J* = 8.1, 1.4 Hz, 2H, Ar-H), 7.43 (d, *J* = 3.3 Hz, 2H, Ar-H), 7.33 (d, *J* = 8.5 Hz, 2H, Ar-H), 7.24 (d, *J* = 3.2 Hz, 2H, Ar-H), 7.21 (s, 2H, Ar-H), 7.04 (s, 2H, Ar-H), 3.66 (d, *J* = 7.1 Hz, 4H, N-CH$_2$), 1.94-1.82 (m, 2H, N-CH$_2$-CH$_1$), 1.38-1.22 (m, 64H, -CH$_2$), 0.92-0.86 (m, 12H, -CH$_3$).

^{13}C NMR (CDCl$_3$, 75 MHz) δ 182.2, 152.2, 141.4, 139.3, 136.4, 127, 126.2, 125.5, 125.3, 120.1, 116.3, 106.5, 44.6, 36.2, 31.9, 31.9, 31.6, 30.0, 29.7, 29.4, 29.4, 28.3, 26.6, 22.7, 14.2, 13.6.

HRMS (ESI) calculated for $C_{68}H_{95}N_2O_4S_3^+$:1099.6450; Found: 1099.6448.

2,2′-([2,2′:5′,2′′-terthiophene]-5,5′′-diylbis(1-(2-octyldodecyl)-2-oxoindoline-6-yl-3-ylidene)) dimalononitrile (10b):

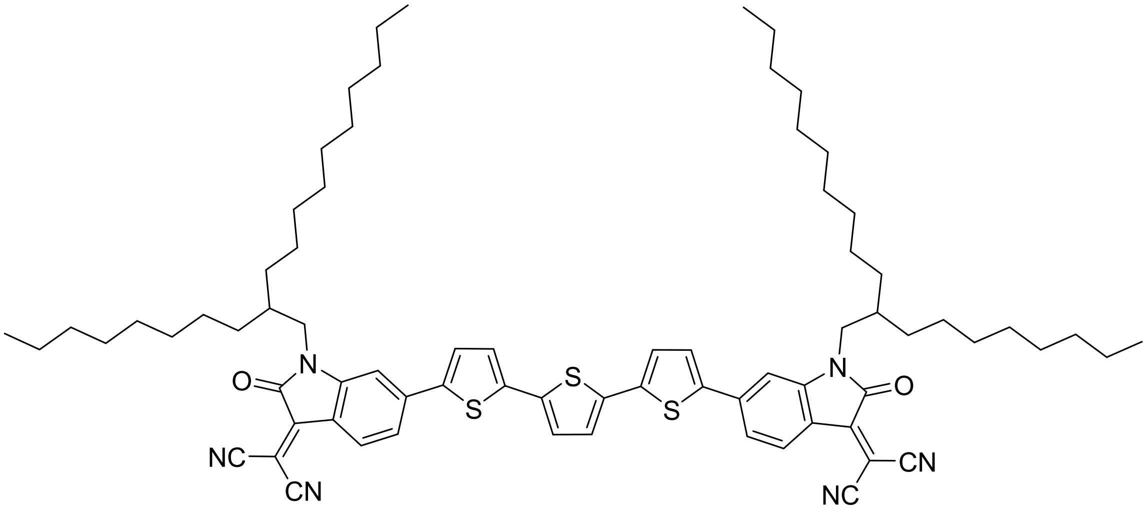

This compound was prepared from ***9b*** (10 mg) and malononitrile (2 mg) in 1 ml of dry EtOH and 1 ml of DCM as described for **2**. The crude was purified by chromatography on silica gel (dichloromethane/petroleum ether = 1:1) to afford ***10b*** as a deep blue solid (10mg, 95% yield).

^{1}H NMR (CDCl$_3$, 300 MHz) δ 8.11 (d, *J* = 8.3 Hz, 2H, Ar-H), 7.47 (d, *J* = 4.0 Hz, 2H, Ar-H), 7.34 (d, *J* = 7.9 Hz, 2H, Ar-H), 7.25 (d, *J* = 3.1 Hz, 2H, Ar-H), 7.22 (s, 2H, Ar-H), 6.98 (s, 2H, Ar-H), 3.63 (d, *J* = 7.1 Hz, 4H, N-CH$_2$), 1.91-1.81 (m, 2H, N-CH$_2$-CH$_1$), 1.43-1.18 (m, 64H, -CH$_2$), 0.91-0.87 (m, 12H, -CH$_3$).

^{13}C NMR (CDCl$_3$, 75 MHz) δ 163.3, 147.9, 147.7, 142.4, 141.5, 139.8, 136.5, 127.5, 125.7, 125.5, 120.0, 117.1, 112.9, 111.1, 105.8, 80.3, 44.9, 36.4, 31.9, 31.9, 31.6, 30.0, 29.7, 29.6, 29.4, 29.4, 26.6, 22.7, 14.2.

HRMS (APPI) calculated for $C_{74}H_{95}N_2O_6S_3^+$: 1195.6673; Found: 1195.6653.

6,6′-(thiophene-2,5-diyl) bis(1-hexyl-5-nitroindoline-2,3-dione) (7b):

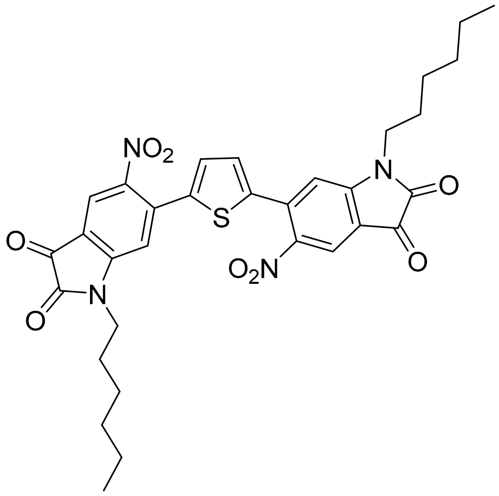

This compound was prepared from ***17b*** (210 mg, 0.59 mmol), 2,5-Bis(tributylstannyl)thiophene (163 mg, 0.25 mmol, 0.49 eq), DMF 2 mL and Pd(dppf)Cl_2 (21 mg, 0.03 mmol, 5%) as described for ***7a***. The crude was purified on silica gel (dichloromethane/hexane = 2:1) to afford ***7b*** as a red solid (140 mg, 90% yield).

^{1}H NMR ($CDCl_3$, 300 MHz) δ 8.16 (s, 2H, Ar-H), 7.22 (s, 2H, Ar-H), 7.04 (s, 2H, Ar-H), 3.82 (t, *J* = 7.3 Hz, 4H, N-CH_2), 1.76-1.69 (m, 4H, -CH_2), 1.40-1.34 (m, 12H, -CH_2), 0.91-0.86 (m, 6H, -CH_3).

^{13}C NMR ($CDCl_3$, 75 MHz) δ 180.8, 157.7, 152.8, 144.6, 139.8, 137.8, 129.4, 122.1, 116.4, 113.1, 40.9, 31.3, 27.3, 26.5, 22.5, 14.0.

HRMS (ESI) calculated for $C_{32}H_{33}N_4O_8S^+$:633.2013; Found: 633.2014.

2,2'-(thiophene-2,5-diylbis(1-hexyl-5-nitro-2-oxoindoline-6-yl-3-ylidene))dimalononitrile (8b):

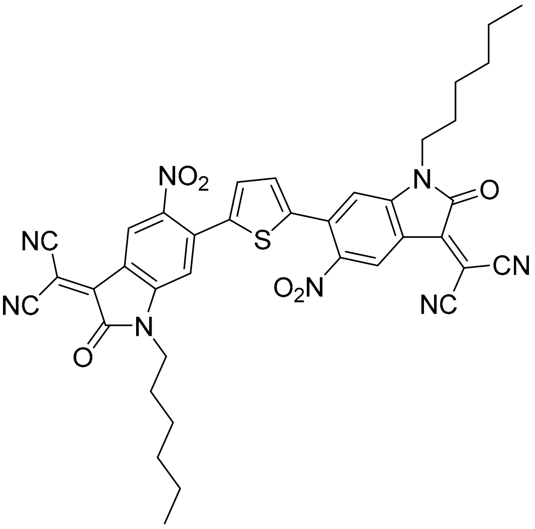

This compound was prepared from ***7b*** (30 mg, 0.04 mmol), malononitrile (10 mg, 0.14 mmol, 3.5 eq) and 3 ml of anhydrous EtOH as described for **2**. The crude was purified on silica gel (dichloromethane/ petroleum ether = 1:1) to afford ***8b*** as a deep red solid (29 mg, 85% yield).

^{1}H NMR ($CDCl_3$, 300 MHz) δ 8.62 (s, 2H, Ar-H), 7.24 (s, 2H, Ar-H), 7.00 (s, 2H, Ar-H), 3.82 (t, *J* = 7.4 Hz, 4H, N-CH_2), 1.74-1.69 (m, 4H, -CH_2), 1.40-1.34 (m, 12H, -CH_2), 0.92-0.86 (m, 6H, -CH_3).

^{13}C NMR ($CDCl_3$, 75 MHz) δ162.2, 148.4, 146.5, 144.4, 140.0, 136.6, 129.8, 123.0, 117.6, 112.2, 111.5, 109.8, 85.7, 41.2, 31.2, 27.3, 26.5, 22.46, 14.0.

HRMS (ESI) calculated for $C_{38}H_{33}N_8O_6S^+$: 729.2245; Found: 729.2238.

3. *UV-vis absorption spectra*

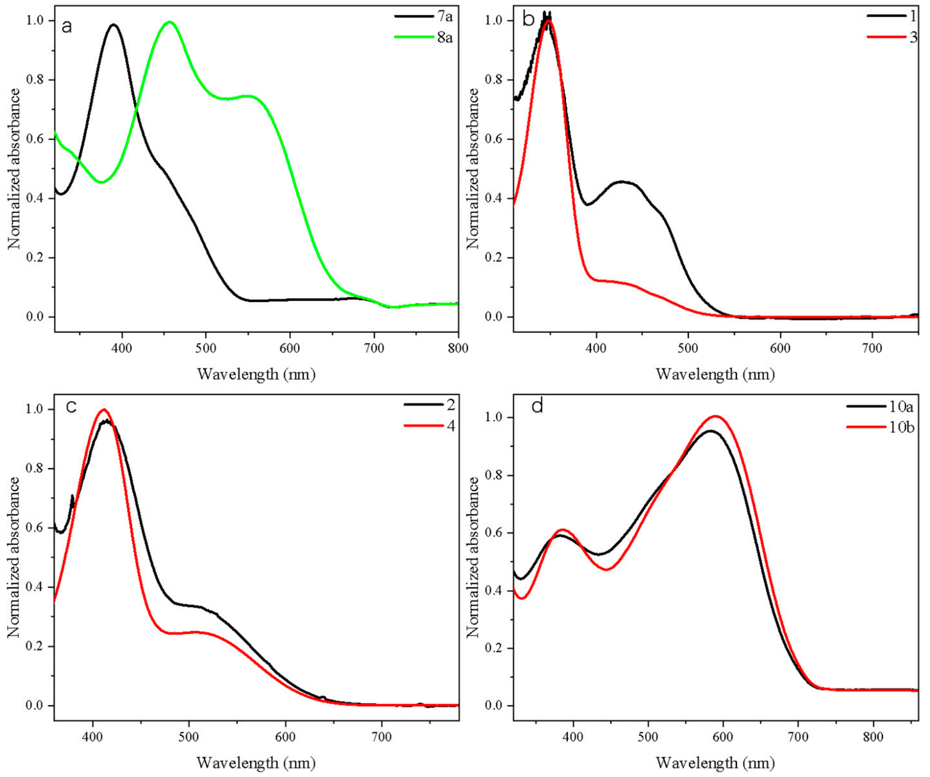

Figure S1. Normalized UV-vis absorption spectra of (**a**) Compound **7a** and **8a** in THF; (**b**) bis-isatin compounds **1** and **3** in THF; (**c**) di-2-(2-oxindolin-3-ylidene) malononitrile compounds **2** and **4** in THF; (**d**) di-2-(2-oxindolin-3-ylidene) malononitrile compounds **10a** and **10b** in THF.

The introduction of dicyano groups (malononitrile compounds) leads to a red shift of the maximum absorption peak by about 100 nm as shown in Fig. a. Based on Figs. b and c for the materials based on benzene ring as a bridging group, the position of the substituent group does not have a significant effect on the position of the absorption peaks but mainly affects the intensity of their ICT peaks at the same molecular composition. As shown in Fig. d, changing the substituent at the N position with different alkyl chain lengths has almost no effect on the absorption spectrum.

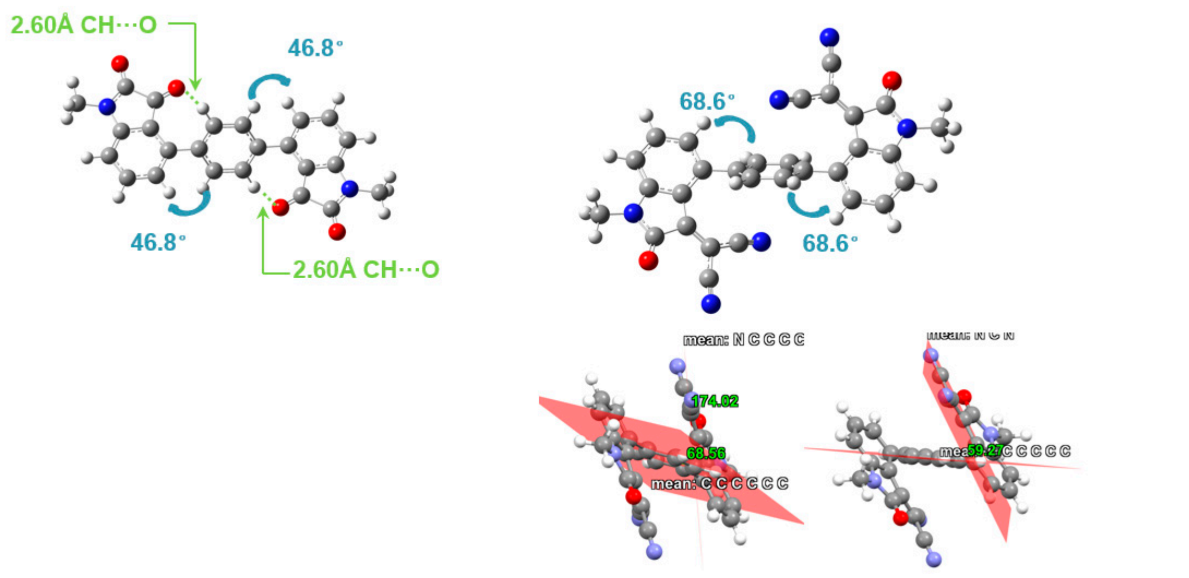

Figure S2. The lowest energy configuration of compound 1 and 2 obtained by relying on DFT at the B3LYP-D3/def2tzvp level.

Based on the results of the theoretical simulations, it can be seen that the planarity of material 1 is better. While the molecular co-planarity of material 2 is relatively poor, which is related to the space site resistance with the benzene ring caused by the length of the cyano group.

5. Figure S3. HOMO/LUMO maps of bis-isatin and di-2-(2-oxindolin-3-ylidene) malononitrile derivatives.

Acronym	HOMO map	LUMO map
**1**	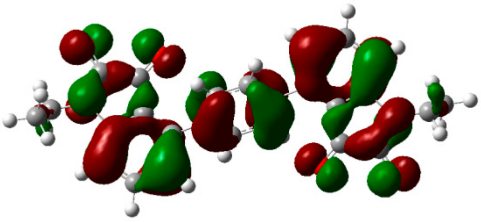	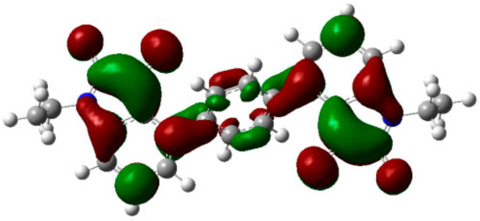
**2**	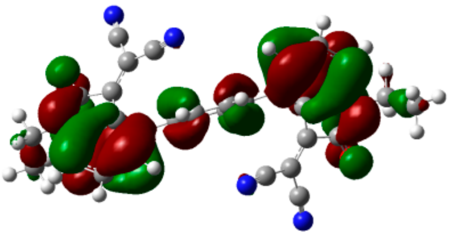	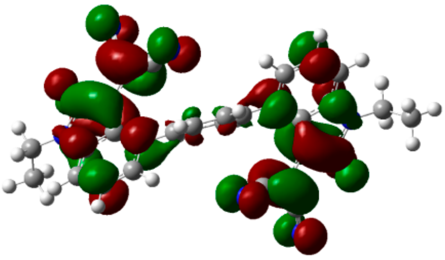
**3**	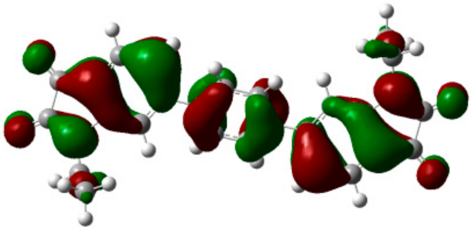	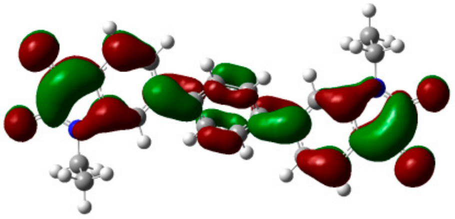
**4**	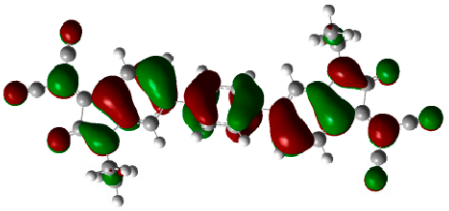	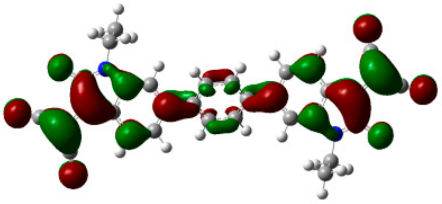
**5**	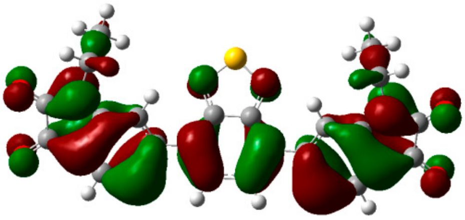	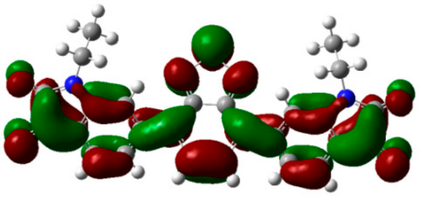
**6**	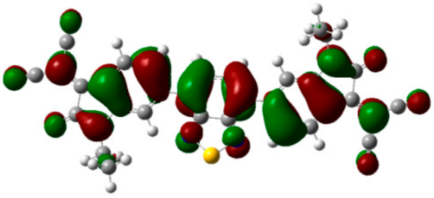	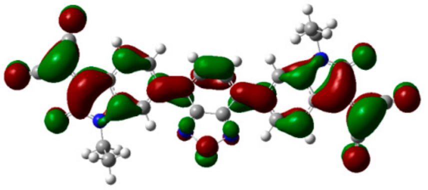
**7a**	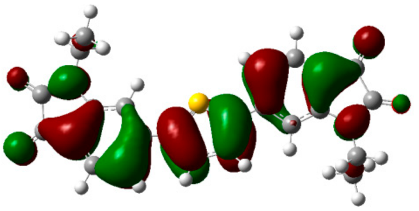	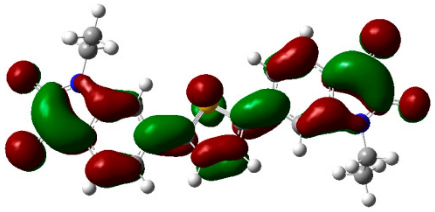

8a

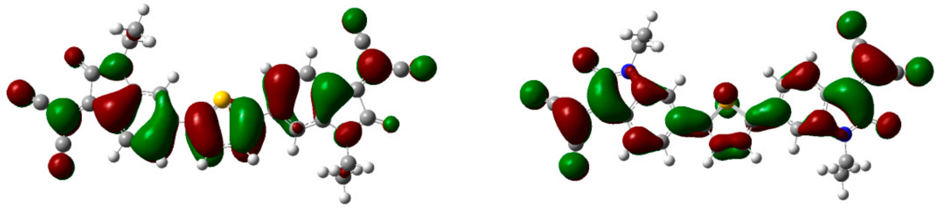

8b

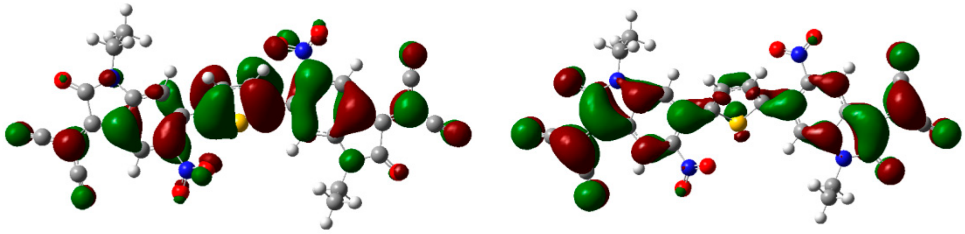

9

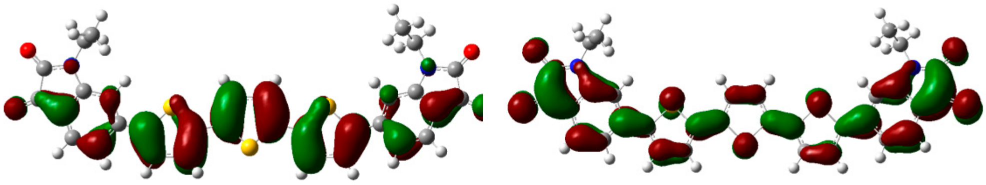

10

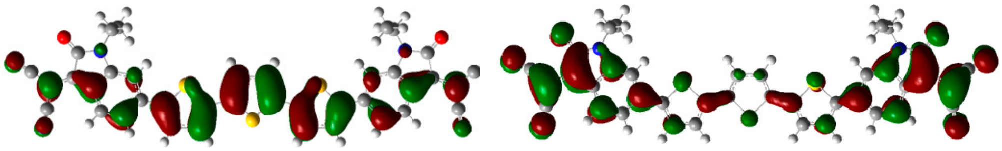

6.Device fabrication

6.1. Perovskite solar cells fabrication

The inverted planer heterojunction structure solar cells were fabricated under N_2 atmosphere conditions and started from ITO/PEDOT:PSS. The $(MA_{0.17}FA_{0.83})_{0.95}Cs_{0.05}Pb(I_{0.83}Br_{0.17})_3$ perovskite precursor solution was spin-coated onto the substrate via a two-step speed process at 2000 rpm for 10 s and 4000 rpm for 30 s, respectively. During the second spin-coating step, 100 µL of chlorobenzene (CB) was dropped onto the substrate. The resulting film was then annealed at 100 °C for 30 min for enhanced crystallization. Afterwards, PCBM as reference or **10a** as electron transport layers were spin-coated on perovskite layer, without further annealing. Finally, BCP (5 nm), used as hole blocking layer, was evaporated and deposited onto the ETL and, a thick contact layer of Ag (100 nm) was used as top electrode by using this thermal evaporator condition under vacuum (6×10^{-6} mbar) through a shadow mask to define the active area (0.28 cm^2) of the devices.

6.2. Preparation of ITO substrates

Pre-patterned indium-tin oxide (ITO) coated glass slides of 1.5 x 2 cm^2 with a sheet resistance of Rs = 7 $\Omega.sq^{-1}$ were provided at our own lab (LPICM) from Sputtering - Alliance concept Systems. To remove the contaminants from the ITO surface, were cleaned in ultrasonic baths for 15 minutes for each step in the following order: deionized water with detergent (2 drops of Deconex® 12 PA-x solution), then acetone and finally isopropanol and were dried with nitrogen gun. Eventually, the UV-ozone treatment was performed for 15 minutes ensuring elimination of residual organic contaminants of the ITO substrates.

6.3. Deposition of PEDOT:PSS

A filtered aqueous solution of poly(3,4-ethylenedioxy-thiophene):poly(styrenesulfonate) (purchased from Ossila, Al 4083) through the PVDF (hydrophilic / 45 µm) filter is dispensed on the ITO substrate at room temperature and then spun-cast at 4500 rpm for 40 s. Then, the edge parts of the conductive polymer film are removed by cotton swabs before being baked. The last step, the PEDOT:PSS deposit samples are annealed at 120°C for 30 minutes outside the glovebox. After the PEDOT:PSS films are introduced in the glovebox to avoid any reabsorption of water from the atmosphere.

6.4. Preparation of Triple Cation Perovskite Precursor Solution

The triple cation perovskite solution $(MA_{0.17}FA_{0.83})_{0.95}Cs_{0.05}Pb(I_{0.83}Br_{0.17})_3$ was prepared by mixing a precursor solution of formamidinium iodide (1 M), methylammonium bromide (0.2 M), lead iodide (1.1 M) and lead bromide (0.2) in a mixture of DMSO/DMF (1:4) solvent system. Then by adding 1.5 molar ratio of CsI (dissolved in DMSO) solution. The perovskite precursor solution was stirred at room temperature under a N_2 atmosphere between 4 to 5 h before use.

6.5. Preparation of the PCBM (Reference ETL)

The PCBM solution was made by dissolving 20 mg of PCBM in 1 mL of anhydrous CB and stirred for 4 or 5 h at 70°C. The solution was spin-coated onto the perovskite film and spin-coated 6000 rpm for 30 s.

6.6. Preparation of the 10a solution (ETL)

Have prepared by adding the 5 mg of **10a** in 2 mL of CB and stirred for 2 h at 60°C. The solution was spin-coated onto the perovskite film at 1000 rpm for 30 s.

6.7. Preparation of the 4 solution (passivating agent)

The **4** solution was made by dissolving 3 mg in 1 mL of CB and stirred for 2 h at 70°C. The solution was spin-coated as an antisolvent /or passive treatment onto the perovskite films, eg., following this condition: perovskite precursor solution was spin-coated onto the substrate via a two-step speed process at 2000 rpm for 10 s and 4000 rpm for 30 s, respectively. During the second spin-coating step, 100 μL of **4** solution was dropped onto the substrate. The resulting film was then annealed at 100 °C for 30 min for enhanced crystallization.

6.8. Space charge limited current (SCLC) device fabrication

To evaluate the electron mobility of **10a**, we fabricated electron-only devices with the structures of ITO/SnO_2/**10a**/LiF/Al and performed SCLC measurements. According to the charge behaviour, the *J-V* log-log plots can be divided into three regions: ohmic region, trap-filled limited (TFL) region, SCLC region.

6.9. Preparation of SnO_2 nanoparticle solution by sol-gel method

The precursor solution of SnO_2 (0.1 M) was obtained by dissolving 112.8 mg precursor of tin ($SnCl_2{\cdot}2H_2O$) in 5 mL absolute ethanol and then SnO_2 solution stirred 6h at 80 °C. After the solution kept at room temperature for at least 1 day, allowing the aging process. Afterward, we used this solution for preparing SnO_2 films by spin-coating without any purification at a speed rate of 3000 rpm for 40 s and have thermal annealing at 180 °C for 1 h on hot plate.

6.10. Preparation of the 10a solution (SCLC fabrication)

The **10a** solution was made by dissolving 5 mg in 1 mL of CB and stirred for 4 or 5 h at 70°C. The solution was spin-coated onto the SnO_2 films and spin-coated 2000 rpm for 30 s and the thickness determined with profilometer around 88 nm.

6.11. Deposition of LiF and Al

LiF can be used as cathode interlayer to favor electron extraction. LiF and Al layers were deposited by vacuum evaporation to reach thicknesses of 7 and 100 nm, respectively (under a pressure of 10^{-7} mbar at an evaporation rate of ca. 0.4 Å/s).

7. **Figure S4.** AFM image of PSCs device (**a**) and SCLC device for measuring the electron mobility (**b**).

a)

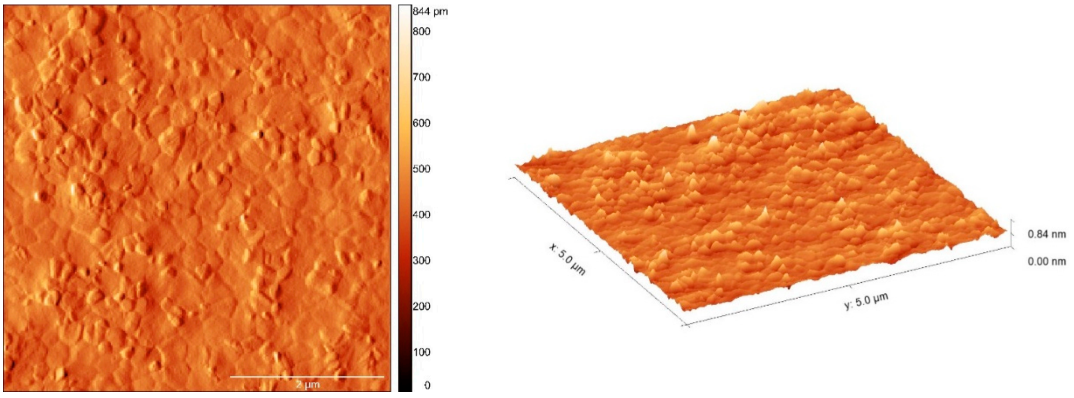

ITO/PEDOT PSS/3CP/10a/BCP

b)

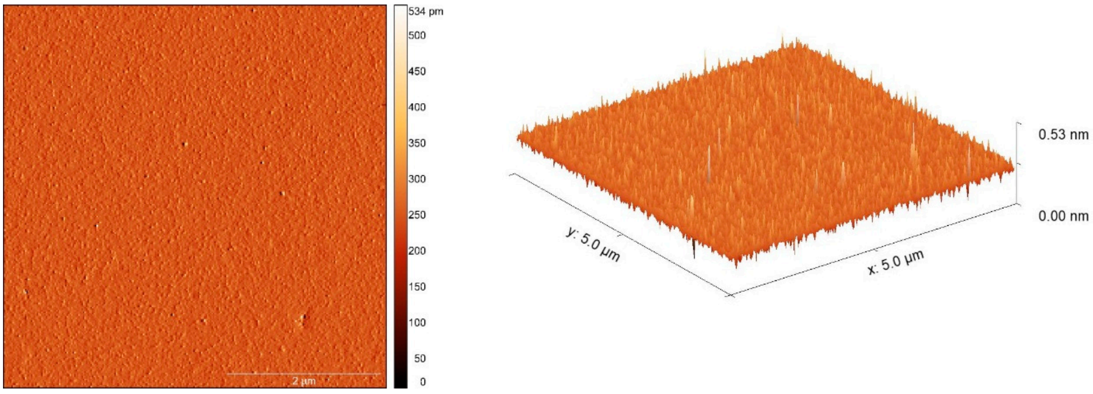

ITO/SnO_2/10a/LiF

8. Mass spectra

Figure S5. Mass spectra of Compound **1**.

Fédération de Recherche Physique et Chimie du Vivant (FR2708 : CBM/ICOA)
Plate-forme de Spectrométrie de Masse Haute Résolution

HRAM

Analysis Info

Sample Name	**RSW168**	Acquisition Date	12/02/2020 13:46:55
Analysis Name	X054829CYC.d	Instrument / Ser#	maXis 255552.00086
		Method	Positif.m

Acquisition Parameter

Source Type	ESI	Ion Polarity	Positive	Set Nebulizer	0.6 Bar
Scan Begin	50 m/z	Set Capillary	4500 V	Set Dry Heater	200 °C
Scan End	3000 m/z	Set Collision Cell RF	1800.0 Vpp	Set Dry Gas	7.0 l/min

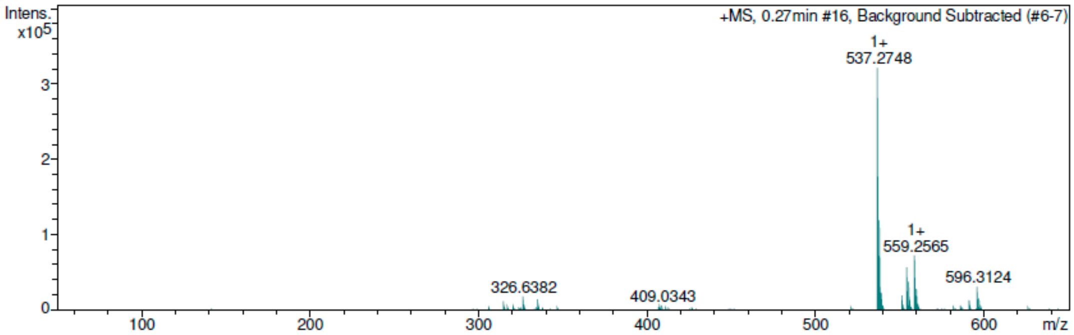

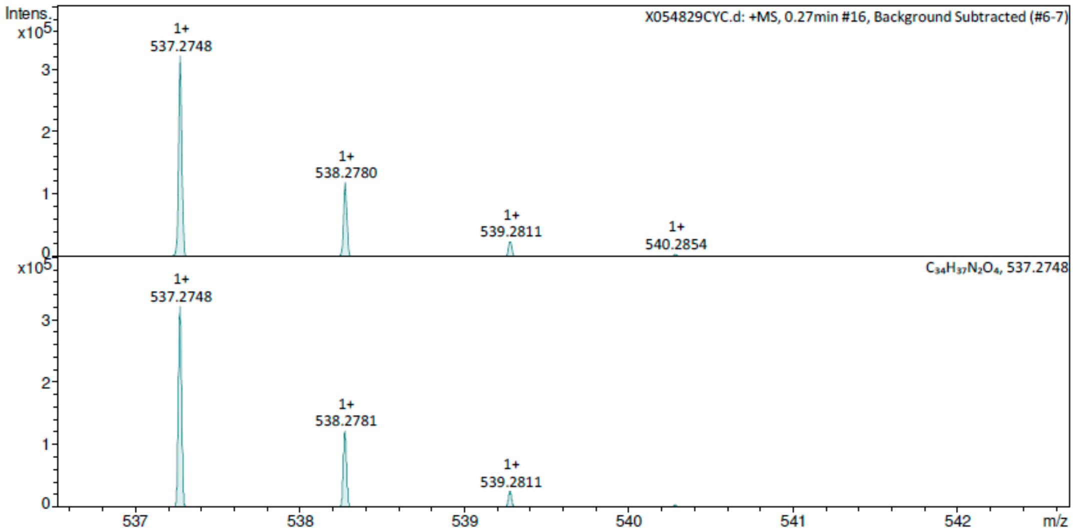

Meas. m/z	z	#	Ion Formula	m/z	err [ppm]	mSigma	rdb	e⁻ Conf
537.274808	1+	1	C34H37N2O4	537.274784	-0.0	5.3	18.0	even
554.301588	1+	1	C34H40N3O4	554.301333	-0.5	n.a.	17.0	even
559.256542	1+	1	C34H36N2NaO4	559.256728	0.3	10.3	18.0	even

Institut de Chimie Organique et Analytique
UMR 7311 - Université d'Orléans - CNRS
BP 6759 - rue de Chartres - F-45067 ORLEANS Cedex 2
cyril.colas@univ-orleans.fr
+33 (0)2 38 49 46 61
printed: 17/02/2020 18:25:03
Page 1 of 1

Figure S6. Mass spectra of Compound **2.**

Fédération de Recherche Physique et Chimie du Vivant (FR2708 : CBM/ICOA)
Plate-forme de Spectrométrie de Masse Haute Résolution

HRAM

**Analysis Info**		Acquisition Date	12/02/2020 13:48:25
Sample Name	**RSW170**	Instrument / Ser#	maXis 255552.00086
Analysis Name	X054830CYC.d	Method	Positif.m

Acquisition Parameter

Source Type	ESI	Ion Polarity	Positive	Set Nebulizer	0.6 Bar
Scan Begin	50 m/z	Set Capillary	4500 V	Set Dry Heater	200 °C
Scan End	3000 m/z	Set Collision Cell RF	1800.0 Vpp	Set Dry Gas	7.0 l/min

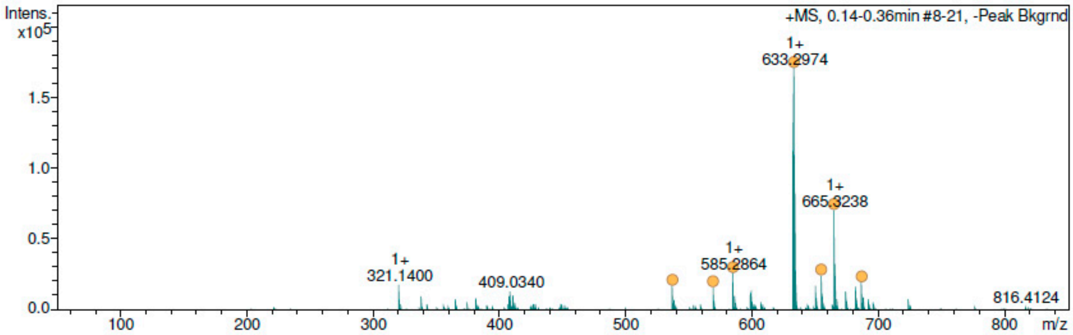

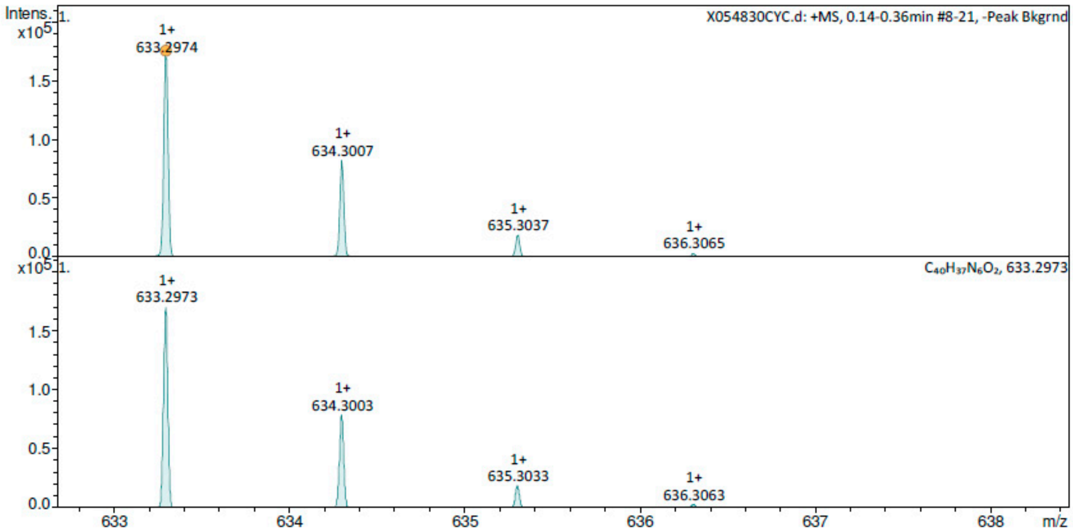

Meas. m/z	z	#	Ion Formula	m/z	err [ppm]	mSigma	rdb	e⁻ Conf
537.275203	1+	1	C35H33N6	537.276122	1.7	15.3	23.0	even
	1+	2	C34H37N2O4	537.274784	-0.8	18.6	18.0	even
569.291132	1+	1	C37H37N4O2	569.291103	-0.1	12.5	22.0	even
585.286414	1+	1	C37H37N4O3	585.286017	-0.7	62.5	22.0	even
633.297415	1+	1	C40H37N6O2	633.297251	-0.3	11.9	26.0	even
655.279573	1+	1	C40H36N6NaO2	655.279195	-0.6	7.6	26.0	even
665.323765	1+	1	C41H41N6O3	665.323466	-0.5	5.8	25.0	even
687.305492	1+	1	C41H40N6NaO3	687.305410	-0.1	7.1	25.0	even

Institut de Chimie Organique et Analytique
UMR 7311 - Université d'Orléans - CNRS
BP 6759 - rue de Chartres - F-45067 ORLEANS Cedex 2
cyril.colas@univ-orleans.fr
+33 (0)2 38 49 46 61

printed: 17/02/2020 18:28:07
Page 1 of 2

Figure S7. Mass spectra of Compound **3.**

Fédération de Recherche Physique et Chimie du Vivant (FR2708 : CBM/ICOA)
Plate-forme de Spectrométrie de Masse Haute Résolution

HRAM

Analysis Info

Sample Name	**RSW188**	Acquisition Date	12/02/2020 13:52:54
Analysis Name	X054833CYC.d	Instrument / Ser#	maXis 255552.00086
		Method	Positif.m

Acquisition Parameter

Source Type	ESI	Ion Polarity	Positive	Set Nebulizer	0.6 Bar
Scan Begin	50 m/z	Set Capillary	4500 V	Set Dry Heater	200 °C
Scan End	3000 m/z	Set Collision Cell RF	1800.0 Vpp	Set Dry Gas	7.0 l/min

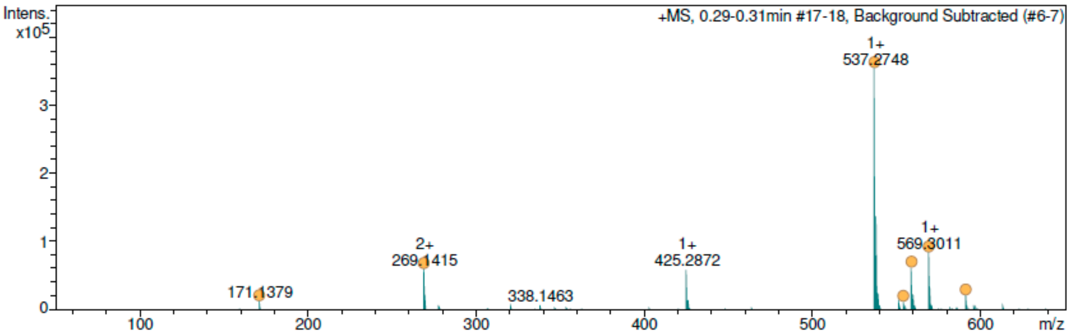

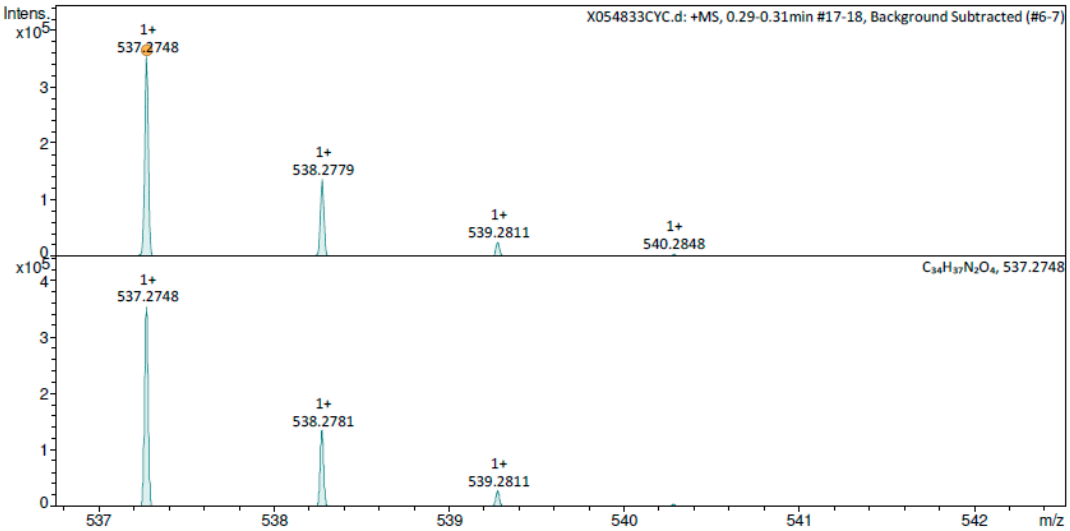

Meas. m/z	z	#	Ion Formula	m/z	err [ppm]	mSigma	rdb	e⁻ Conf
171.137918	1+	1	C10H19O2	171.137956	0.2	6.1	2.0	even
269.141536	2+	1	C34H38N2O4	269.141030	-1.9	8.9	18.0	even
537.274823	1+	1	C34H37N2O4	537.274784	-0.1	4.6	18.0	even
554.301853	1+	1	C34H40N3O4	554.301333	-0.9	42.1	17.0	even
559.256679	1+	1	C34H36N2NaO4	559.256728	0.1	5.1	18.0	even
569.301075	1+	1	C35H41N2O5	569.300999	-0.1	12.5	17.0	even
591.282989	1+	1	C35H40N2NaO5	591.282943	-0.1	22.9	17.0	even

Institut de Chimie Organique et Analytique
UMR 7311 - Université d'Orléans - CNRS
BP 6759 - rue de Chartres - F-45067 ORLEANS Cedex 2

cyril.colas@univ-orleans.fr
+33 (0)2 38 49 46 61

printed: 17/02/2020 18:39:14
Page 1 of 1

Figure S8. Mass spectra of Compound **4.**

Fédération de Recherche Physique et Chimie du Vivant (FR2708 : CBM/ICOA)
Plate-forme de Spectrométrie de Masse Haute Résolution

HRAM

Analysis Info

Sample Name	**RSW191**	Acquisition Date	12/02/2020 13:54:27
Analysis Name	X054834CYC.d	Instrument / Ser#	maXis 255552.00086
		Method	Positif.m

Acquisition Parameter

Source Type	ESI	Ion Polarity	Positive	Set Nebulizer	0.6 Bar
Scan Begin	50 m/z	Set Capillary	4500 V	Set Dry Heater	200 °C
Scan End	3000 m/z	Set Collision Cell RF	1800.0 Vpp	Set Dry Gas	7.0 l/min

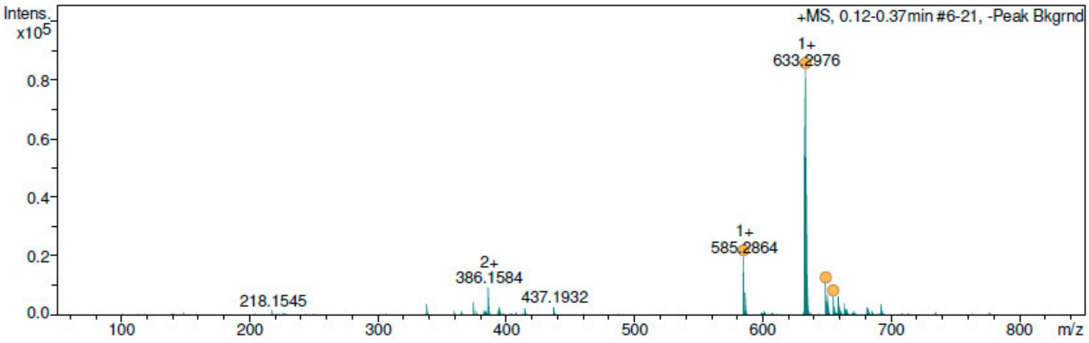

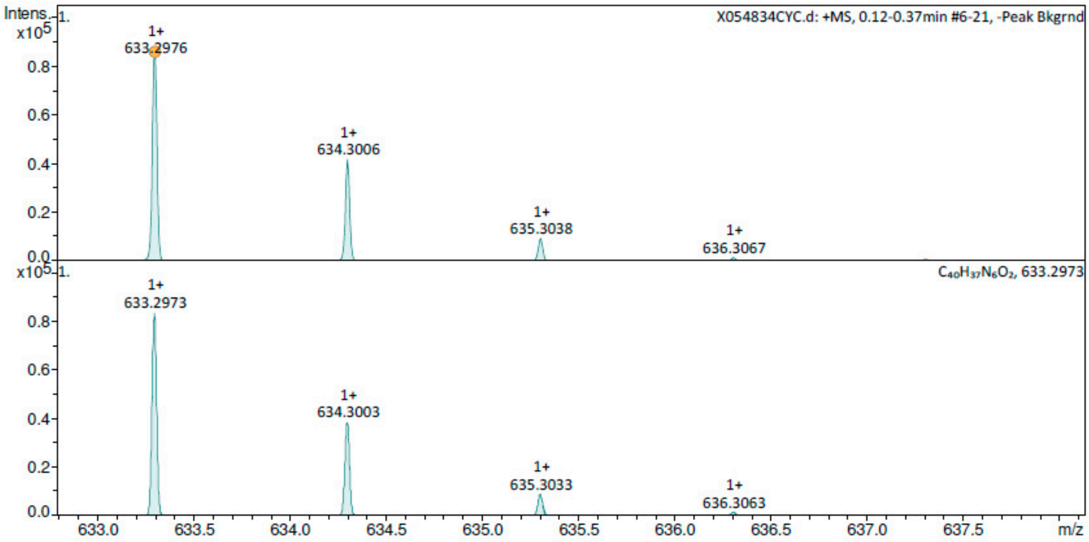

Meas. m/z	z	#	Ion Formula	m/z	err [ppm]	mSigma	rdb	e⁻ Conf
585.286429	1+	1	C36H41O7	585.284680	-3.0	3.3	17.0	even
	1+	2	C37H37N4O3	585.286017	-0.7	14.8	22.0	even
633.297606	1+	1	C40H37N6O2	633.297251	-0.6	19.7	26.0	even
649.292350	1+	1	C40H37N6O3	649.292165	-0.3	n.a.	26.0	even
	1+	2	C27H45N4O14	649.292679	0.5	n.a.	8.0	even
655.280936	1+	1	C40H36N6NaO2	655.279195	-2.7	25.8	26.0	even
	1+	2	C44H40NaO4	655.281880	1.4	26.8	25.0	even

Institut de Chimie Organique et Analytique
UMR 7311 - Université d'Orléans - CNRS
BP 6759 - rue de Chartres - F-45067 ORLEANS Cedex 2
cyril.colas@univ-orleans.fr
+33 (0)2 38 49 46 61

printed: 17/02/2020 18:41:18

Page 1 of 1

Figure S9. Mass spectra of Compound **5.**

Fédération de Recherche Physique et Chimie du Vivant (FR2708 : CBM/ICOA)
Plate-forme de Spectrométrie de Masse Haute Résolution

HRAM

**Analysis Info**		Acquisition Date	12/02/2020 13:49:56
Sample Name	**RSW183**	Instrument / Ser#	maXis 255552.00086
Analysis Name	X054831CYC.d	Method	Positif.m

Acquisition Parameter

Source Type	ESI	Ion Polarity	Positive	Set Nebulizer	0.6 Bar
Scan Begin	50 m/z	Set Capillary	4500 V	Set Dry Heater	200 °C
Scan End	3000 m/z	Set Collision Cell RF	1800.0 Vpp	Set Dry Gas	7.0 l/min

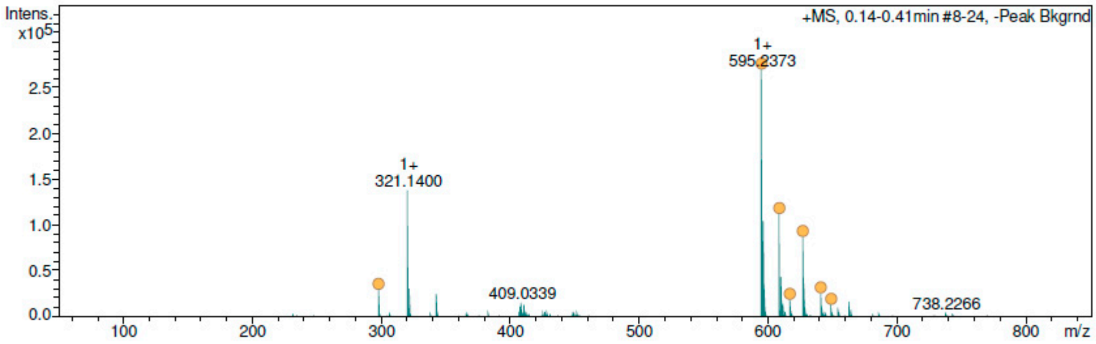

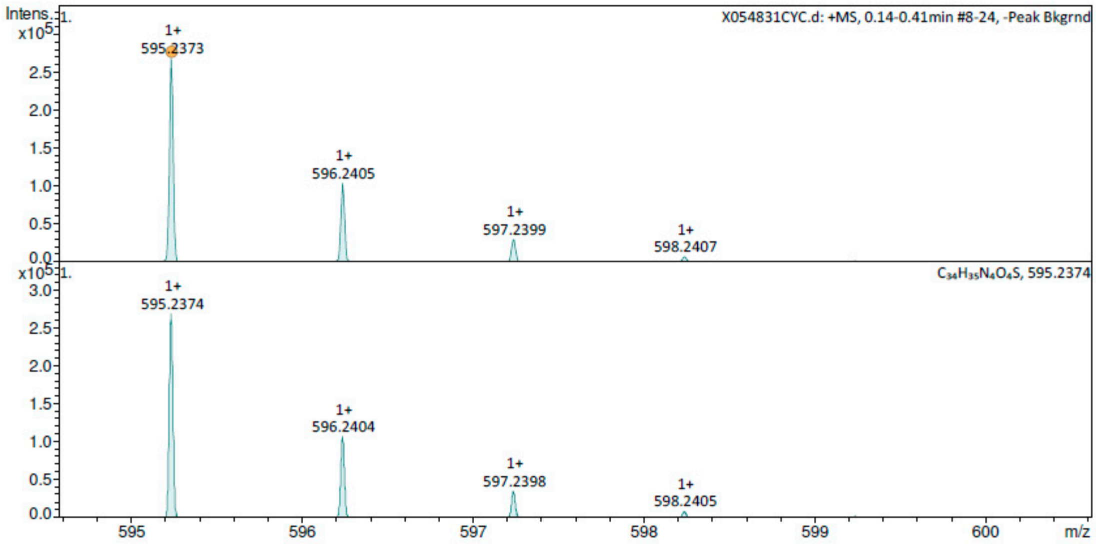

Meas. m/z	z	#	Ion Formula	m/z	err [ppm]	mSigma	rdb	e^- Conf
298.122444	2+	1	C34H36N4O4S	298.122315	-0.4	7.4	20.0	even
	2+	2	C33H40O8S	298.121646	-2.7	19.0	15.0	even
595.237310	1+	1	C34H35N4O4S	595.237353	0.1	10.6	20.0	even
609.252938	1+	1	C35H37N4O4S	609.253003	0.1	51.0	20.0	even
617.219178	1+	1	C33H38NaO8S	617.217960	-2.0	5.2	15.0	even
	1+	2	C34H34N4NaO4S	617.219297	0.2	13.7	20.0	even
627.263450	1+	1	C35H39N4O5S	627.263568	0.2	7.4	19.0	even
	1+	2	C34H43O9S	627.262230	-1.9	15.3	14.0	even

Institut de Chimie Organique et Analytique
UMR 7311 - Université d'Orléans - CNRS
BP 6759 - rue de Chartres - F-45067 ORLEANS Cedex 2
cyril.colas@univ-orleans.fr
+33 (0)2 38 49 46 61

printed: 17/02/2020 18:33:23
Page 1 of 2

Figure S10. Mass spectra of Compound **6.**

Fédération de Recherche Physique et Chimie du Vivant (FR2708 : CBM/ICOA)
Plate-forme de Spectrométrie de Masse Haute Résolution

HRAM

Analysis Info

Sample Name	**RSW184**	Acquisition Date	12/02/2020 13:51:25
Analysis Name	X054832CYC.d	Instrument / Ser#	maXis 255552.00086
		Method	Positif.m

Acquisition Parameter

Source Type	ESI	Ion Polarity	Positive	Set Nebulizer	0.6 Bar
Scan Begin	50 m/z	Set Capillary	4500 V	Set Dry Heater	200 °C
Scan End	3000 m/z	Set Collision Cell RF	1800.0 Vpp	Set Dry Gas	7.0 l/min

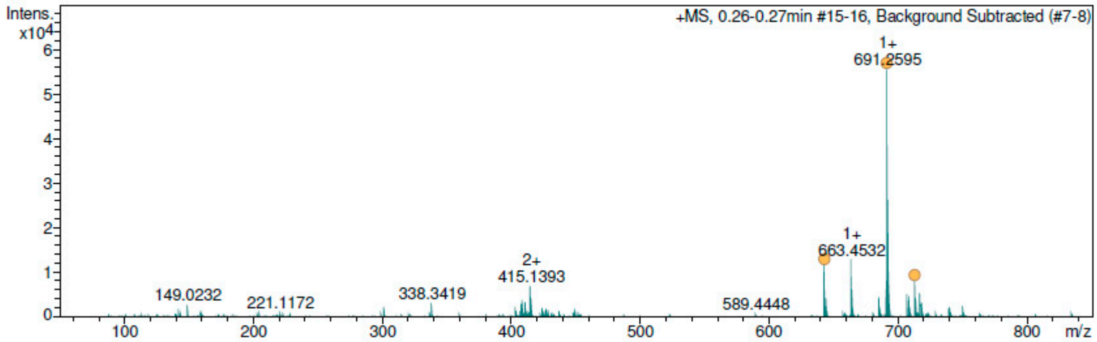

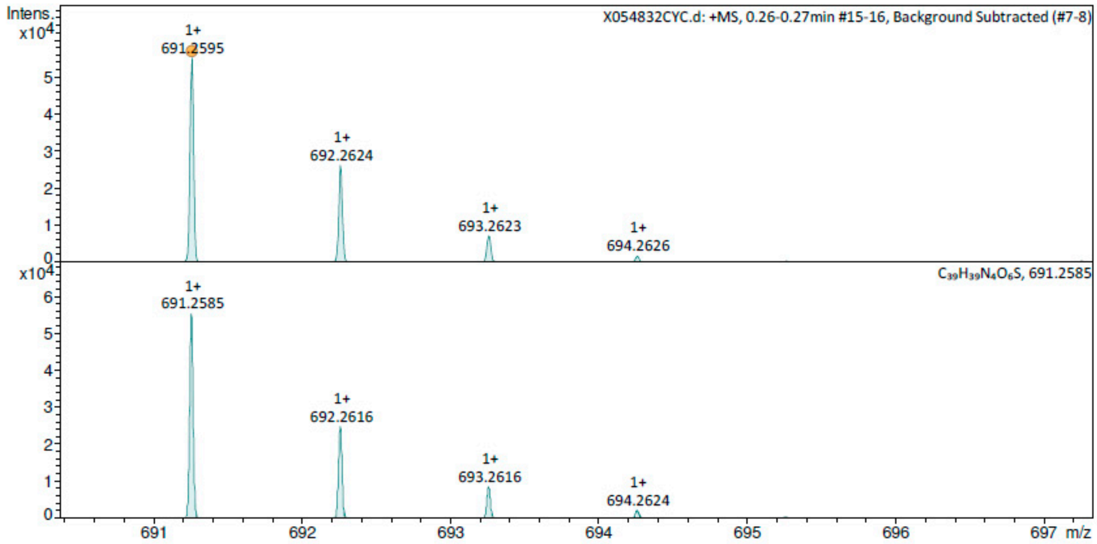

Meas. m/z	z	#	Ion Formula	m/z	err [ppm]	mSigma	rdb	e⁻ Conf
643.248252	1+	1	C36H39N2O7S	643.247249	-1.6	23.1	19.0	even
	1+	2	C37H35N6O3S	643.248586	0.5	34.2	24.0	even
691.259501	1+	1	C39H39N4O6S	691.258482	-1.5	15.9	23.0	even
713.241251	1+	1	C39H38N4NaO6S	713.240427	-1.2	29.4	23.0	even

Institut de Chimie Organique et Analytique
UMR 7311 - Université d'Orléans - CNRS
BP 6759 - rue de Chartres - F-45067 ORLEANS Cedex 2
cyril.colas@univ-orleans.fr
+33 (0)2 38 49 46 61

printed: 17/02/2020 18:36:20
Page 1 of 1

Figure S11. Mass spectra of Compound **7a.**

Fédération de Recherche Physique et Chimie du Vivant (FR2708 : CBM/ICOA)
Plate-forme de Spectrométrie de Masse Haute Résolution

HRAM

Analysis Info

Sample Name	**RSW225**	Acquisition Date	12/02/2020 14:12:20
Analysis Name	X054846CYC.d	Instrument / Ser#	maXis 255552.00086
		Method	Positif.m

Acquisition Parameter

Source Type	ESI	Ion Polarity	Positive	Set Nebulizer	0.6 Bar
Scan Begin	50 m/z	Set Capillary	4500 V	Set Dry Heater	200 °C
Scan End	3000 m/z	Set Collision Cell RF	1800.0 Vpp	Set Dry Gas	7.0 l/min

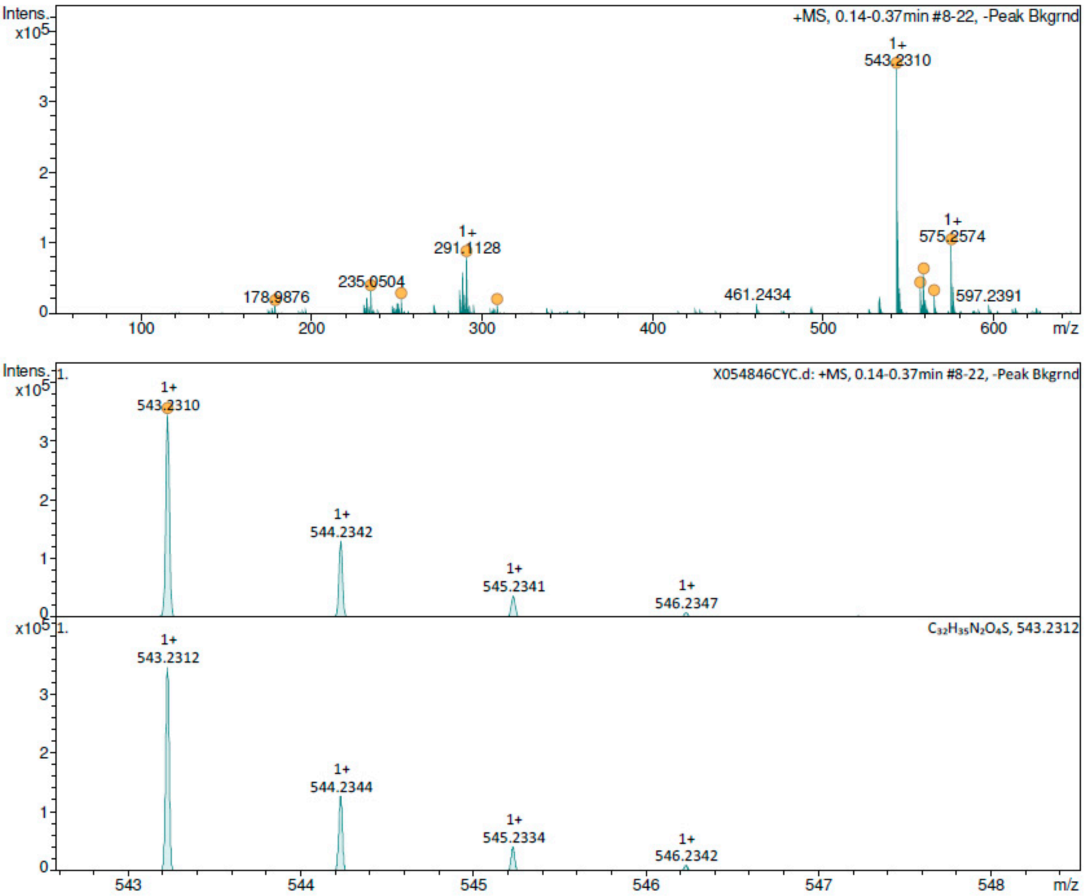

Meas. m/z	z	#	Ion Formula	m/z	err [ppm]	mSigma	rdb	e⁻ Conf
178.987612	1+	1	C4H11Sn	178.987721	0.9	22.4	1.0	even
235.050365	1+	1	C8H19Sn	235.050322	0.3	9.0	1.0	even
253.060975	1+	1	C8H21OSn	253.060886	0.1	22.3	0.0	even
291.112827	1+	1	C12H27Sn	291.112922	1.0	11.2	1.0	even
309.123448	1+	1	C12H29OSn	309.123487	0.8	19.8	0.0	even
543.231021	1+	1	C32H35N2O4S	543.231205	0.3	8.0	17.0	even
557.246664	1+	1	C33H37N2O4S	557.246855	0.3	62.8	17.0	even
559.226623	1+	1	C32H35N2O5S	559.226120	-0.9	7.0	17.0	even

Institut de Chimie Organique et Analytique
UMR 7311 - Université d'Orléans - CNRS
BP 6759 - rue de Chartres - F-45067 ORLEANS Cedex 2
cyril.colas@univ-orleans.fr
+33 (0)2 38 49 46 61

printed: 18/02/2020 18:10:15

Figure S12. Mass spectra of Compound **8a.**

Fédération de Recherche Physique et Chimie du Vivant (FR2708 : CBM/ICOA)
Plate-forme de Spectrométrie de Masse Haute Résolution

HRAM

Analysis Info

Sample Name	**RSW226**	Acquisition Date	12/02/2020 17:08:28
Analysis Name	X054847CYC_31576.d	Instrument / Ser#	maXis 255552.00086
		Method	Positif.m

Acquisition Parameter

Source Type	ESI	Ion Polarity	Positive	Set Nebulizer	0.6 Bar
Scan Begin	50 m/z	Set Capillary	4500 V	Set Dry Heater	200 °C
Scan End	3000 m/z	Set Collision Cell RF	1800.0 Vpp	Set Dry Gas	7.0 l/min

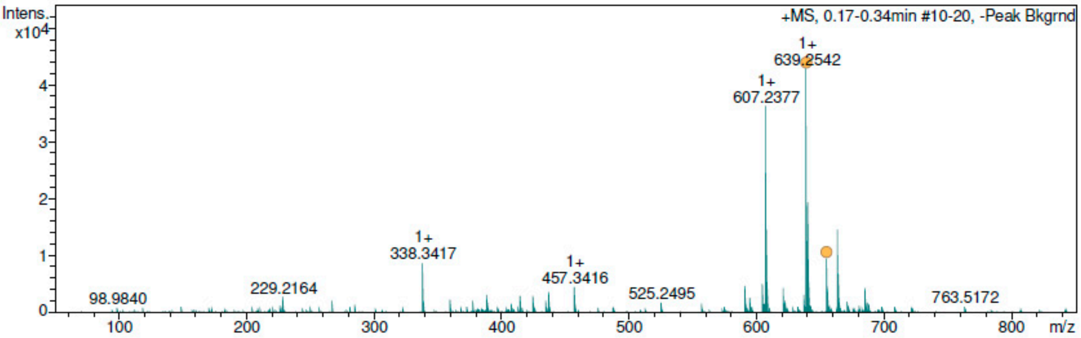

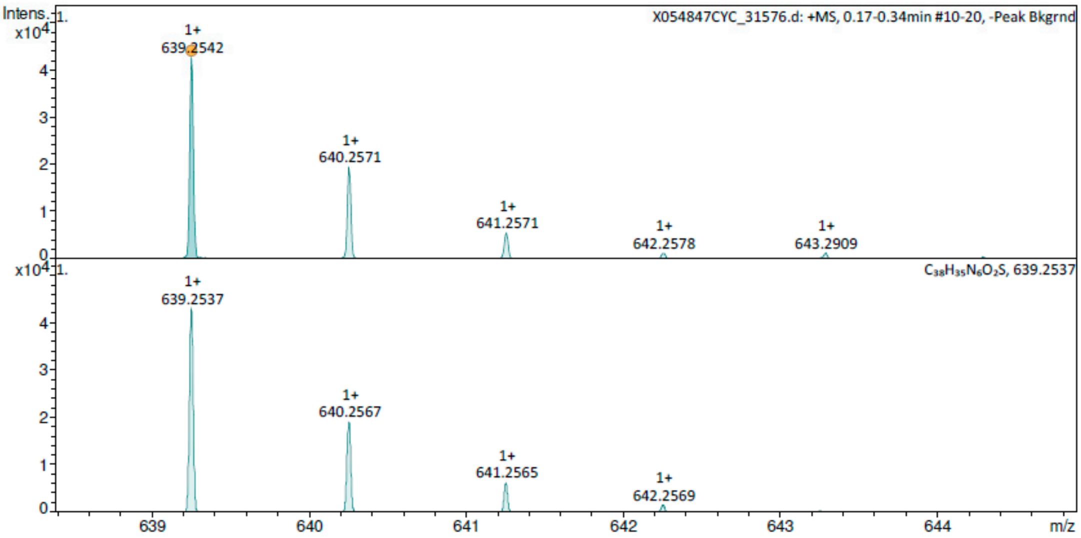

Meas. m/z	z	#	Ion Formula	m/z	err [ppm]	mSigma	rdb	e⁻ Conf
639.254155	1+	1	C38H35N6O2S	639.253672	-0.8	10.2	25.0	even
655.249022	1+	1	C38H35N6O3S	655.248586	-0.7	21.6	25.0	even

Institut de Chimie Organique et Analytique
UMR 7311 - Université d'Orléans - CNRS
BP 6759 - rue de Chartres - F-45067 ORLEANS Cedex 2
cyril.colas@univ-orleans.fr
+33 (0)2 38 49 46 61

printed: 18/02/2020 18:12:33
Page 1 of 1

Figure S13. Mass spectra of Compound **7b.**

Fédération de Recherche Physique et Chimie du Vivant (FR2708 : CBM/ICOA)
Plate-forme de Spectrométrie de Masse Haute Résolution

HRAM

Analysis Info

Sample Name	**RSW195**	Acquisition Date	12/02/2020 13:55:54
Analysis Name	X054835CYC.d	Instrument / Ser#	maXis 255552.00086
		Method	Positif.m

Acquisition Parameter

Source Type	ESI	Ion Polarity	Positive	Set Nebulizer	0.6 Bar
Scan Begin	50 m/z	Set Capillary	4500 V	Set Dry Heater	200 °C
Scan End	3000 m/z	Set Collision Cell RF	1800.0 Vpp	Set Dry Gas	7.0 l/min

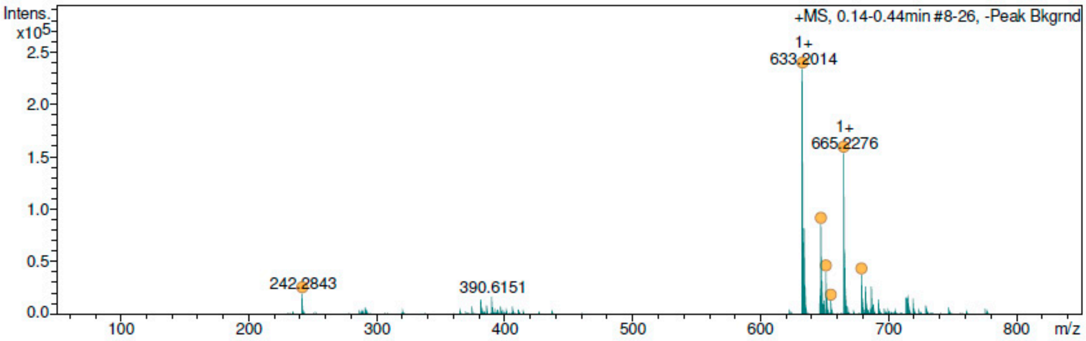

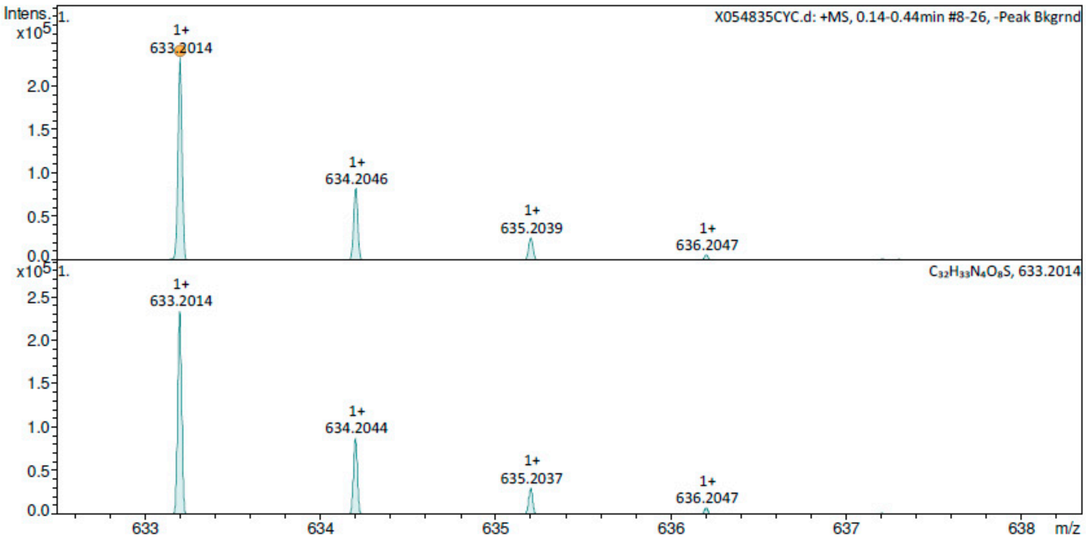

Meas. m/z	z	#	Ion Formula	m/z	err [ppm]	mSigma	rdb	e⁻ Conf
242.284299	1+	1	C16H36N	242.284227	-0.3	1.7	0.0	even
633.201382	1+	1	C32H33N4O8S	633.201361	-0.0	15.6	19.0	even
647.217157	1+	1	C33H35N4O8S	647.217012	-0.2	64.9	19.0	even
651.213147	1+	1	C32H35N4O9S	651.211926	-1.9	8.6	18.0	even
655.183673	1+	1	C32H32N4NaO8S	655.183306	-0.6	17.7	19.0	even
665.227592	1+	1	C33H37N4O9S	665.227576	-0.0	12.3	18.0	even
	1+	2	C32H41O13S	665.226239	-2.0	15.2	13.0	even
679.243190	1+	1	C34H39N4O9S	679.243226	0.1	325.6	18.0	even

Institut de Chimie Organique et Analytique
UMR 7311 - Université d'Orléans - CNRS
BP 6759 - rue de Chartres - F-45067 ORLEANS Cedex 2

cyril.colas@univ-orleans.fr
+33 (0)2 38 49 46 61

printed: 17/02/2020 18:44:30
Page 1 of 2

Figure S14. Mass spectra of Compound **8b.**

Fédération de Recherche Physique et Chimie du Vivant (FR2708 : CBM/ICOA)
Plate-forme de Spectrométrie de Masse Haute Résolution

HRAM

Analysis Info

Sample Name	**RSW197**	Acquisition Date	12/02/2020 16:59:01
Analysis Name	X054836CYC_31570.d	Instrument / Ser#	maXis 255552.00086
		Method	Positif.m

Acquisition Parameter

Source Type	ESI	Ion Polarity	Positive	Set Nebulizer	0.6 Bar
Scan Begin	50 m/z	Set Capillary	4500 V	Set Dry Heater	200 °C
Scan End	3000 m/z	Set Collision Cell RF	1800.0 Vpp	Set Dry Gas	7.0 l/min

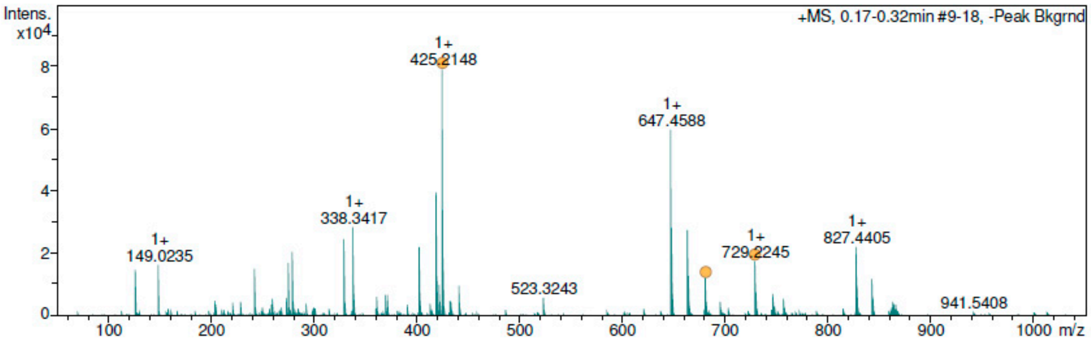

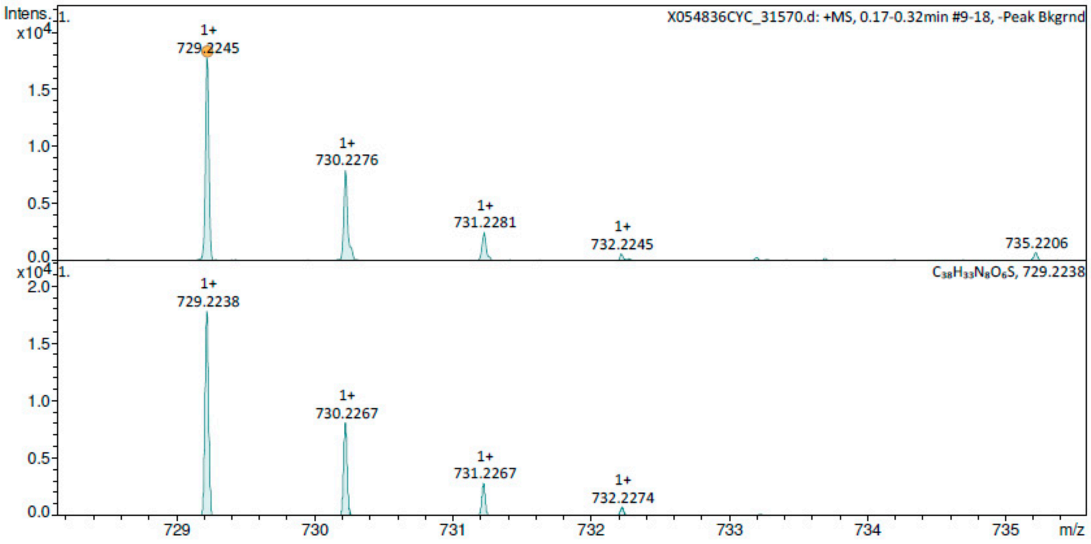

Meas. m/z	z	#	Ion Formula	m/z	err [ppm]	mSigma	rdb	e⁻ Conf
425.214841	1+	1	C19H25N10O2	425.215647	1.9	6.3	13.0	even
	1+	2	C18H29N6O6	425.214309	-1.3	7.6	8.0	even
681.212798	1+	1	C35H33N6O7S	681.212595	-0.3	26.1	23.0	even
	1+	2	C36H29N10O3S	681.213932	1.7	36.8	28.0	even
729.224474	1+	1	C38H33N8O6S	729.223828	-0.9	10.1	27.0	even

Institut de Chimie Organique et Analytique
UMR 7311 - Université d'Orléans - CNRS
BP 6759 - rue de Chartres - F-45067 ORLEANS Cedex 2
cyril.colas@univ-orleans.fr
+33 (0)2 38 49 46 61

printed: 17/02/2020 18:53:43
Page 1 of 1

Figure S15. Mass spectra of Compound **18a.**

Fédération de Recherche Physique et Chimie du Vivant (FR2708 : CBM/ICOA)
Plate-forme de Spectrométrie de Masse Haute Résolution

HRAM

Analysis Info

Sample Name	**RSW206**	Acquisition Date	12/02/2020 13:58:50
Analysis Name	X054837CYC.d	Instrument / Ser#	maXis 255552.00086
		Method	Positif.m

Acquisition Parameter

Source Type	ESI	Ion Polarity	Positive	Set Nebulizer	0.6 Bar
Scan Begin	50 m/z	Set Capillary	4500 V	Set Dry Heater	200 °C
Scan End	3000 m/z	Set Collision Cell RF	1800.0 Vpp	Set Dry Gas	7.0 l/min

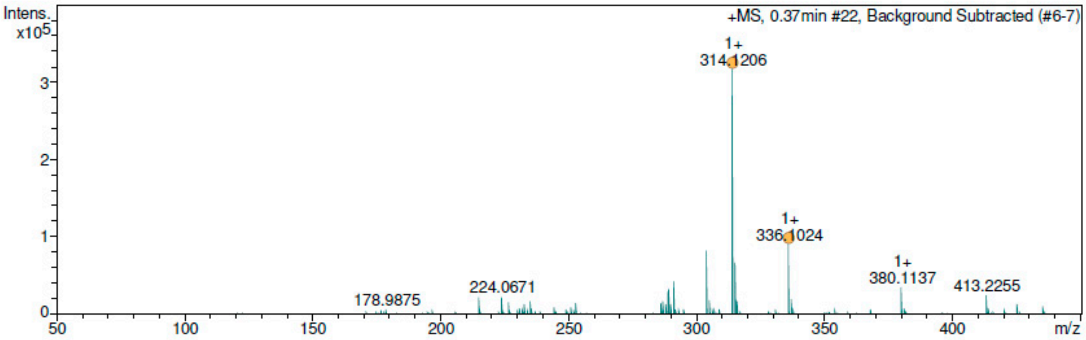

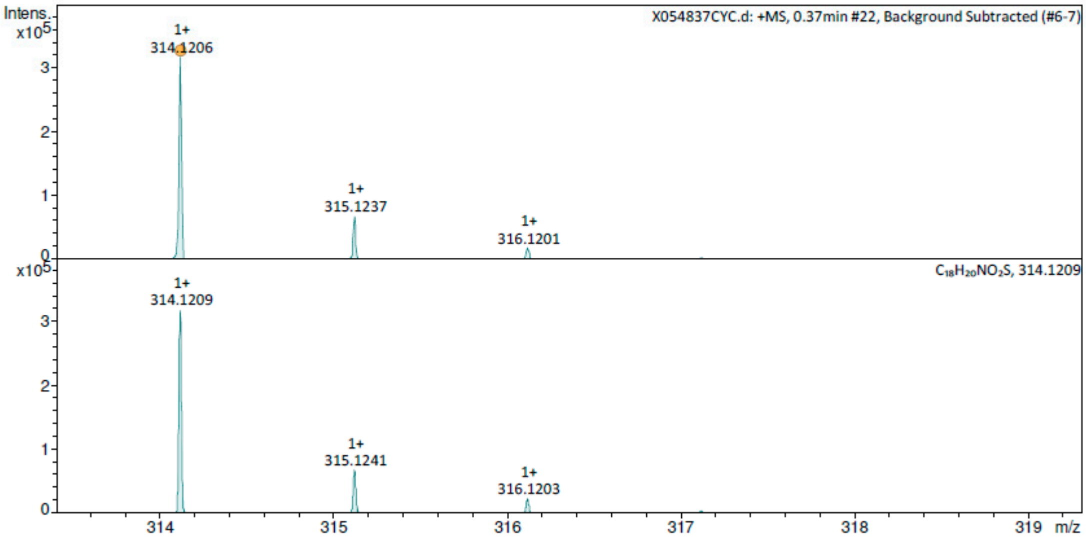

Meas. m/z	z	#	Ion Formula	m/z	err [ppm]	mSigma	rdb	e⁻ Conf
314.120593	1+	1	C18H20NO2S	314.120926	1.1	7.1	10.0	even
336.102422	1+	1	C18H19NNaO2S	336.102871	1.3	6.7	10.0	even

Institut de Chimie Organique et Analytique
UMR 7311 - Université d'Orléans - CNRS
BP 6759 - rue de Chartres - F-45067 ORLEANS Cedex 2
cyril.colas@univ-orleans.fr
+33 (0)2 38 49 46 61

printed: 17/02/2020 18:54:54
Page 1 of 1

Figure S16. Mass spectra of Compound **19a.**

Fédération de Recherche Physique et Chimie du Vivant (FR2708 : CBM/ICOA)
Plate-forme de Spectrométrie de Masse Haute Résolution

HRAM

Analysis Info

Sample Name	**RSW208**	Acquisition Date	12/02/2020 14:00:20
Analysis Name	X054838CYC.d	Instrument / Ser#	maXis 255552.00086
		Method	Positif.m

Acquisition Parameter

Source Type	ESI	Ion Polarity	Positive	Set Nebulizer	0.6 Bar
Scan Begin	50 m/z	Set Capillary	4500 V	Set Dry Heater	200 °C
Scan End	3000 m/z	Set Collision Cell RF	1800.0 Vpp	Set Dry Gas	7.0 l/min

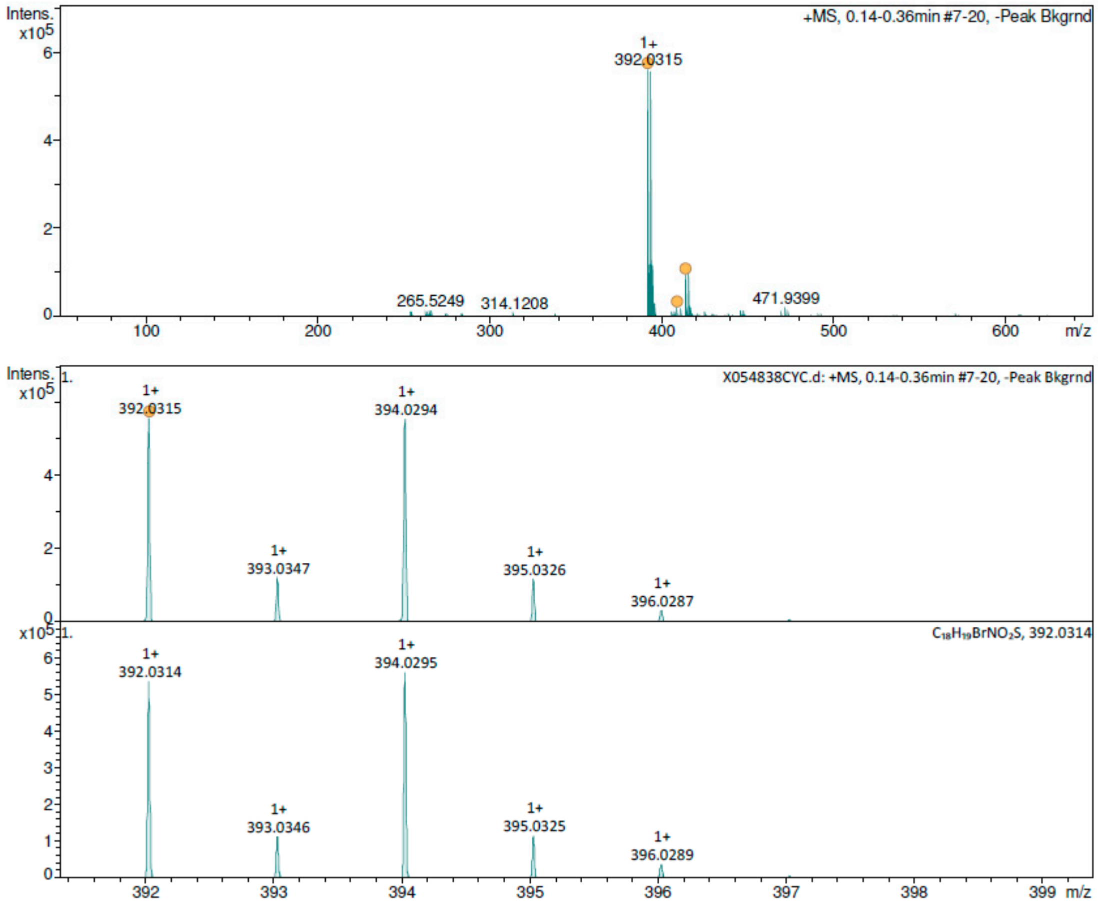

Meas. m/z	z	#	Ion Formula	m/z	err [ppm]	mSigma	rdb	e⁻ Conf
392.031453	1+	1	C18H19BrNO2S	392.031438	-0.0	20.1	10.0	even
409.056750	1+	1	C18H22BrN2O2S	409.057987	3.0	39.0	9.0	even
414.013476	1+	1	C18H18BrNNaO2S	414.013383	-0.2	5.6	10.0	even

Institut de Chimie Organique et Analytique
UMR 7311 - Université d'Orléans - CNRS
BP 6759 - rue de Chartres - F-45067 ORLEANS Cedex 2
cyril.colas@univ-orleans.fr
+33 (0)2 38 49 46 61
printed: 17/02/2020 18:56:23
Page 1 of 1

Figure S17. Mass spectra of Compound **9a.**

Fédération de Recherche Physique et Chimie du Vivant (FR2708 : CBM/ICOA)
Plate-forme de Spectrométrie de Masse Haute Résolution

HRAM

Analysis Info

Sample Name	**RSW209**	Acquisition Date	12/02/2020 17:00:52
Analysis Name	X054839CYC_31571.d	Instrument / Ser#	maXis 255552.00086
		Method	Positif.m

Acquisition Parameter

Source Type	ESI	Ion Polarity	Positive	Set Nebulizer	0.6 Bar
Scan Begin	50 m/z	Set Capillary	4500 V	Set Dry Heater	200 °C
Scan End	3000 m/z	Set Collision Cell RF	1800.0 Vpp	Set Dry Gas	7.0 l/min

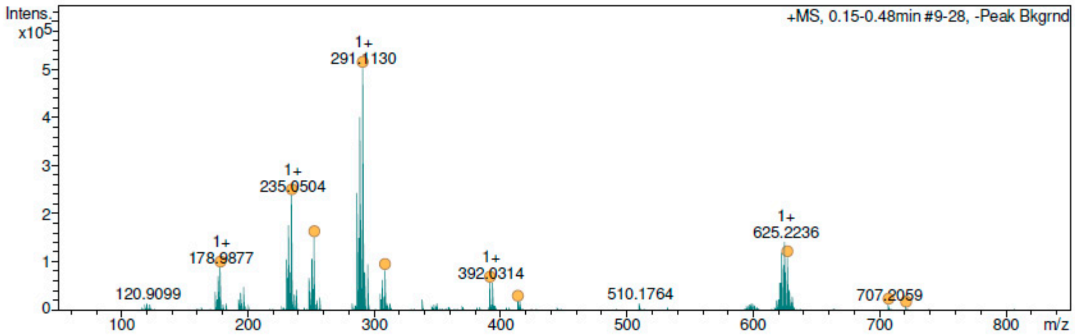

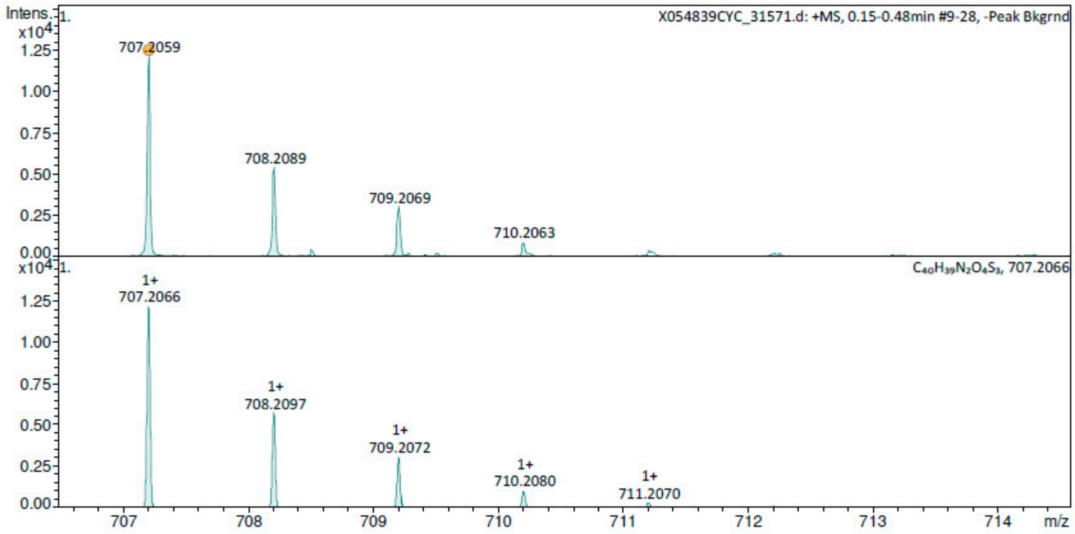

Meas. m/z	z	#	Ion Formula	m/z	err [ppm]	mSigma	rdb	e⁻ Conf
178.987707	1+	1	C4H11Sn	178.987721	0.4	19.2	1.0	even
235.050415	1+	1	C8H19Sn	235.050322	0.1	6.3	1.0	even
253.060961	1+	1	C8H21OSn	253.060886	0.2	11.9	0.0	even
291.113031	1+	1	C12H27Sn	291.112922	0.3	22.8	1.0	even
309.123437	1+	1	C12H29OSn	309.123487	0.8	13.0	0.0	even
392.031372	1+	1	C18H19BrNO2S	392.031438	0.2	22.3	10.0	even
414.013196	1+	1	C18H18BrNNaO2S	414.013383	0.5	6.7	10.0	even
627.224699	1+	1	C25H55O2Sn2	627.224047	0.7	16.1	1.0	even

Institut de Chimie Organique et Analytique
UMR 7311 - Université d'Orléans - CNRS
BP 6759 - rue de Chartres - F-45067 ORLEANS Cedex 2
cyril.colas@univ-orleans.fr
+33 (0)2 38 49 46 61

printed: 18/02/2020 14:29:38
Page 1 of 2

Meas. m/z	z	#	Ion Formula	m/z	err [ppm]	mSigma	rdb	e⁻ Conf
707.205942	1+	1	C40H39N2O4S3	707.206647	1.0	17.2	23.0	even
721.222719	1+	1	C41H41N2O4S3	721.222297	-0.6	39.4	23.0	even

Figure S18. Mass spectra of Compound **10a.**

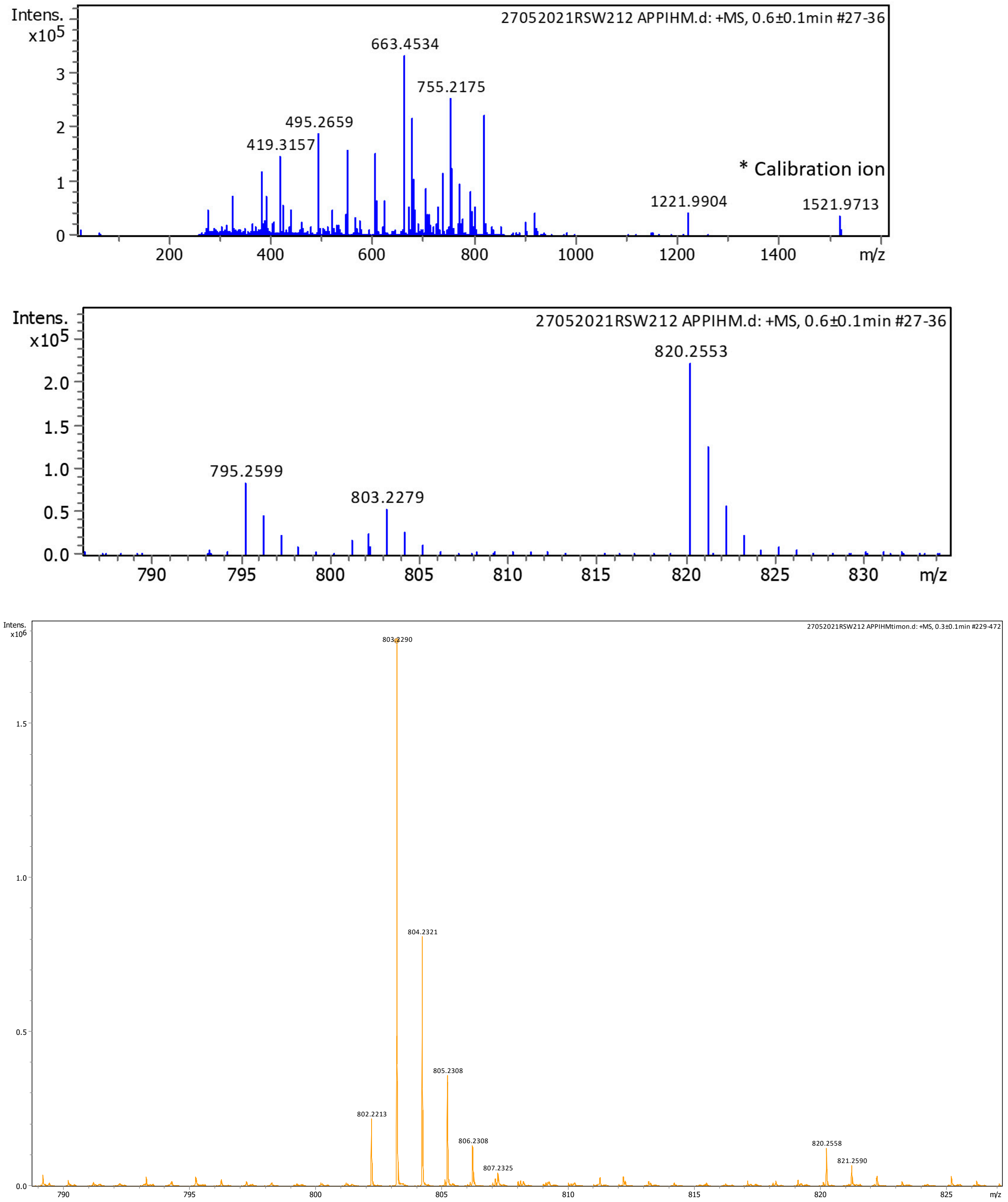

Figure S19. Mass spectra of Compound **18b.**

Fédération de Recherche Physique et Chimie du Vivant (FR2708 : CBM/ICOA)
Plate-forme de Spectrométrie de Masse Haute Résolution

HRAM

Analysis Info

Sample Name	**RSW218**	Acquisition Date	12/02/2020 14:04:52
Analysis Name	X054841CYC.d	Instrument / Ser#	maXis 255552.00086
		Method	Positif.m

Acquisition Parameter

Source Type	ESI	Ion Polarity	Positive	Set Nebulizer	0.6 Bar
Scan Begin	50 m/z	Set Capillary	4500 V	Set Dry Heater	200 °C
Scan End	3000 m/z	Set Collision Cell RF	1800.0 Vpp	Set Dry Gas	7.0 l/min

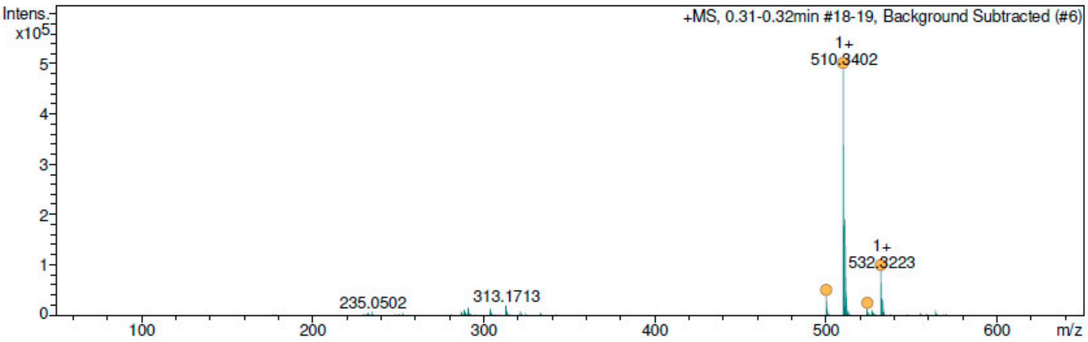

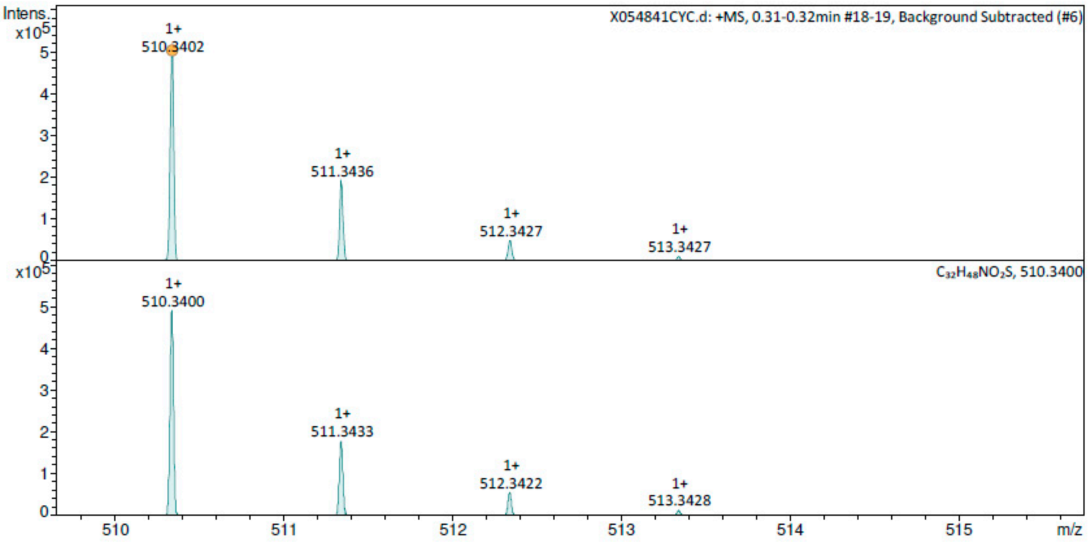

Meas. m/z	z	#	Ion Formula	m/z	err [ppm]	mSigma	rdb	e⁻ Conf
500.356006	1+	1	$C_{31}H_{50}NO_2S$	500.355677	-0.7	13.3	8.0	even
510.340162	1+	1	$C_{32}H_{48}NO_2S$	510.340027	-0.3	15.2	10.0	even
524.355755	1+	1	$C_{33}H_{50}NO_2S$	524.355677	-0.1	412.1	10.0	even
532.322287	1+	1	$C_{32}H_{47}NNaO_2S$	532.321971	-0.6	6.9	10.0	even

Institut de Chimie Organique et Analytique
UMR 7311 - Université d'Orléans - CNRS
BP 6759 - rue de Chartres - F-45067 ORLEANS Cedex 2
cyril.colas@univ-orleans.fr
+33 (0)2 38 49 46 61

printed: 18/02/2020 17:52:19
Page 1 of 1

Figure S20. Mass spectra of Compound **19b.**

Fédération de Recherche Physique et Chimie du Vivant (FR2708 : CBM/ICOA)
Plate-forme de Spectrométrie de Masse Haute Résolution

HRAM

Analysis Info

Sample Name	**RSW219**	Acquisition Date	12/02/2020 14:06:20
Analysis Name	X054842CYC.d	Instrument / Ser#	maXis 255552.00086
		Method	Positif.m

Acquisition Parameter

Source Type	ESI	Ion Polarity	Positive	Set Nebulizer	0.6 Bar
Scan Begin	50 m/z	Set Capillary	4500 V	Set Dry Heater	200 °C
Scan End	3000 m/z	Set Collision Cell RF	1800.0 Vpp	Set Dry Gas	7.0 l/min

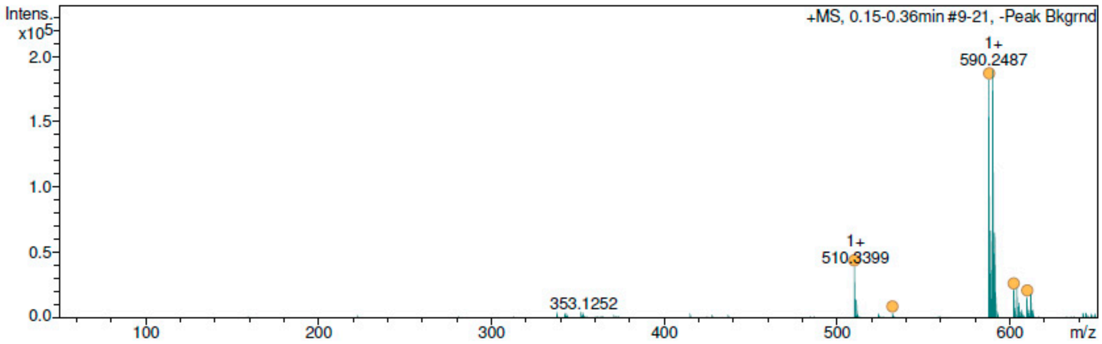

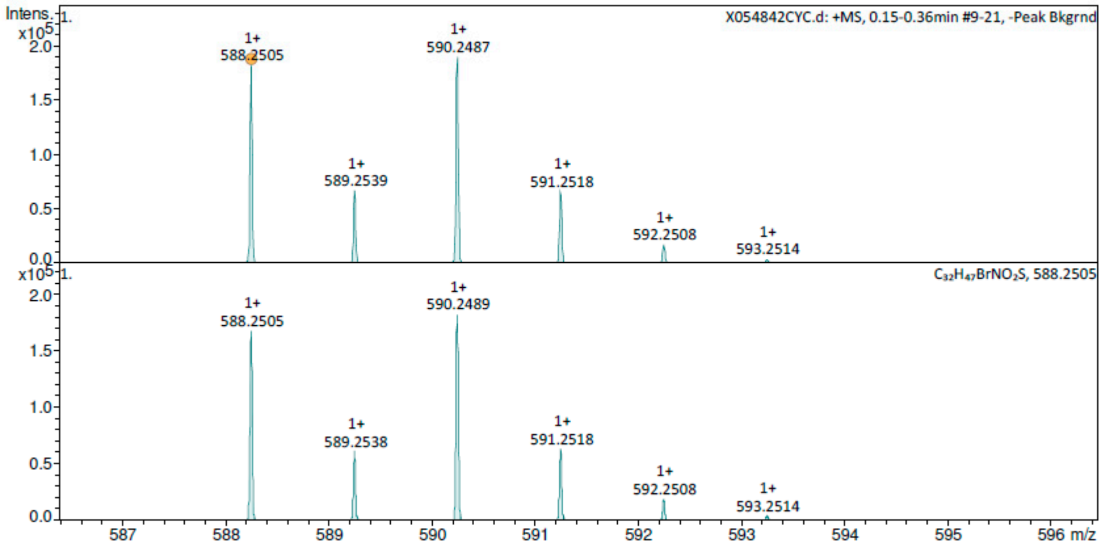

Meas. m/z	z	#	Ion Formula	m/z	err [ppm]	mSigma	rdb	e⁻ Conf
510.339931	1+	1	C32H48NO2S	510.340027	0.2	8.7	10.0	even
532.322583	1+	1	C32H47NNaO2S	532.321971	-1.1	60.7	10.0	even
588.250512	1+	1	C32H47BrNO2S	588.250539	0.0	19.0	10.0	even
602.265973	1+	1	C33H49BrNO2S	602.266189	0.4	120.0	10.0	even
610.232554	1+	1	C32H46BrNNaO2S	610.232484	-0.1	13.0	10.0	even

Institut de Chimie Organique et Analytique
UMR 7311 - Université d'Orléans - CNRS
BP 6759 - rue de Chartres - F-45067 ORLEANS Cedex 2
cyril.colas@univ-orleans.fr
+33 (0)2 38 49 46 61

printed: 18/02/2020 17:54:20
Page 1 of 1

Figure S21. Mass spectra of Compound **9b.**

Fédération de Recherche Physique et Chimie du Vivant (FR2708 : CBM/ICOA)
Plate-forme de Spectrométrie de Masse Haute Résolution

HRAM

Analysis Info

Sample Name	**RSW222**	Acquisition Date	12/02/2020 17:05:24
Analysis Name	X054844CYC_31574.d	Instrument / Ser#	maXis 255552.00086
		Method	Positif.m

Acquisition Parameter

Source Type	ESI	Ion Polarity	Positive	Set Nebulizer	0.6 Bar
Scan Begin	50 m/z	Set Capillary	4500 V	Set Dry Heater	200 °C
Scan End	3000 m/z	Set Collision Cell RF	1800.0 Vpp	Set Dry Gas	7.0 l/min

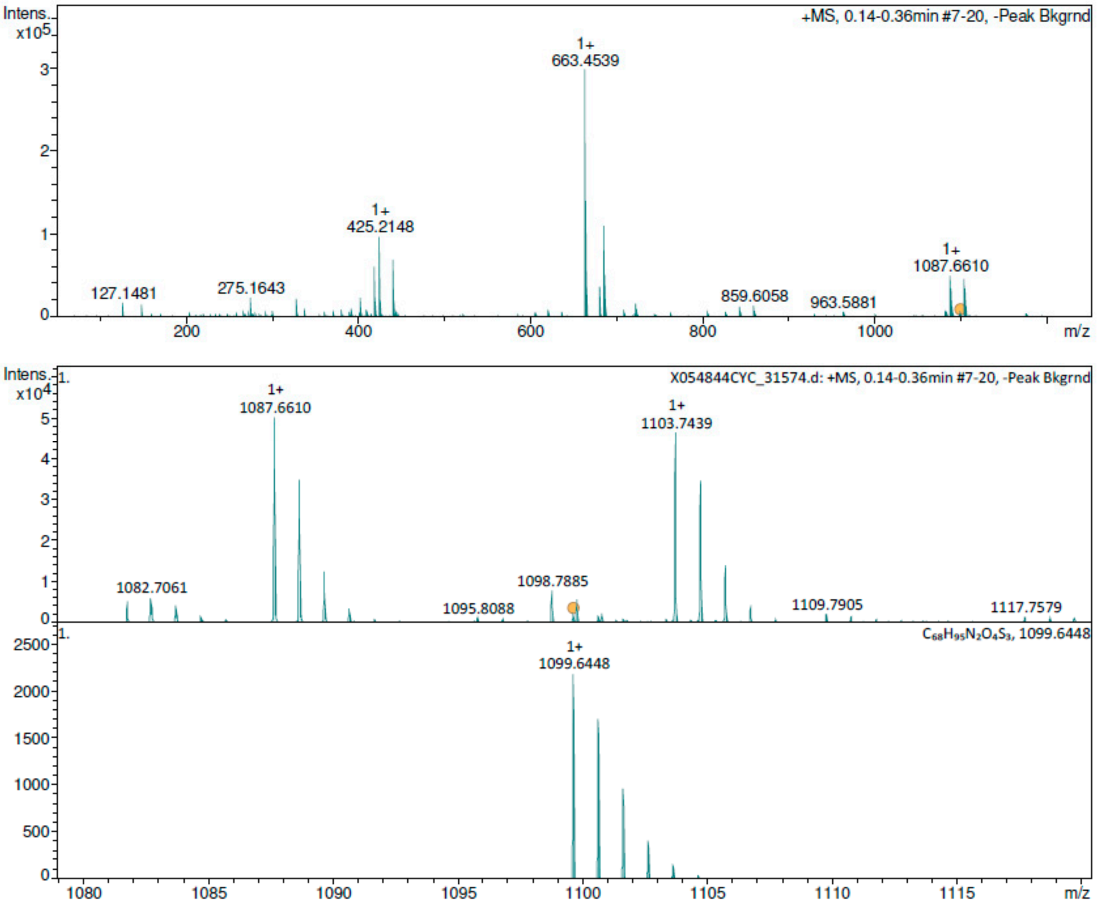

Meas. m/z	z	#	Ion Formula	m/z	err [ppm]	mSigma	rdb	e⁻ Conf
1099.640606	1+	1	C62H99O10S3	1099.639489	-1.0	132.7	14.0	even
	1+	2	C63H95N4O6S3	1099.640826	0.2	132.7	19.0	even
	1+	3	C68H95N2O4S3	1099.644849	3.9	137.1	23.0	even

Institut de Chimie Organique et Analytique
UMR 7311 - Université d'Orléans - CNRS
BP 6759 - rue de Chartres - F-45067 ORLEANS Cedex 2
cyril.colas@univ-orleans.fr
+33 (0)2 38 49 46 61

printed: 18/02/2020 18:04:08
Page 1 of 1

Figure S22. Mass spectra of Compound **10b.**

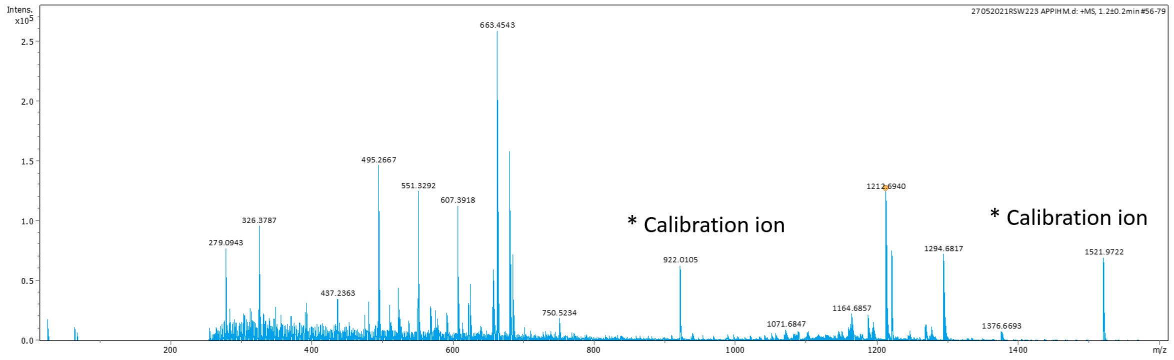

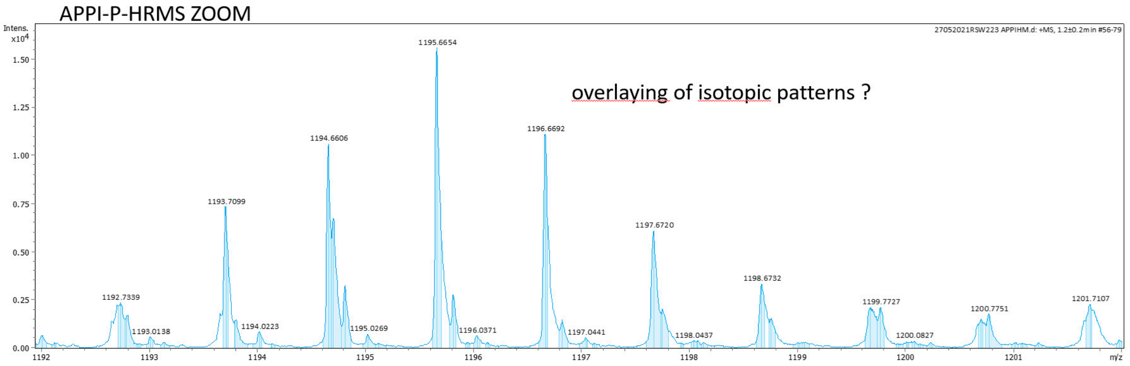

Meas. m/z	Ion Formula	m/z	err [ppm]	mSigma	Score	rdb	e$^-$ Conf	N-Rule
1195.6653	C74H95N6O2S3	1195.6673	1.7	2	100.00	37.0	even	ok

9. ¹H and ¹³C NMR spectra

NMR spectra of the obtained compounds are shown on the following pages.

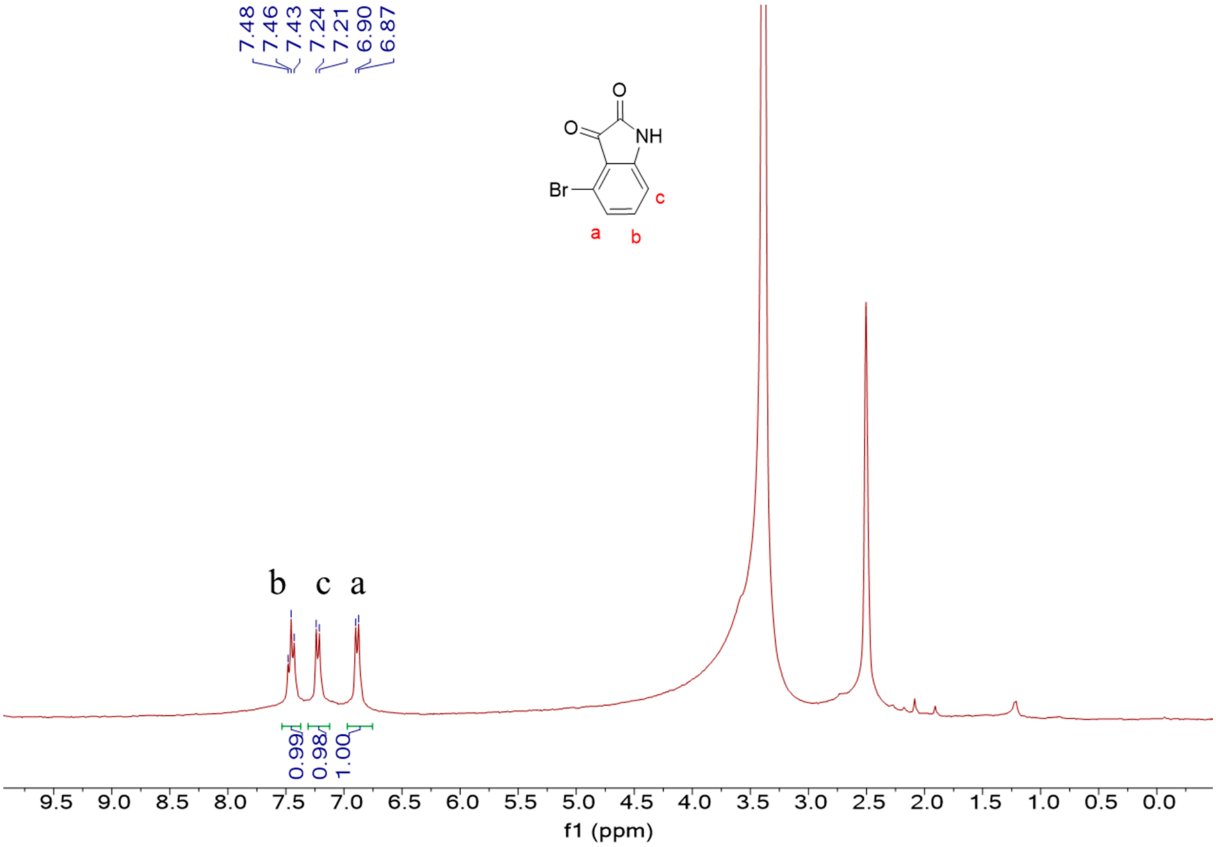

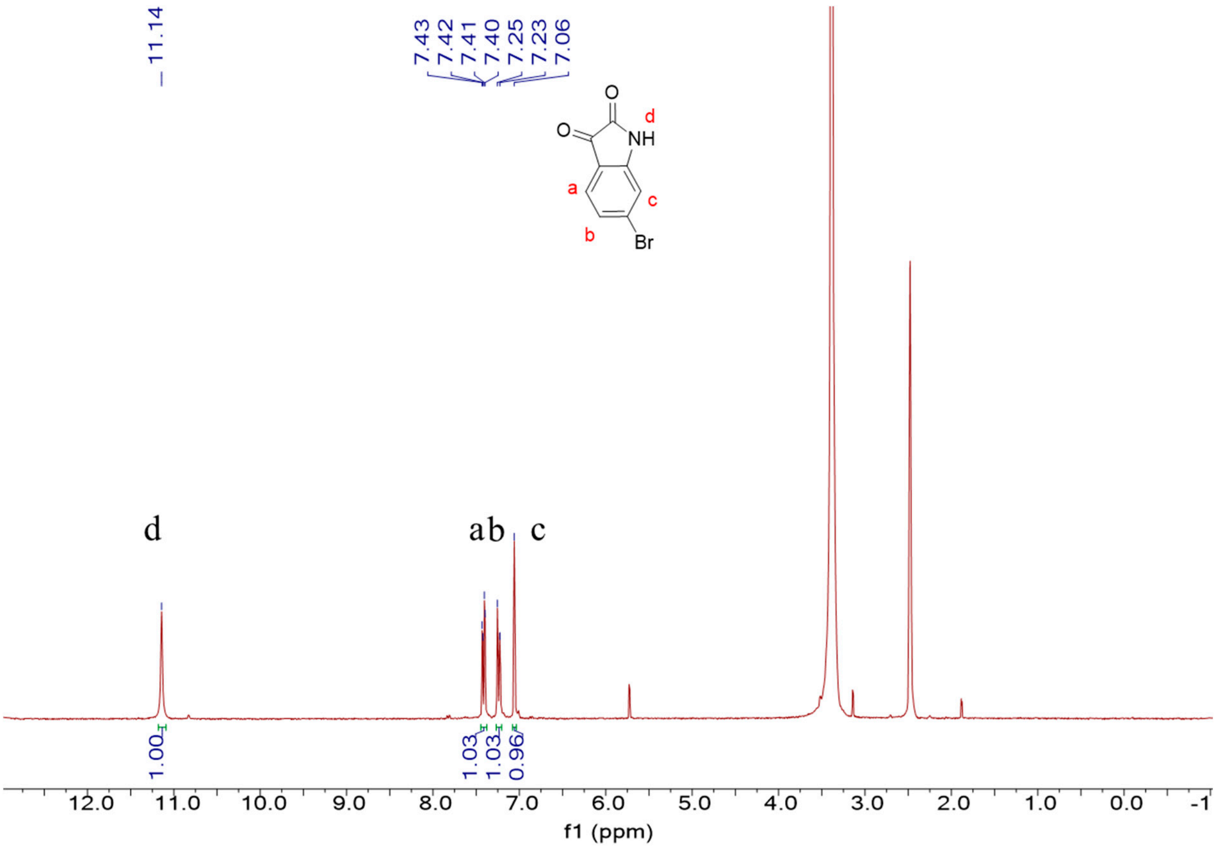

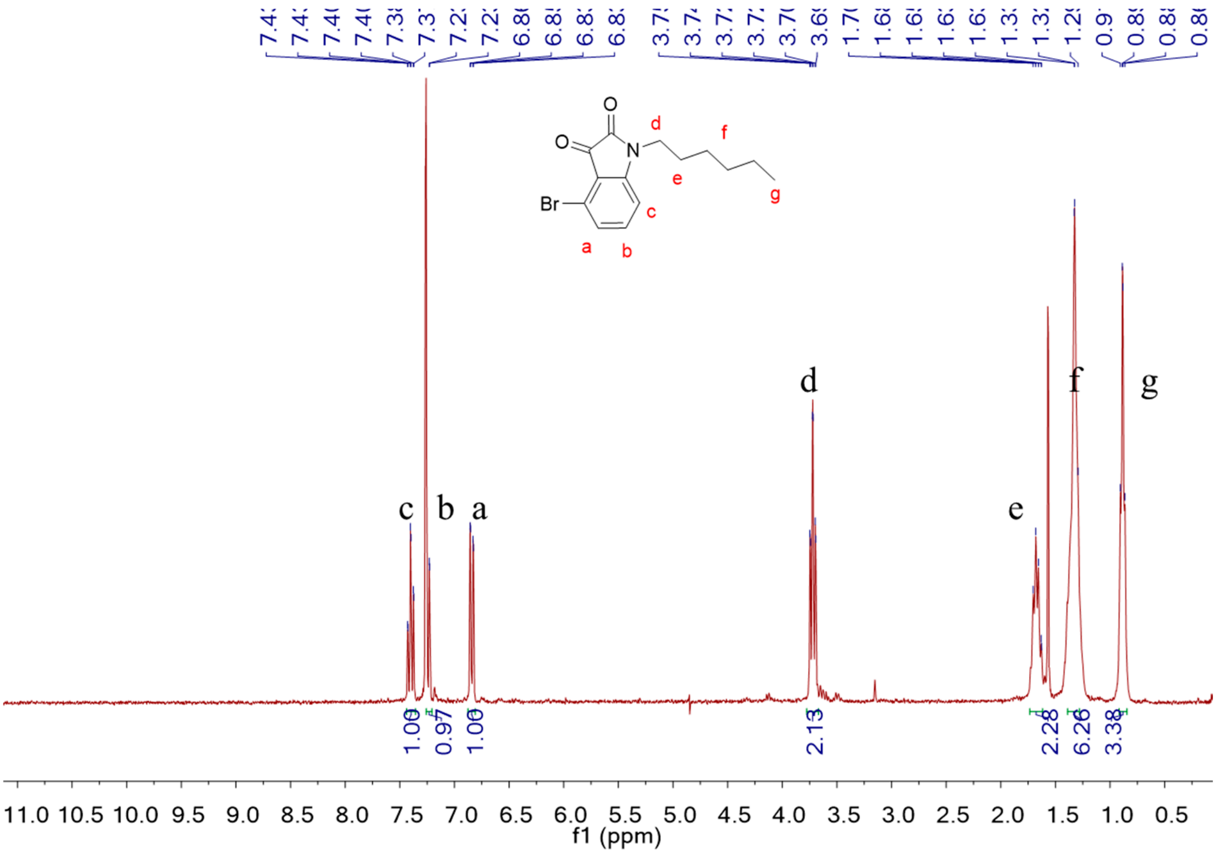
d
f
e
g
c
a
b
Br
O
N
c b a
d
e
f
g
1.00
0.97
1.00
2.13
2.28
6.26
3.38
11.0 10.5 10.0 9.5 9.0 8.5 8.0 7.5 7.0 6.5 6.0 5.5 5.0 4.5 4.0 3.5 3.0 2.5 2.0 1.5 1.0 0.5
f1 (ppm)

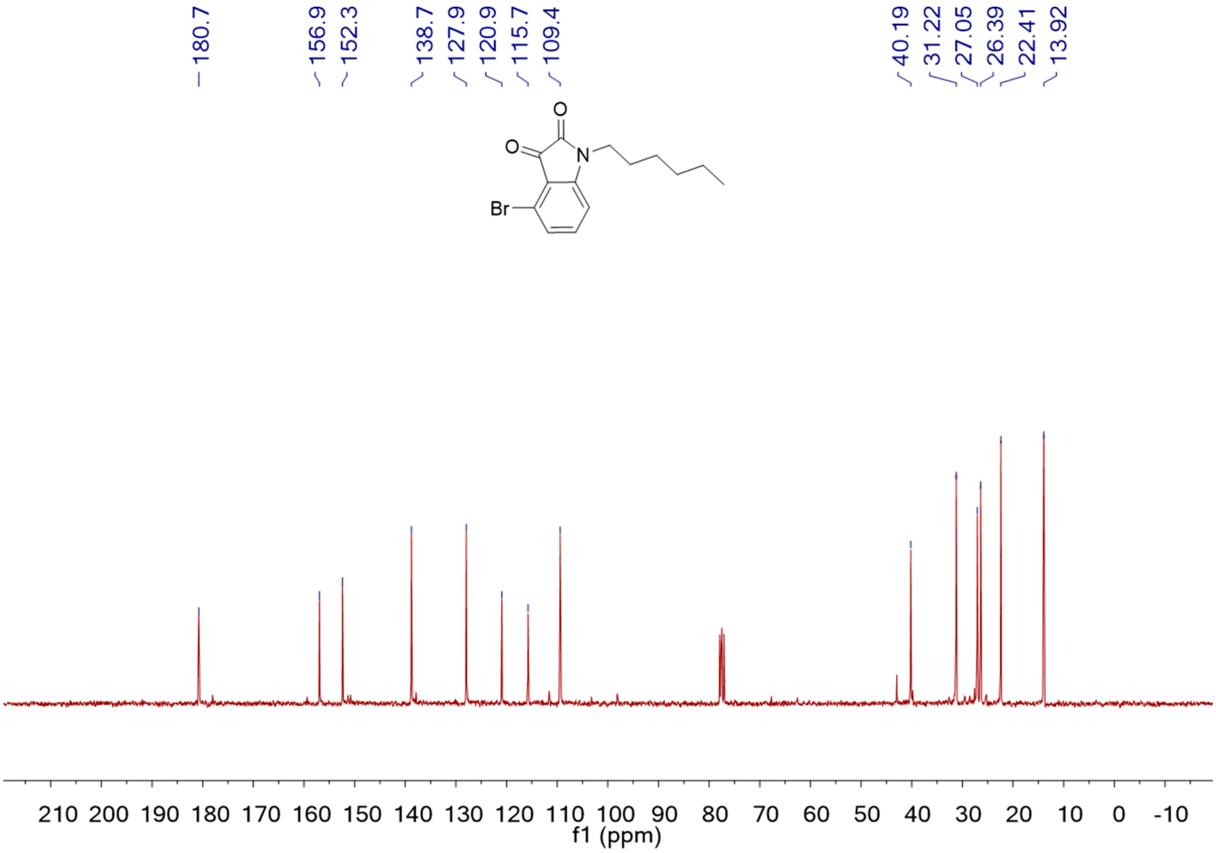
180.7
156.9
152.3
138.7
127.9
120.9
115.7
109.4
40.19
31.22
27.05
26.39
22.41
13.92
O
N
Br
210 200 190 180 170 160 150 140 130 120 110 100 90 80 70 60 50 40 30 20 10 0 -10
f1 (ppm)

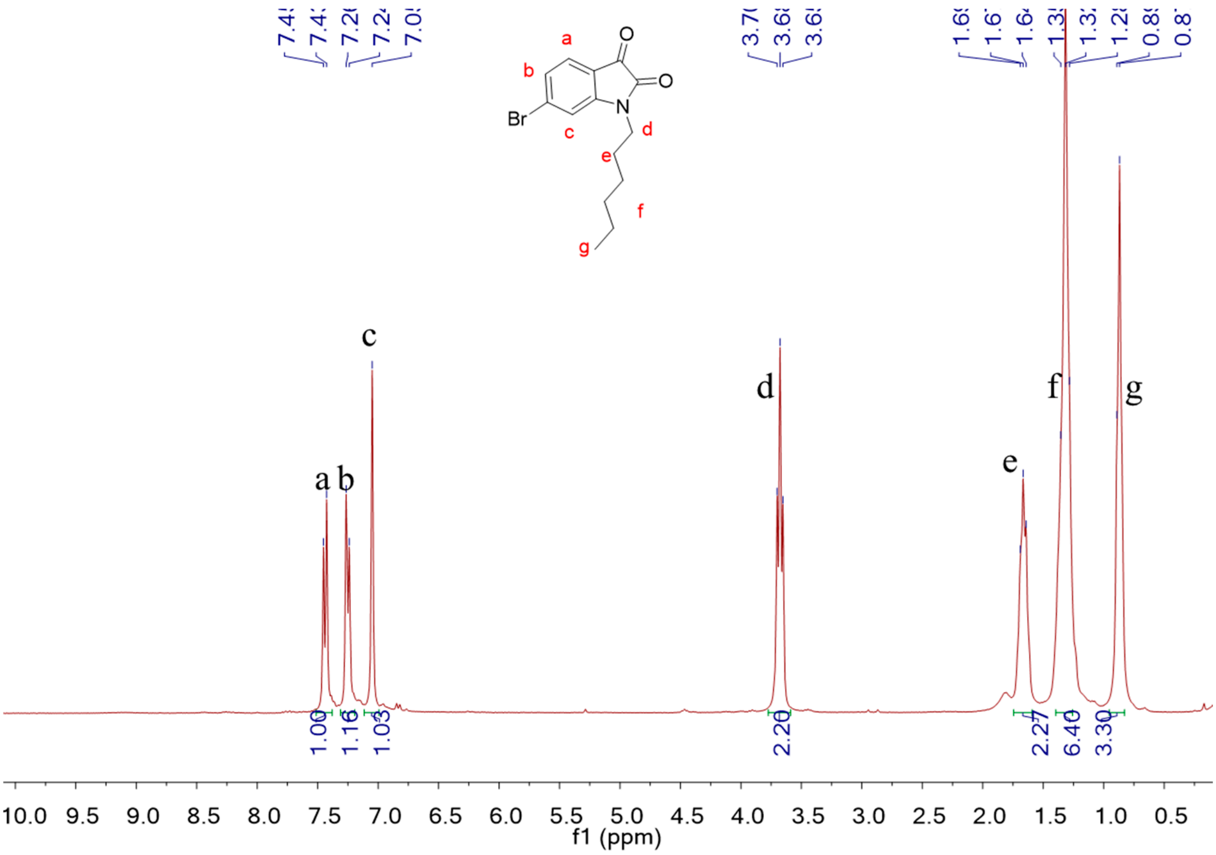
Br
N
O
a
b
c
d
e
f
g
1.00
1.16
1.03
2.20
2.27
6.40
3.30
10.0 9.5 9.0 8.5 8.0 7.5 7.0 6.5 6.0 5.5 5.0 4.5 4.0 3.5 3.0 2.5 2.0 1.5 1.0 0.5
f1 (ppm)

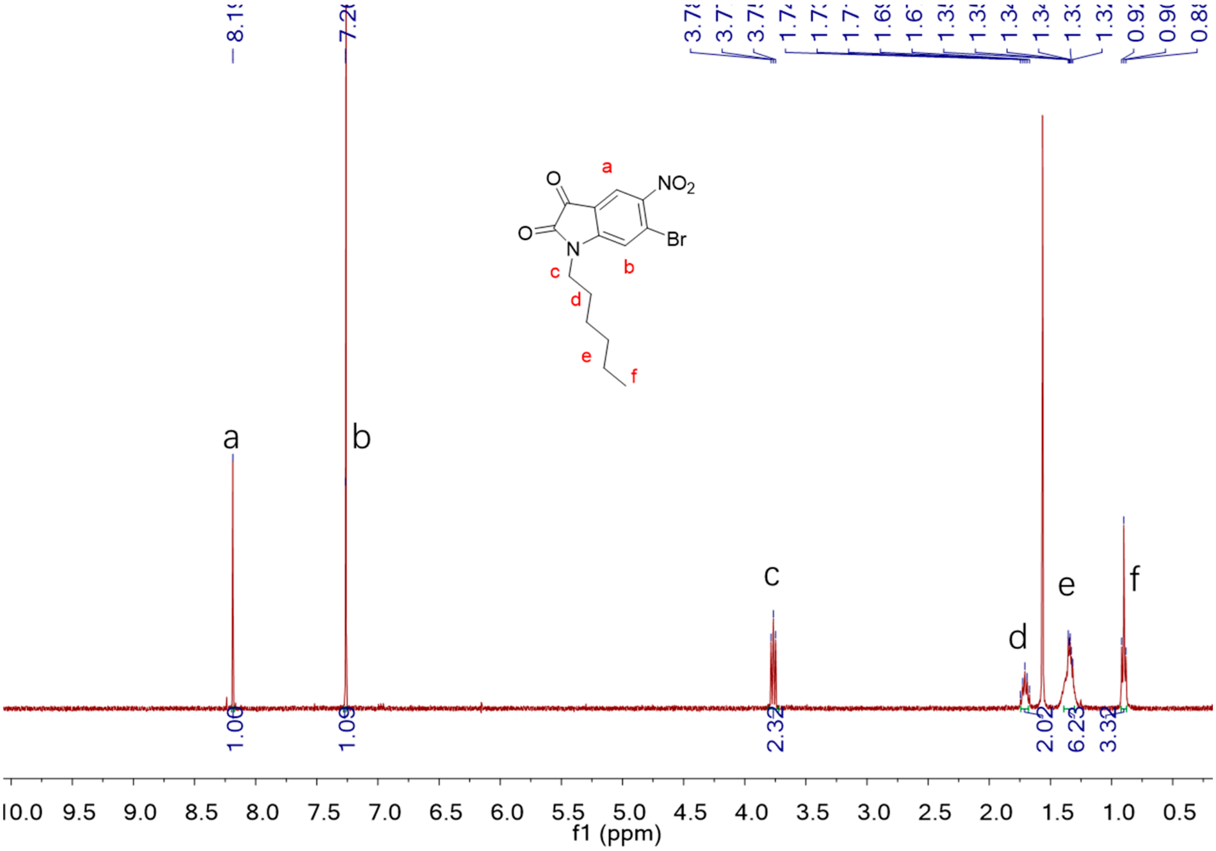
NO2
Br
N
O
a
b
c
d
e
f
1.00
1.09
2.32
2.02
6.23
3.32
10.0 9.5 9.0 8.5 8.0 7.5 7.0 6.5 6.0 5.5 5.0 4.5 4.0 3.5 3.0 2.5 2.0 1.5 1.0 0.5
f1 (ppm)

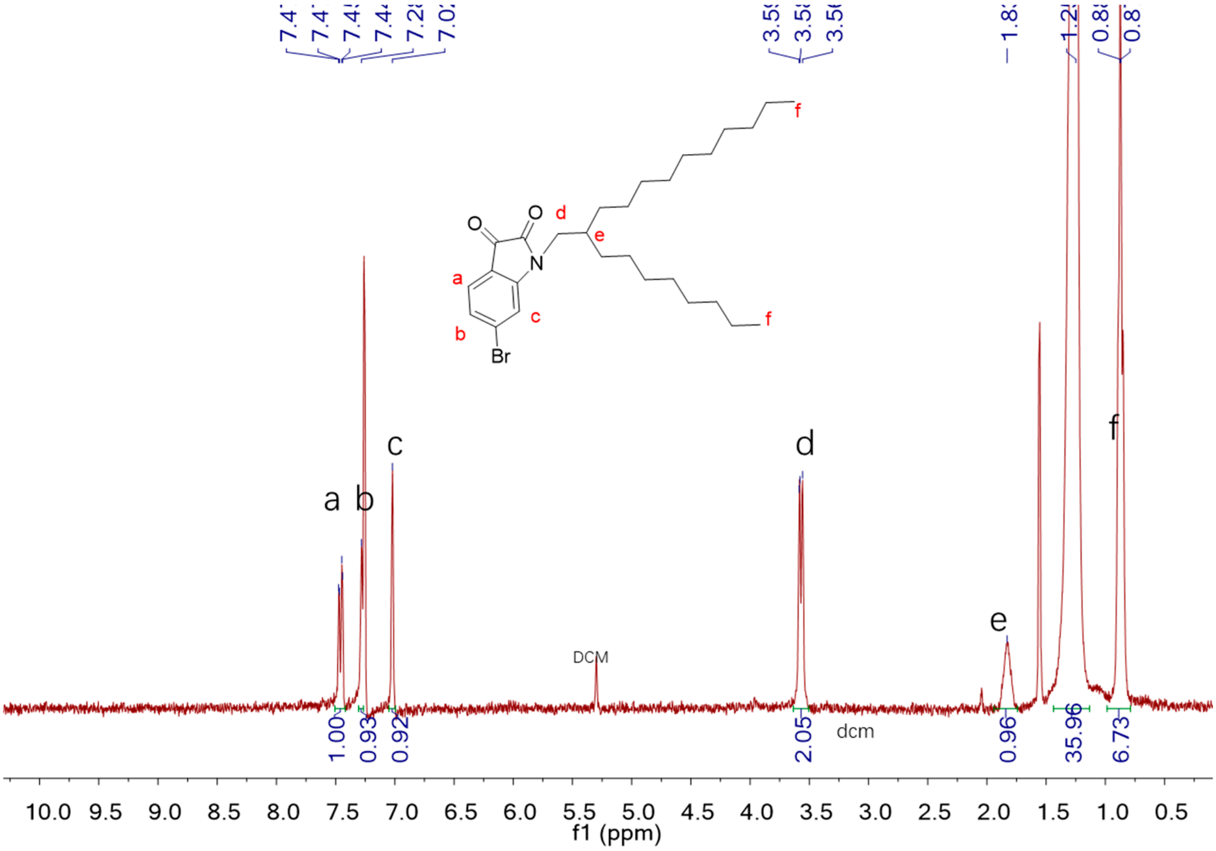

7.47
7.47
7.45
7.44
7.28
7.02
3.59
3.58
3.56
1.83
1.25
0.88
0.87
O
O
N
Br
a
b
c
d
e
f
f
a b
c
d
e
f
DCM
dcm
1.00
0.93
0.92
2.05
0.96
35.96
6.73
10.0 9.5 9.0 8.5 8.0 7.5 7.0 6.5 6.0 5.5 5.0 4.5 4.0 3.5 3.0 2.5 2.0 1.5 1.0 0.5
f1 (ppm)

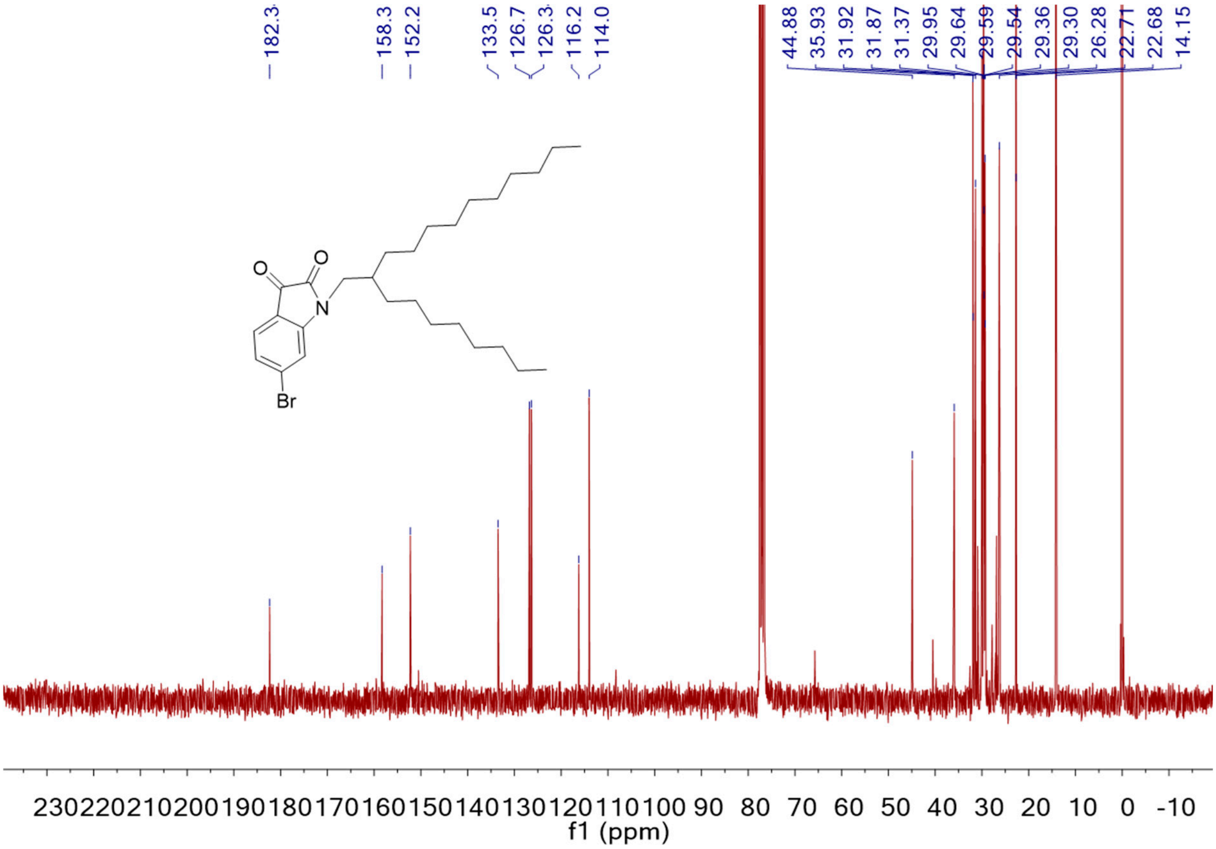

182.3
158.3
152.2
133.5
126.7
126.3
116.2
114.0
44.88
35.93
31.92
31.87
31.37
29.95
29.64
29.59
29.54
29.36
29.30
26.28
22.71
22.68
14.15
O
O
N
Br
230 220 210 200 190 180 170 160 150 140 130 120 110 100 90 80 70 60 50 40 30 20 10 0 -10
f1 (ppm)

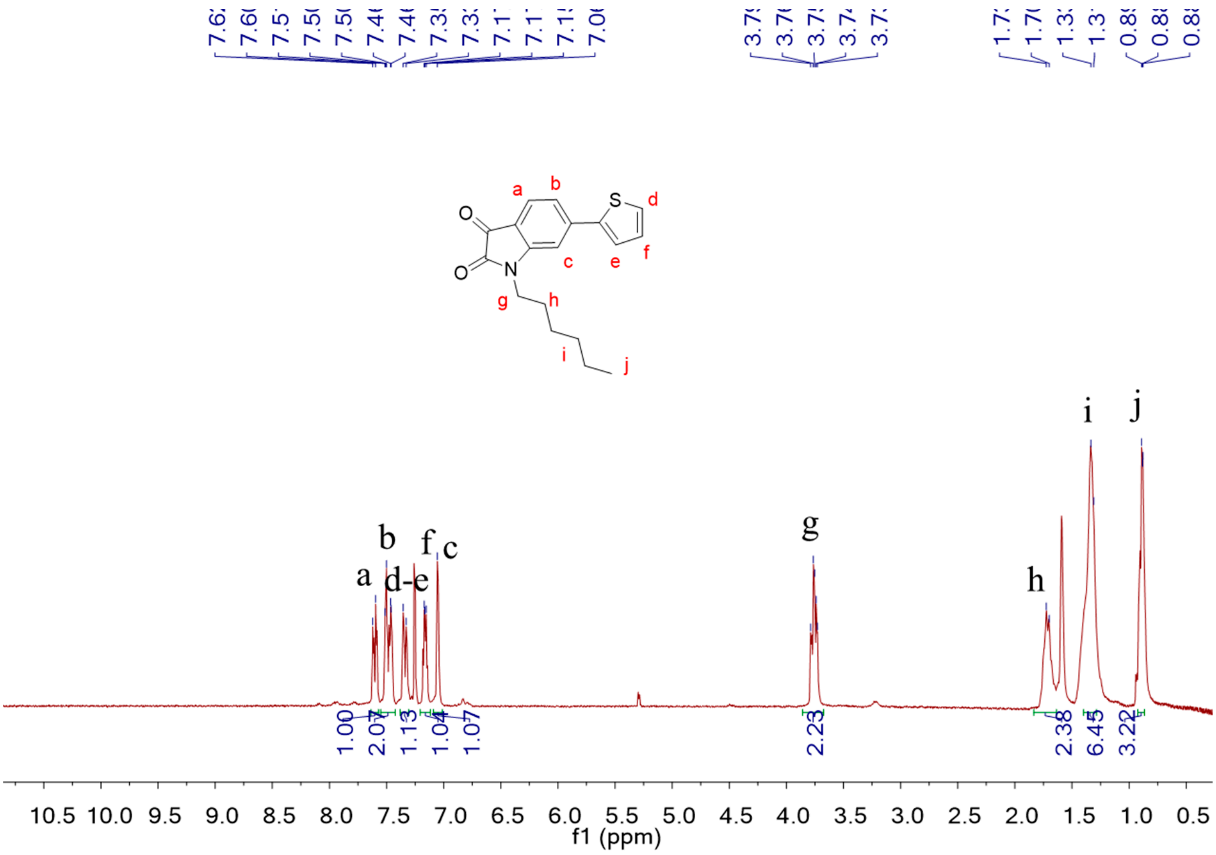

7.62
7.60
7.51
7.50
7.50
7.40
7.40
7.35
7.33
7.17
7.17
7.15
7.06
3.79
3.76
3.75
3.74
3.72
1.73
1.70
1.33
1.31
0.89
0.88
0.88
a
b
S
d
O
O
N
c
e
f
g
h
i
j
a
b
d-e
f
c
g
h
i
j
1.00
2.07
1.13
1.04
1.07
2.23
2.38
6.45
3.22
10.5 10.0 9.5 9.0 8.5 8.0 7.5 7.0 6.5 6.0 5.5 5.0 4.5 4.0 3.5 3.0 2.5 2.0 1.5 1.0 0.5
f1 (ppm)

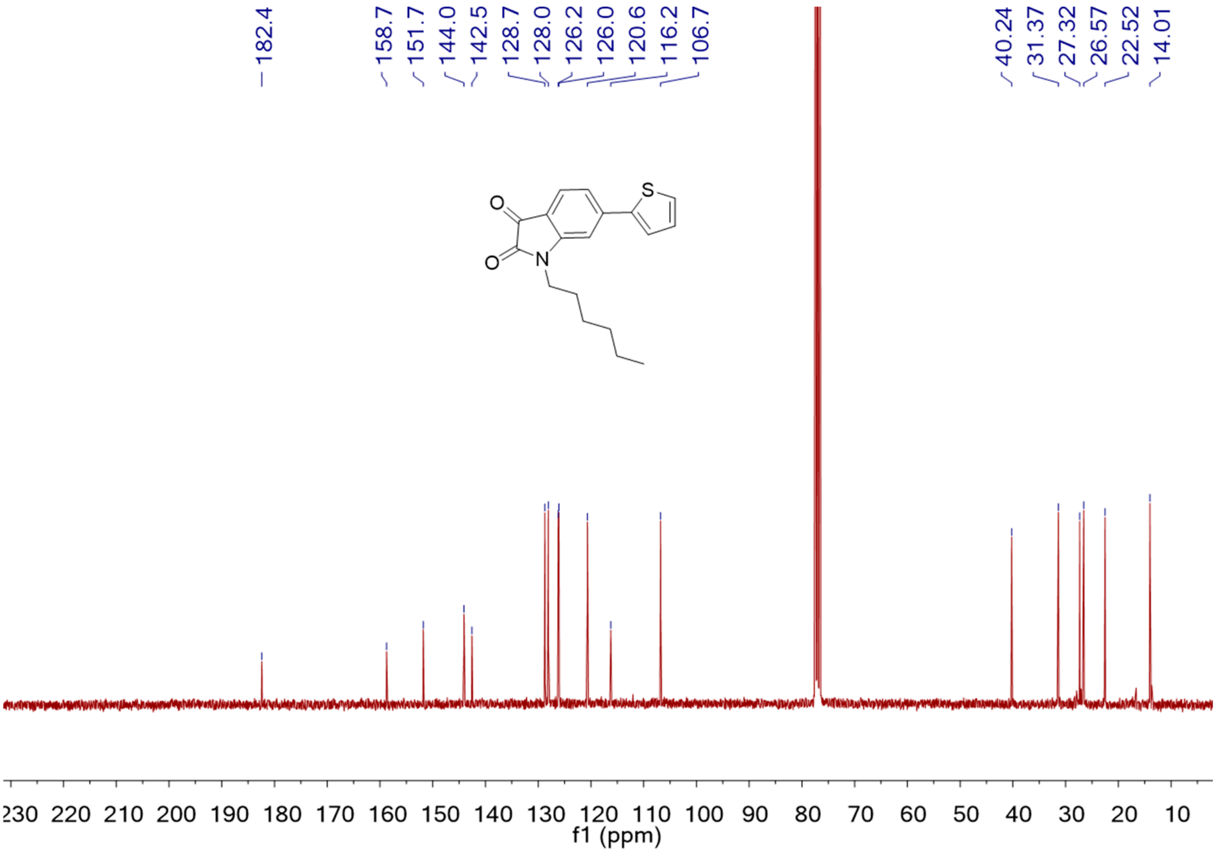

182.4
158.7
151.7
144.0
142.5
128.7
128.0
126.2
126.0
120.6
116.2
106.7
40.24
31.37
27.32
26.57
22.52
14.01
S
O
O
N
230 220 210 200 190 180 170 160 150 140 130 120 110 100 90 80 70 60 50 40 30 20 10
f1 (ppm)

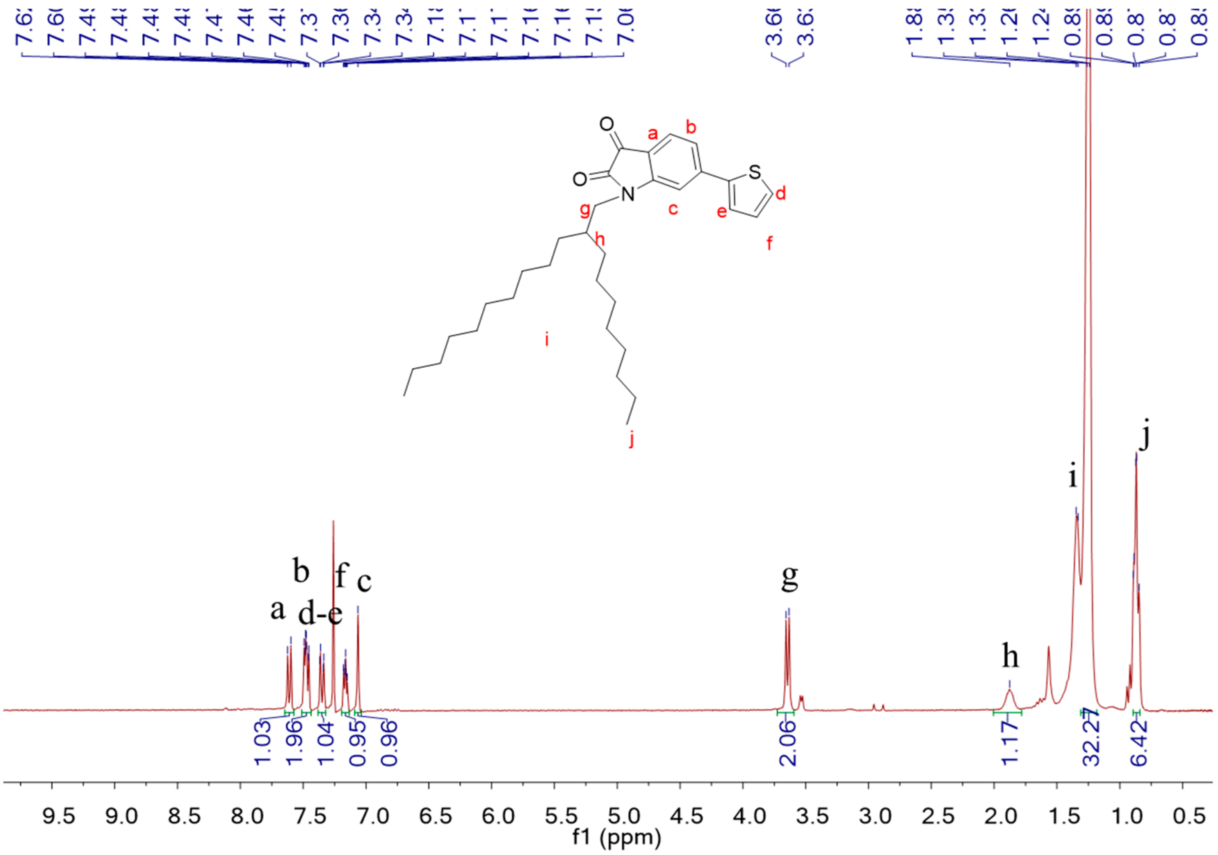
3.66
3.63
a
b
d-e
f
c
g
h
i
j
1.03
1.96
1.04
0.95
0.96
2.06
1.17
32.27
6.42
9.5 9.0 8.5 8.0 7.5 7.0 6.5 6.0 5.5 5.0 4.5 4.0 3.5 3.0 2.5 2.0 1.5 1.0 0.5
f1 (ppm)

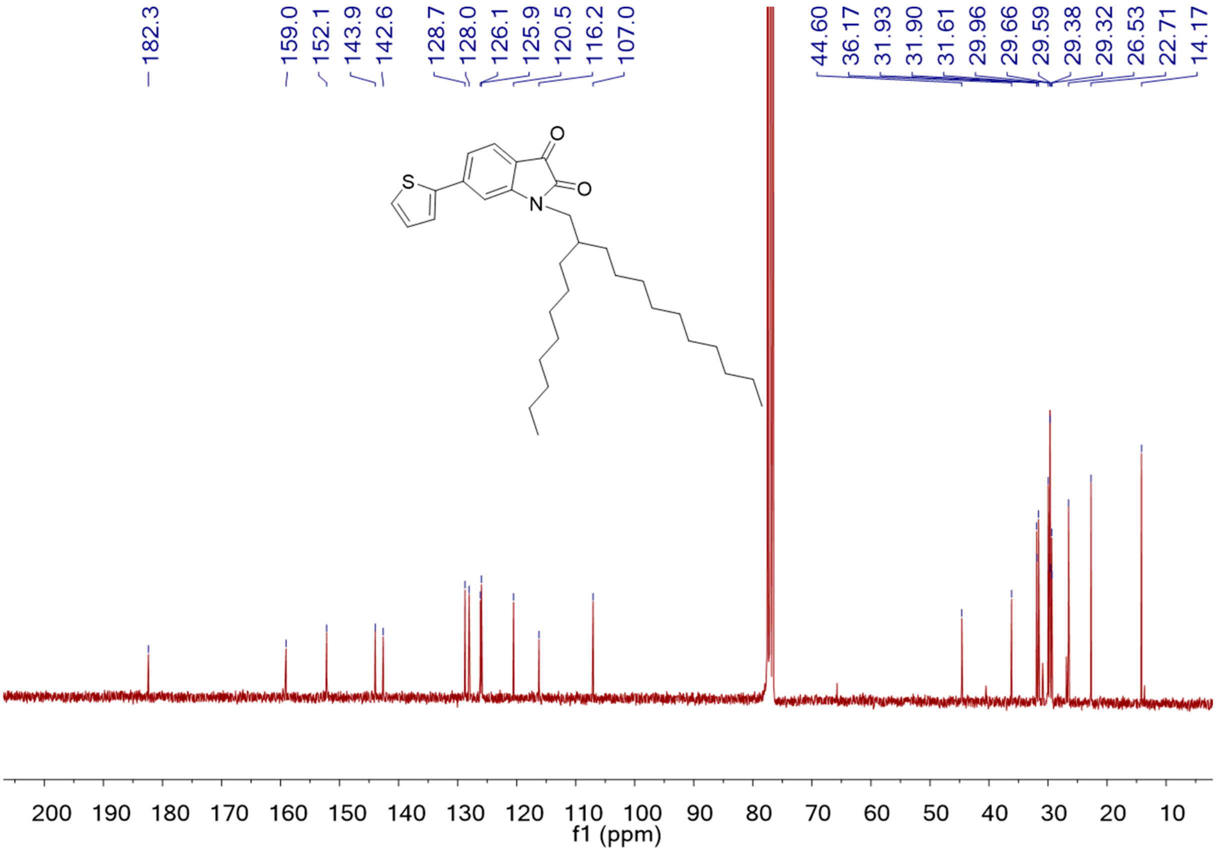
182.3
159.0
152.1
143.9
142.6
128.7
128.0
126.1
125.9
120.5
116.2
107.0
44.60
36.17
31.93
31.90
31.61
29.96
29.66
29.59
29.38
29.32
26.53
22.71
14.17
200 190 180 170 160 150 140 130 120 110 100 90 80 70 60 50 40 30 20 10
f1 (ppm)

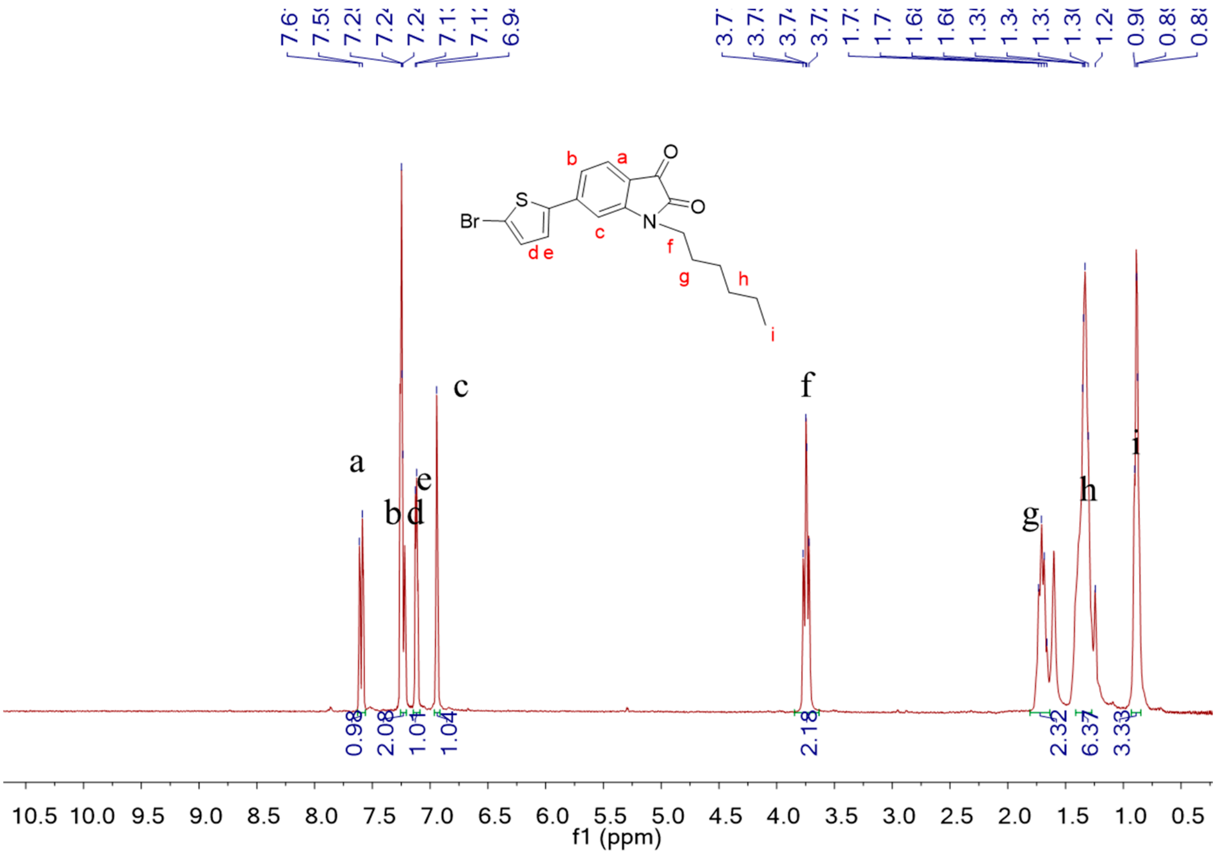
Br
S
N
O
O
a
b
c
d e
f
g
h
i
0.98
2.08
1.04
1.04
2.18
2.32
6.37
3.33
10.5 10.0 9.5 9.0 8.5 8.0 7.5 7.0 6.5 6.0 5.5 5.0 4.5 4.0 3.5 3.0 2.5 2.0 1.5 1.0 0.5
f1 (ppm)

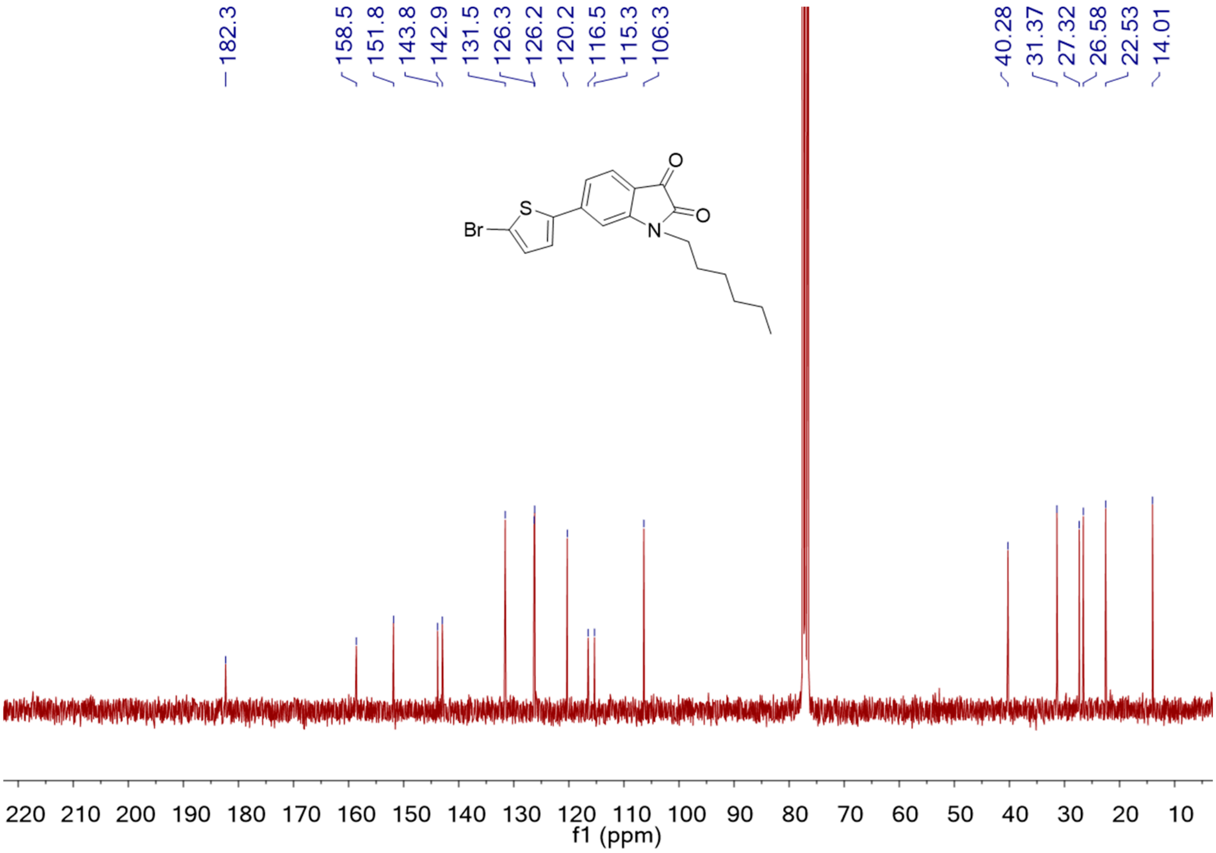
182.3
158.5
151.8
143.8
142.9
131.5
126.3
126.2
120.2
116.5
115.3
106.3
40.28
31.37
27.32
26.58
22.53
14.01
Br
S
N
O
O
220 210 200 190 180 170 160 150 140 130 120 110 100 90 80 70 60 50 40 30 20 10
f1 (ppm)

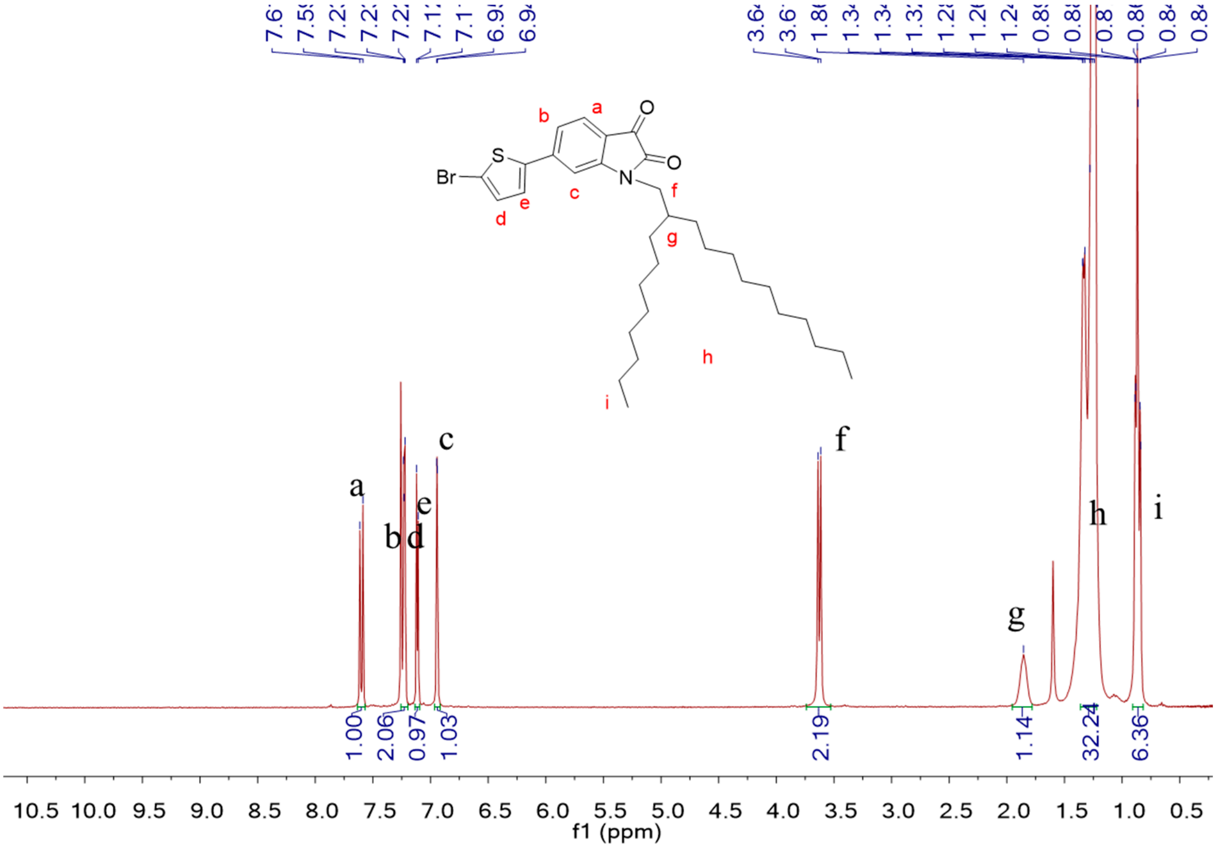
a
b
c
d
e
f
g
h
i
Br
S
N
O
1.00
2.06
0.97
1.03
2.19
1.14
32.24
6.36
10.5 10.0 9.5 9.0 8.5 8.0 7.5 7.0 6.5 6.0 5.5 5.0 4.5 4.0 3.5 3.0 2.5 2.0 1.5 1.0 0.5
f1 (ppm)

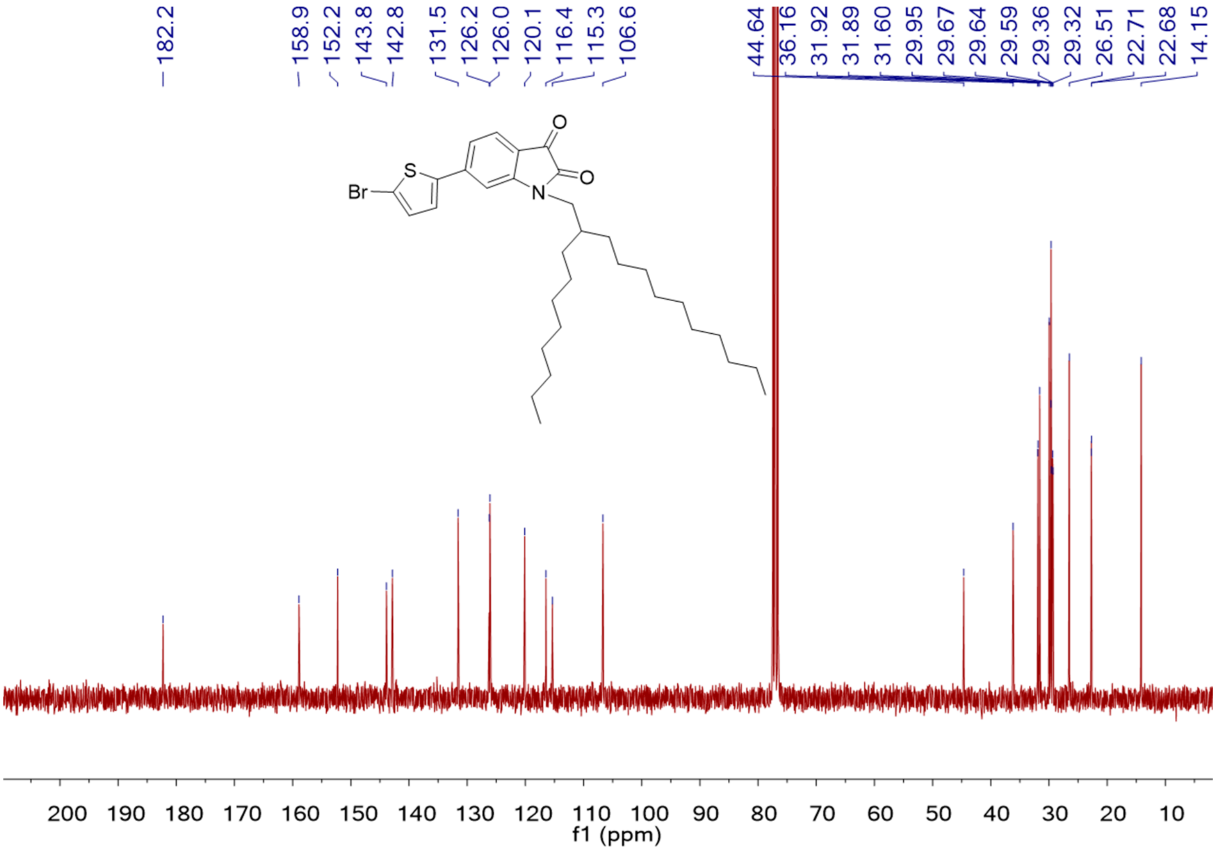
182.2
158.9
152.2
143.8
142.8
131.5
126.2
126.0
120.1
116.4
115.3
106.6
44.64
36.16
31.92
31.89
31.60
29.95
29.67
29.64
29.59
29.36
29.32
26.51
22.71
22.68
14.15
Br
S
N
O
200 190 180 170 160 150 140 130 120 110 100 90 80 70 60 50 40 30 20 10
f1 (ppm)

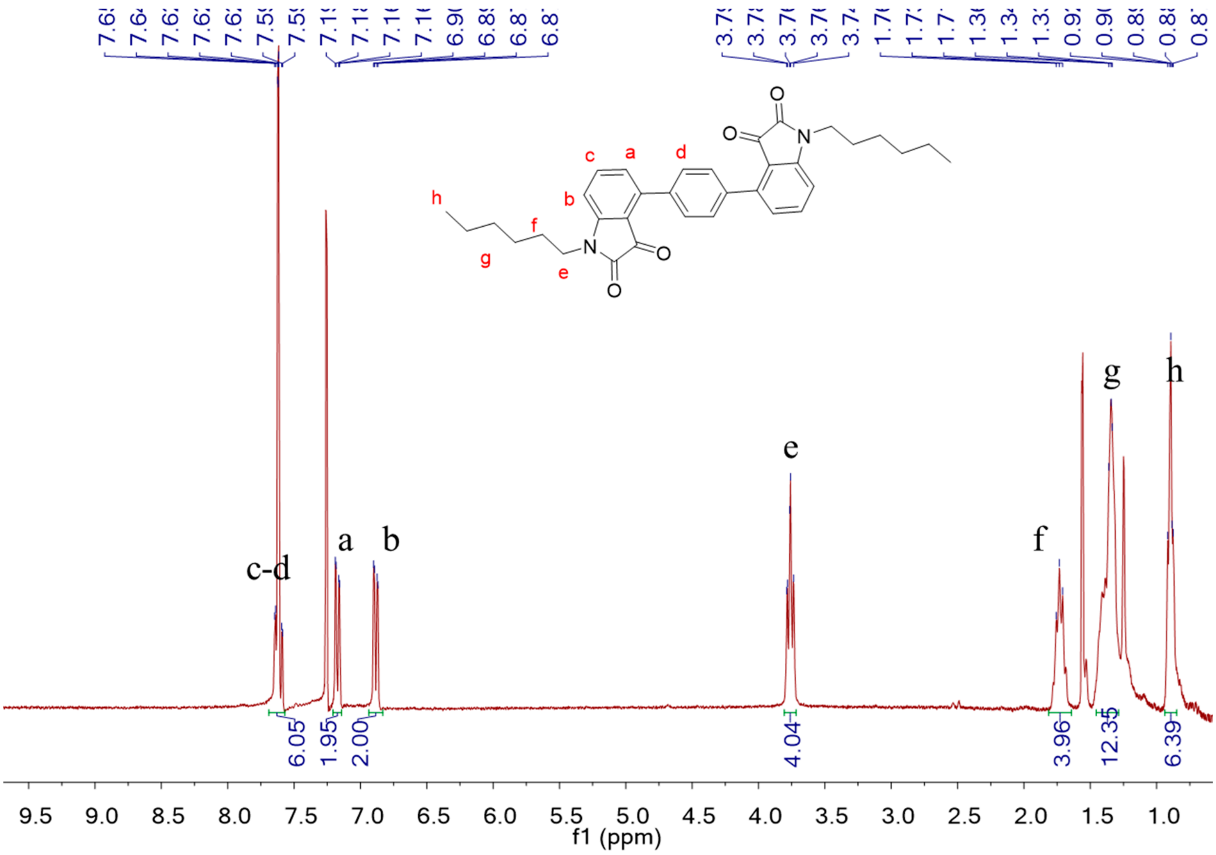
c-d
a
b
e
f
g
h
6.05
1.95
2.00
4.04
3.96
12.35
6.39
9.5 9.0 8.5 8.0 7.5 7.0 6.5 6.0 5.5 5.0 4.5 4.0 3.5 3.0 2.5 2.0 1.5 1.0
f1 (ppm)

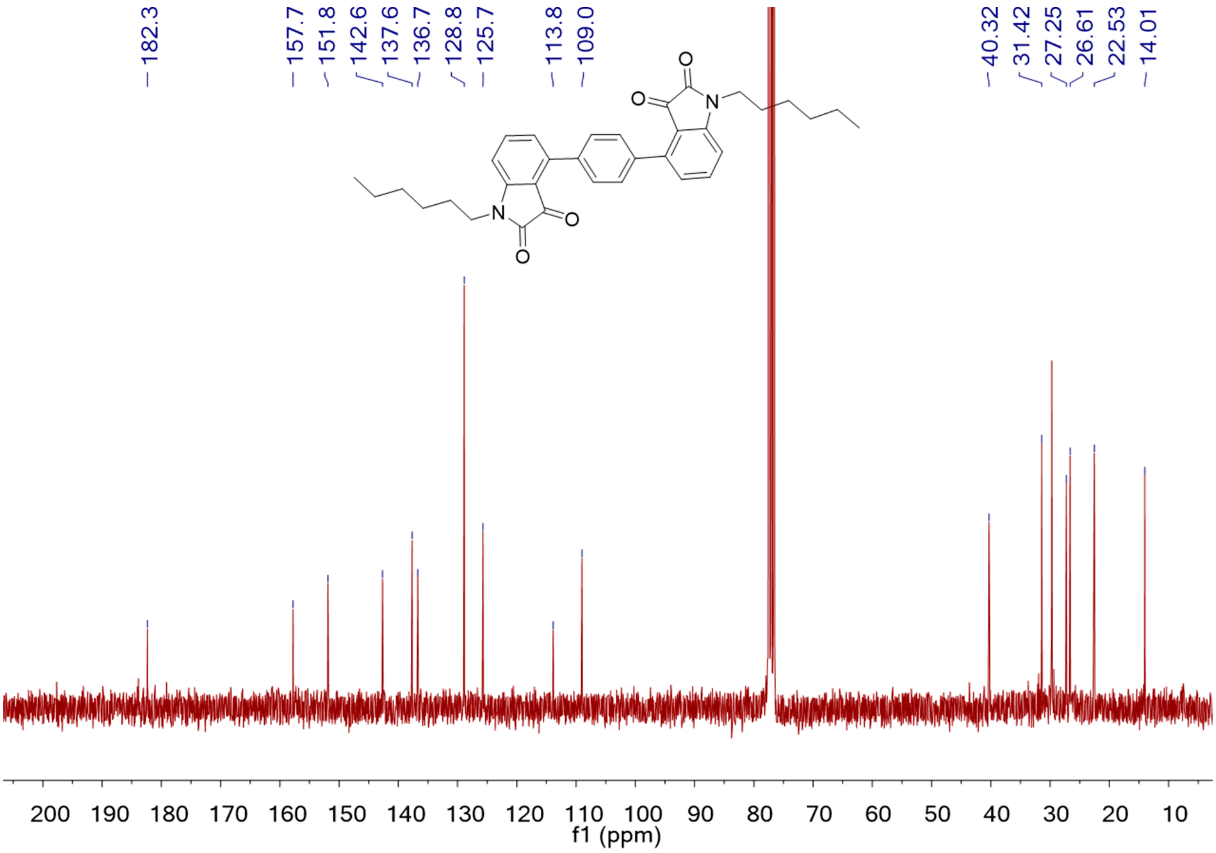
182.3
157.7
151.8
142.6
137.6
136.7
128.8
125.7
113.8
109.0
40.32
31.42
27.25
26.61
22.53
14.01
200 190 180 170 160 150 140 130 120 110 100 90 80 70 60 50 40 30 20 10
f1 (ppm)

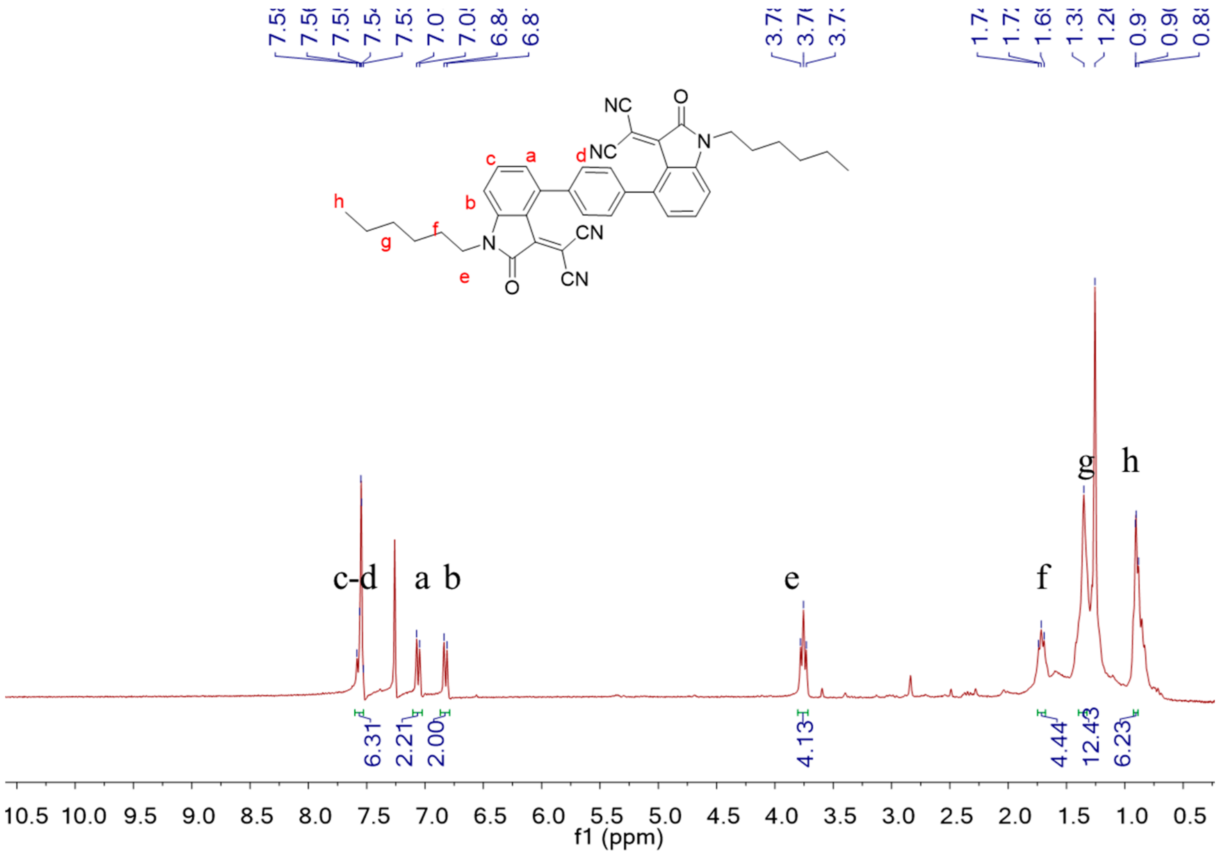
NC
O
NC
N
c
a
d
b
h
g
f
N
e
O
CN
CN
c-d
a
b
e
f
g
h
6.31
2.21
2.00
4.13
4.44
12.43
6.23
f1 (ppm)

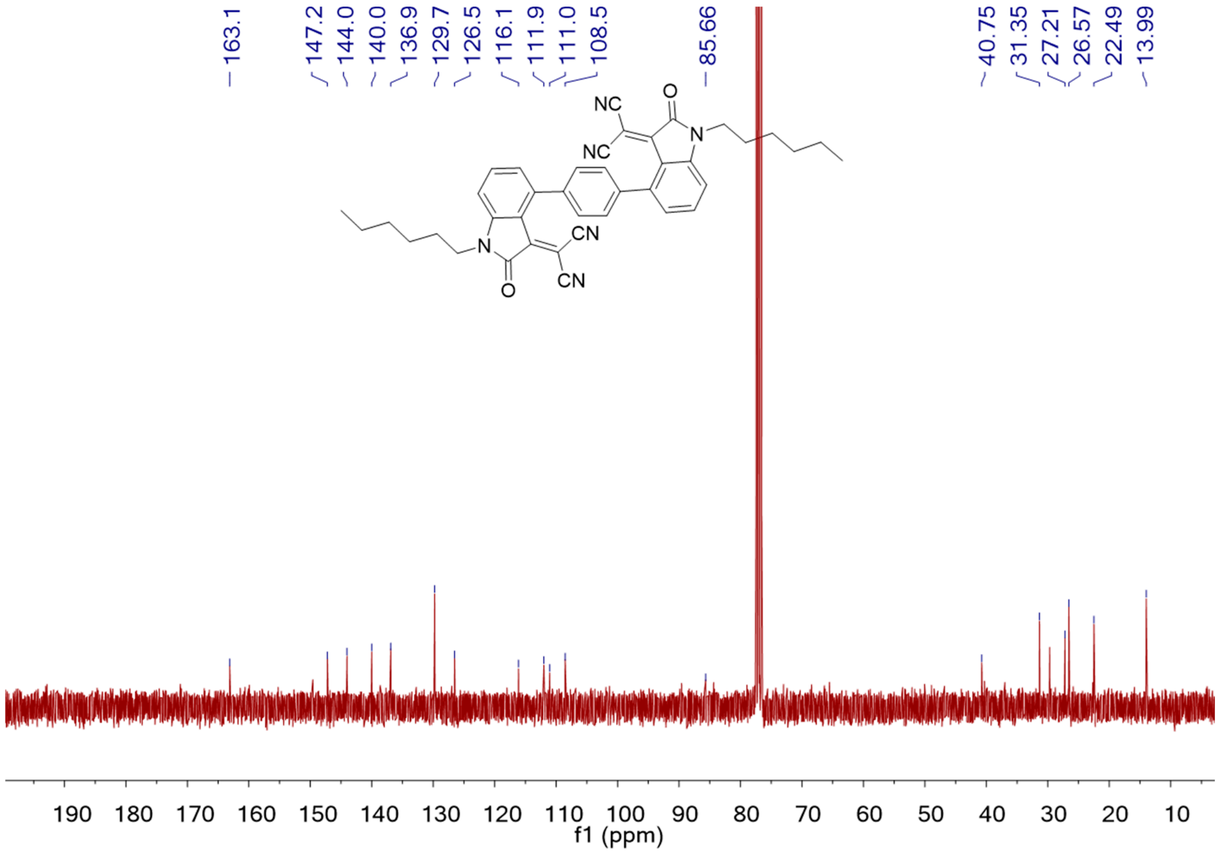
163.1
147.2
144.0
140.0
136.9
129.7
126.5
116.1
111.9
111.0
108.5
85.66
40.75
31.35
27.21
26.57
22.49
13.99
NC
O
NC
N
N
CN
O
CN
f1 (ppm)

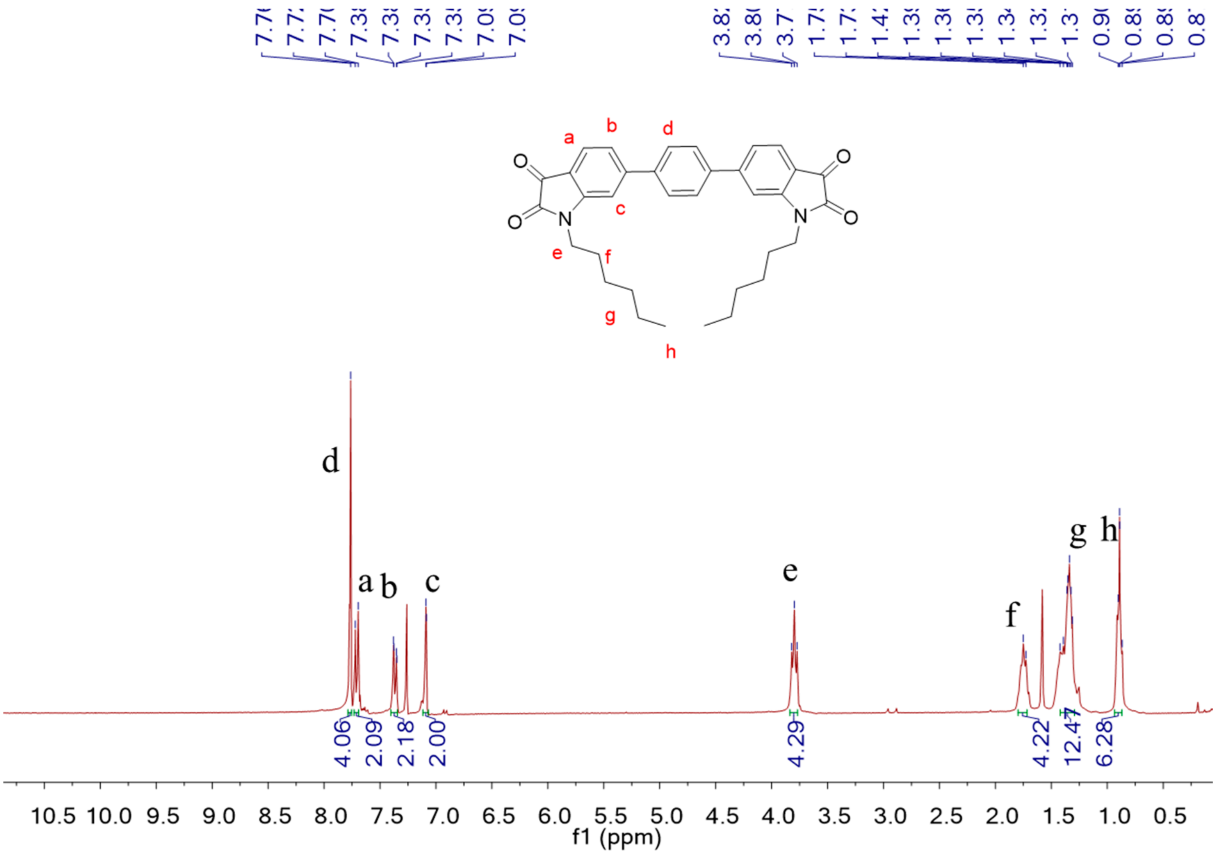
a
b
d
c
e
f
g
h
O
N
d
a
b
c
e
f
g
h
4.06
2.09
2.18
2.00
4.29
4.22
12.47
6.28
10.5 10.0 9.5 9.0 8.5 8.0 7.5 7.0 6.5 6.0 5.5 5.0 4.5 4.0 3.5 3.0 2.5 2.0 1.5 1.0 0.5
f1 (ppm)

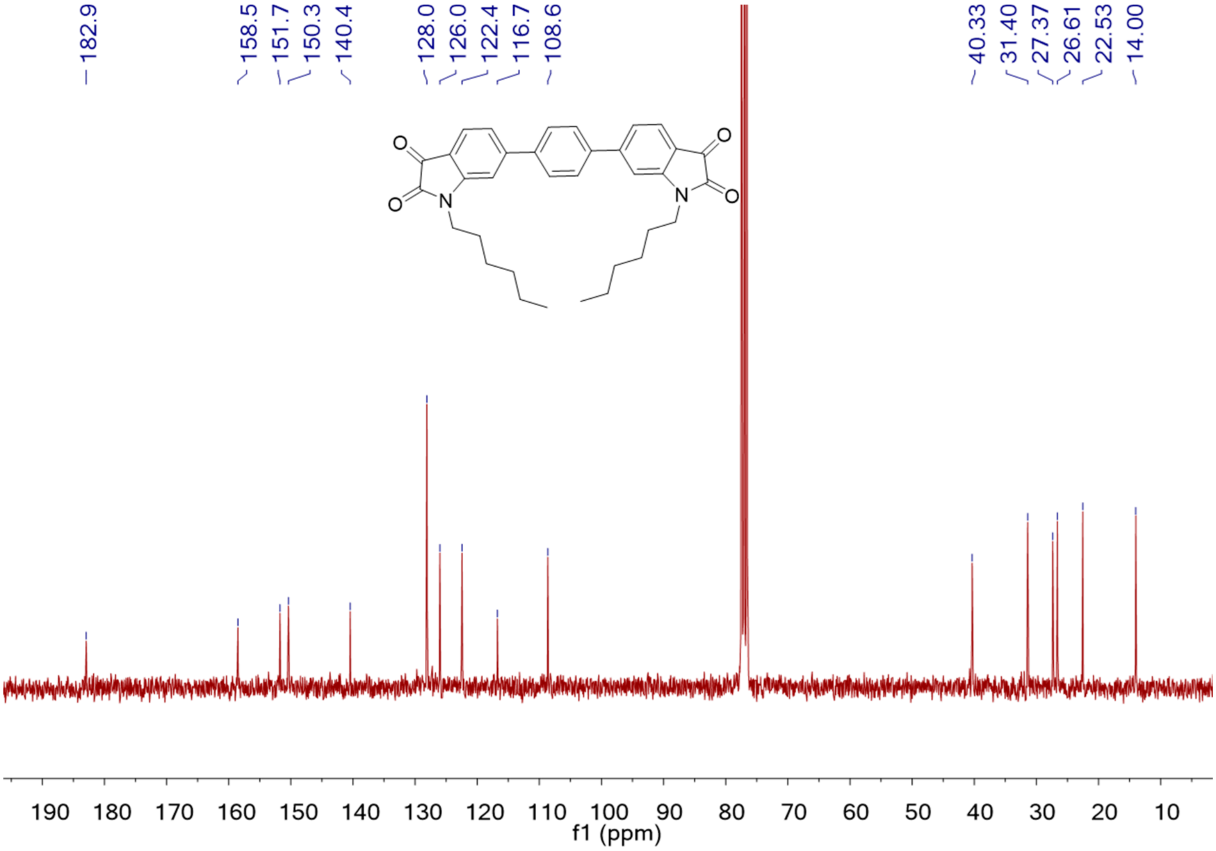
182.9
158.5
151.7
150.3
140.4
128.0
126.0
122.4
116.7
108.6
40.33
31.40
27.37
26.61
22.53
14.00
O
N
190 180 170 160 150 140 130 120 110 100 90 80 70 60 50 40 30 20 10
f1 (ppm)

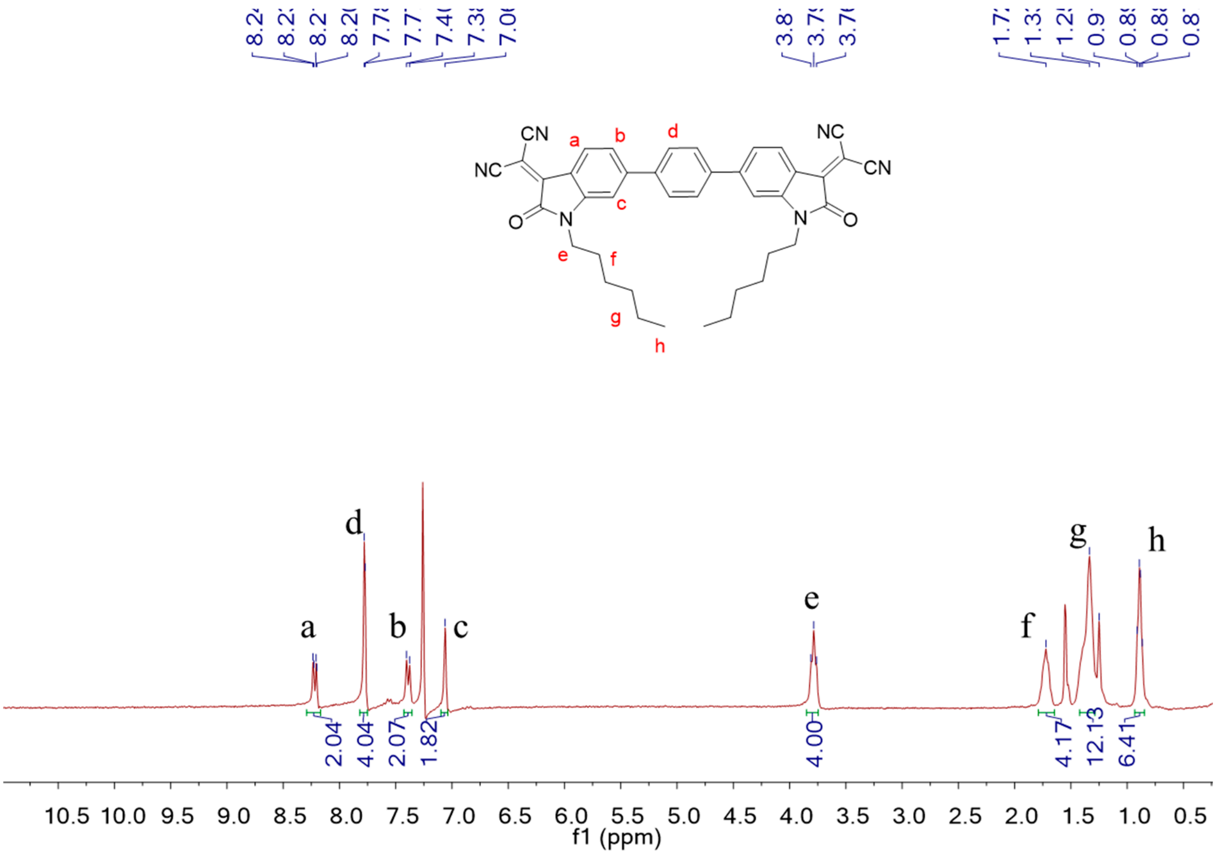
CN
NC
a
b
c
d
e
f
g
h
N
O
2.04
4.04
2.07
1.82
4.00
4.17
12.13
6.41
10.5 10.0 9.5 9.0 8.5 8.0 7.5 7.0 6.5 6.0 5.5 5.0 4.5 4.0 3.5 3.0 2.5 2.0 1.5 1.0 0.5
f1 (ppm)

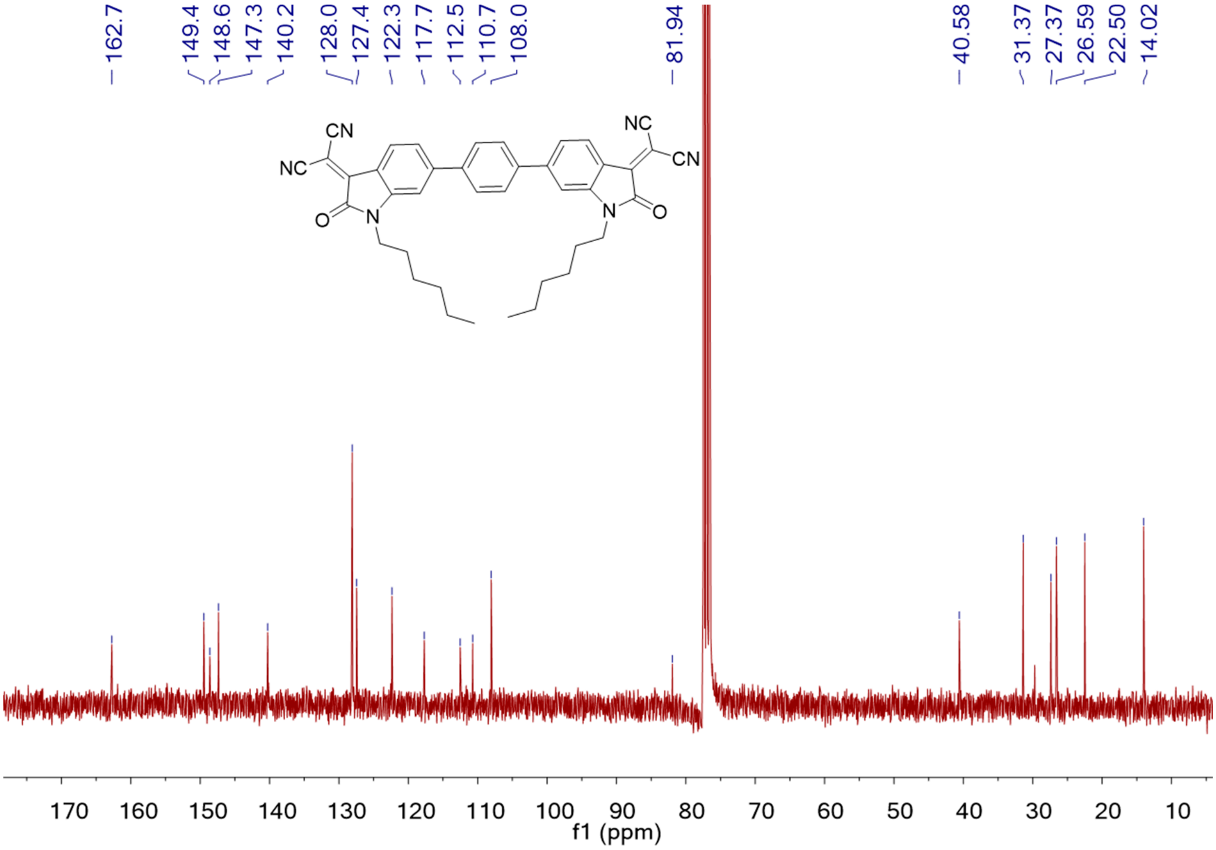
162.7
149.4
148.6
147.3
140.2
128.0
127.4
122.3
117.7
112.5
110.7
108.0
81.94
40.58
31.37
27.37
26.59
22.50
14.02
CN
NC
N
O
170 160 150 140 130 120 110 100 90 80 70 60 50 40 30 20 10
f1 (ppm)

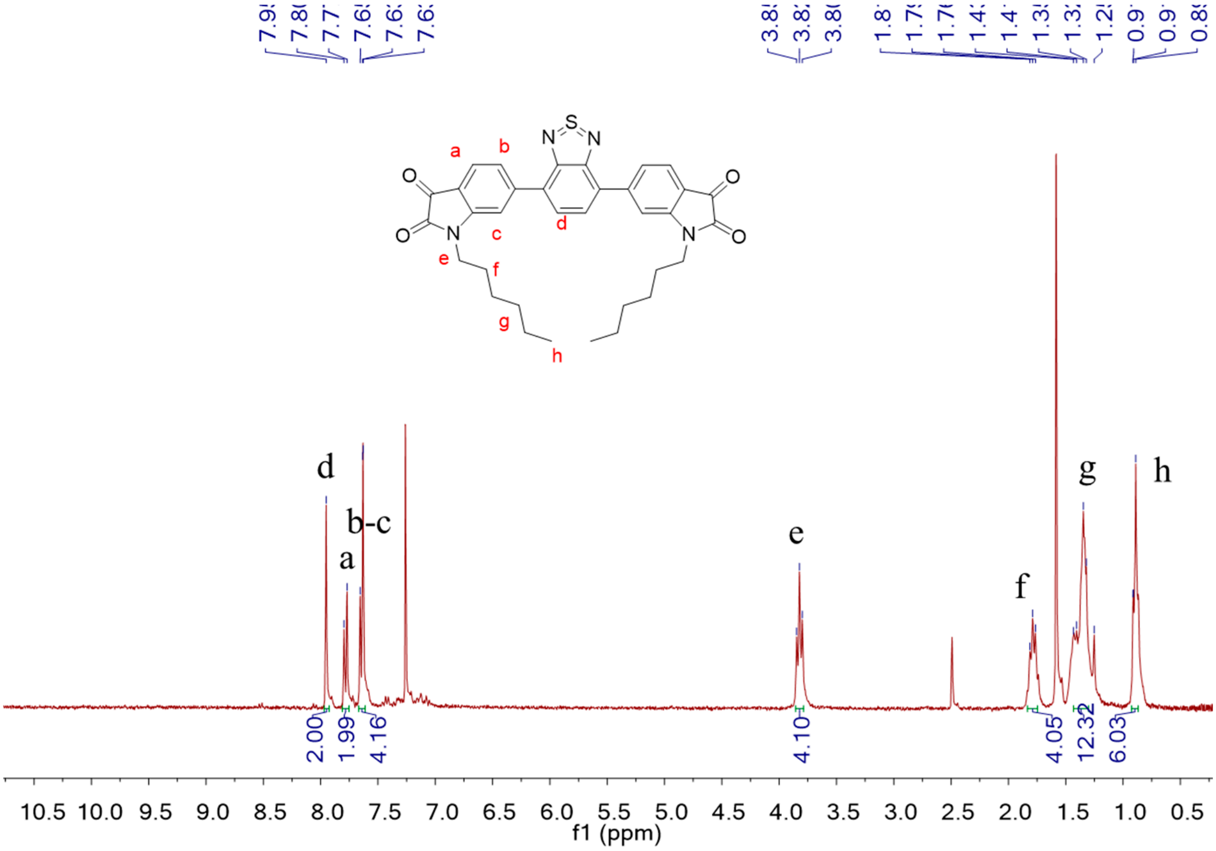
a
b
c
d
e
f
g
h
N
S
O
2.00
1.99
4.16
4.10
4.05
12.32
6.03
10.5 10.0 9.5 9.0 8.5 8.0 7.5 7.0 6.5 6.0 5.5 5.0 4.5 4.0 3.5 3.0 2.5 2.0 1.5 1.0 0.5
f1 (ppm)

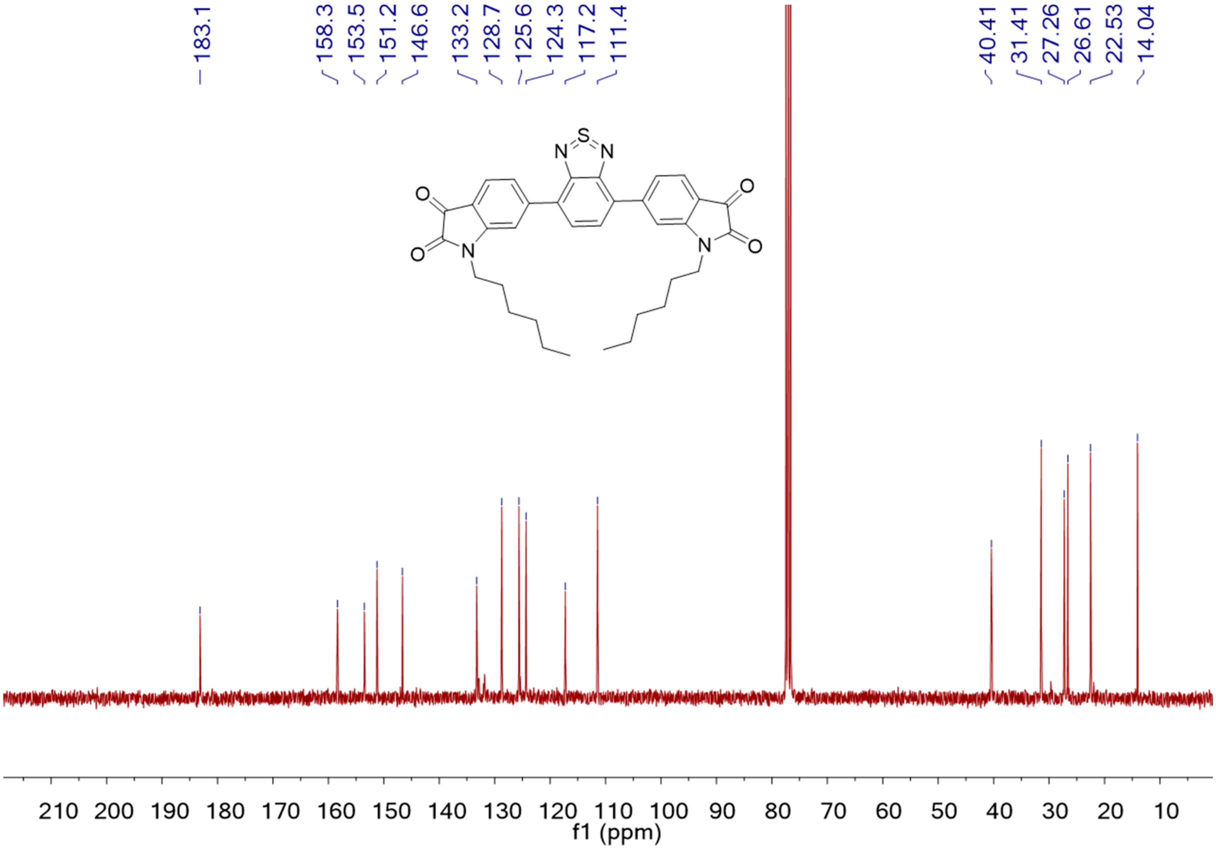
183.1
158.3
153.5
151.2
146.6
133.2
128.7
125.6
124.3
117.2
111.4
40.41
31.41
27.26
26.61
22.53
14.04
N
S
O
210 200 190 180 170 160 150 140 130 120 110 100 90 80 70 60 50 40 30 20 10
f1 (ppm)

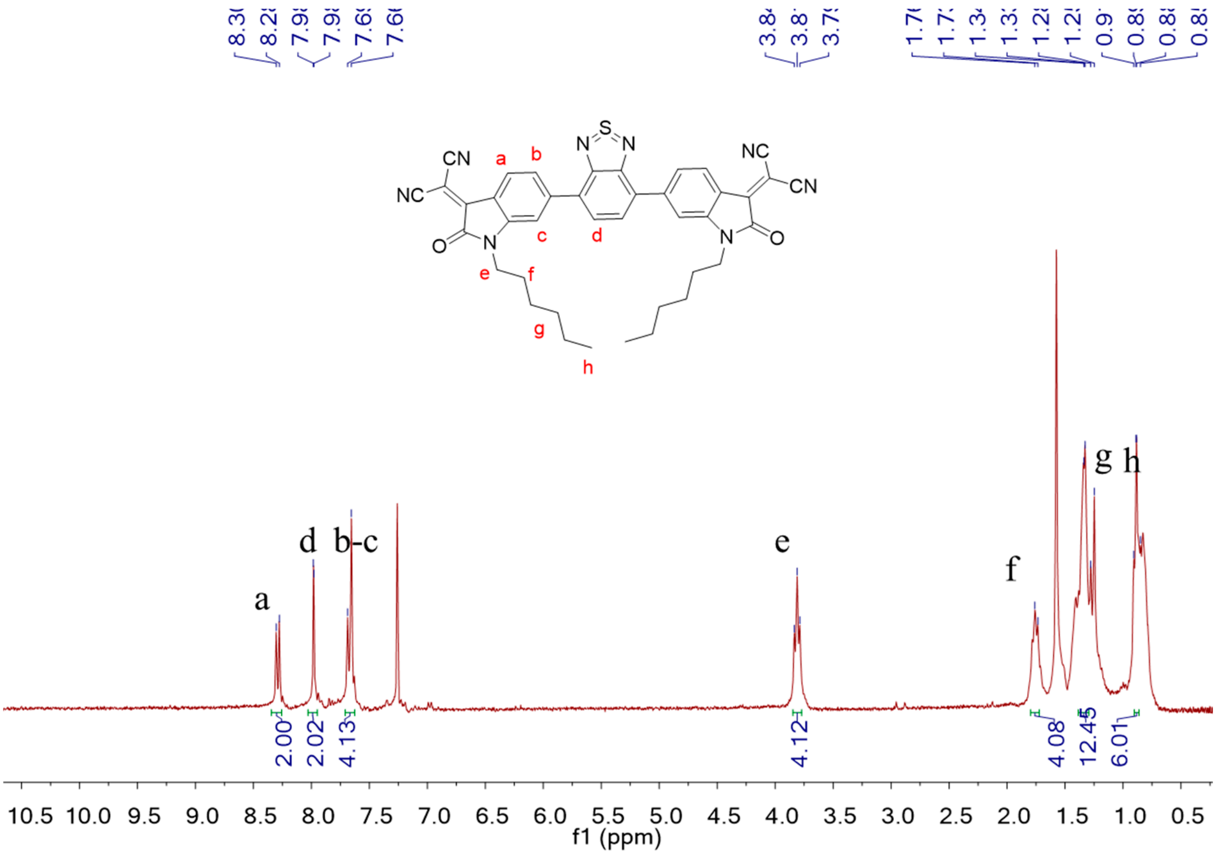
8.30
8.28
7.98
7.98
7.69
7.66
3.84
3.81
3.79
1.76
1.73
1.34
1.33
1.28
1.25
0.91
0.89
0.88
0.85
CN
NC
a
b
S
N
N
NC
CN
O
N
c
d
N
O
e
f
g
h
a
d
b-c
e
f
g
h
2.00
2.02
4.13
4.12
4.08
12.45
6.01
10.5 10.0 9.5 9.0 8.5 8.0 7.5 7.0 6.5 6.0 5.5 5.0 4.5 4.0 3.5 3.0 2.5 2.0 1.5 1.0 0.5
f1 (ppm)

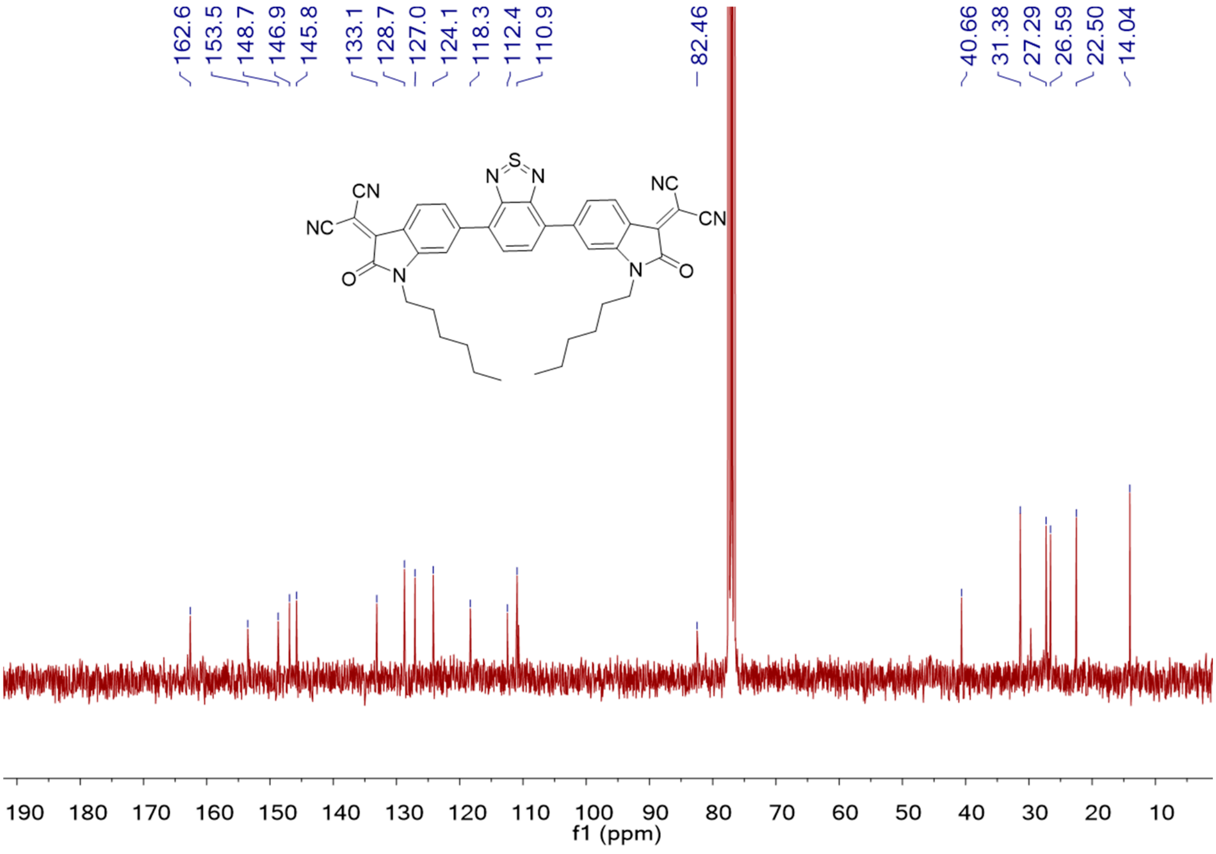
162.6
153.5
148.7
146.9
145.8
133.1
128.7
127.0
124.1
118.3
112.4
110.9
82.46
40.66
31.38
27.29
26.59
22.50
14.04
CN
NC
S
N
N
NC
CN
O
N
N
O
190 180 170 160 150 140 130 120 110 100 90 80 70 60 50 40 30 20 10
f1 (ppm)

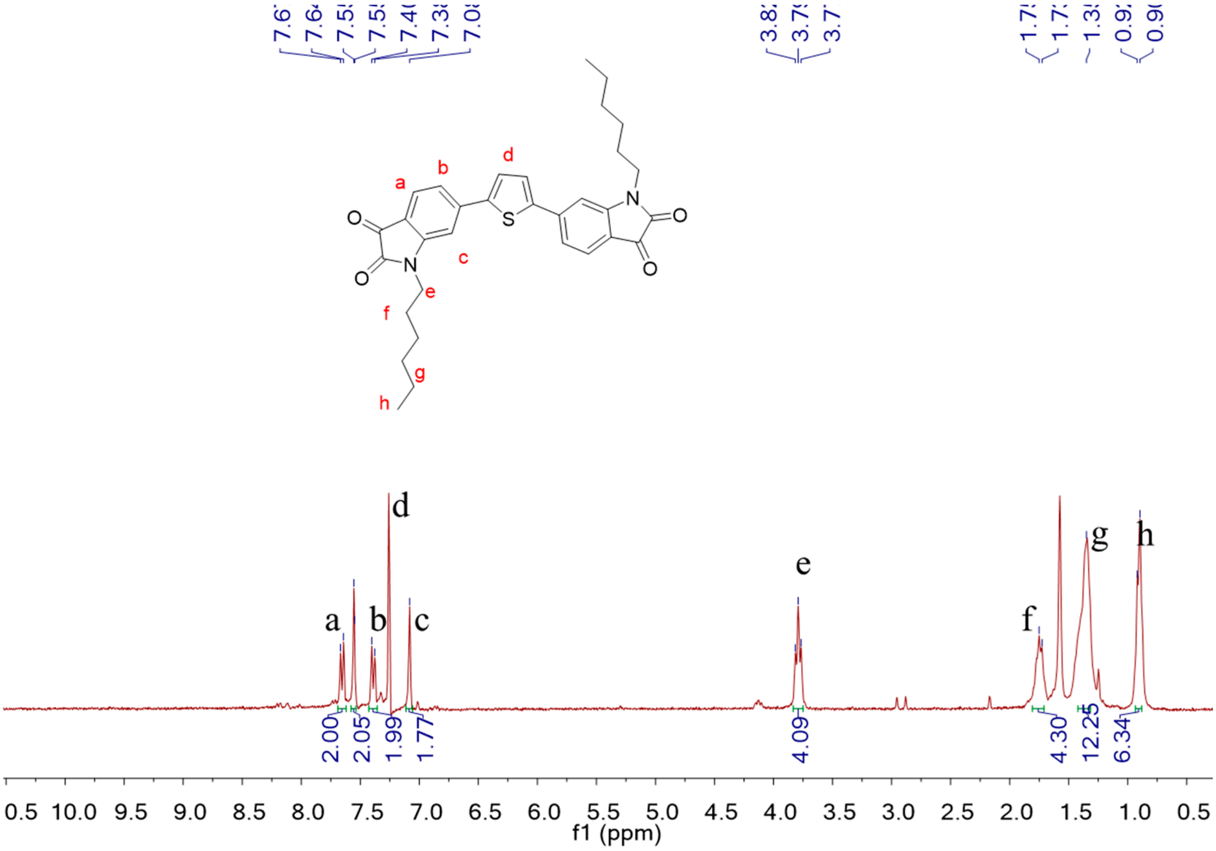

a
b
c
d
e
f
g
h
O
N
S
2.00
2.05
1.99
1.77
4.09
4.30
12.25
6.34
0.5 10.0 9.5 9.0 8.5 8.0 7.5 7.0 6.5 6.0 5.5 5.0 4.5 4.0 3.5 3.0 2.5 2.0 1.5 1.0 0.5
f1 (ppm)

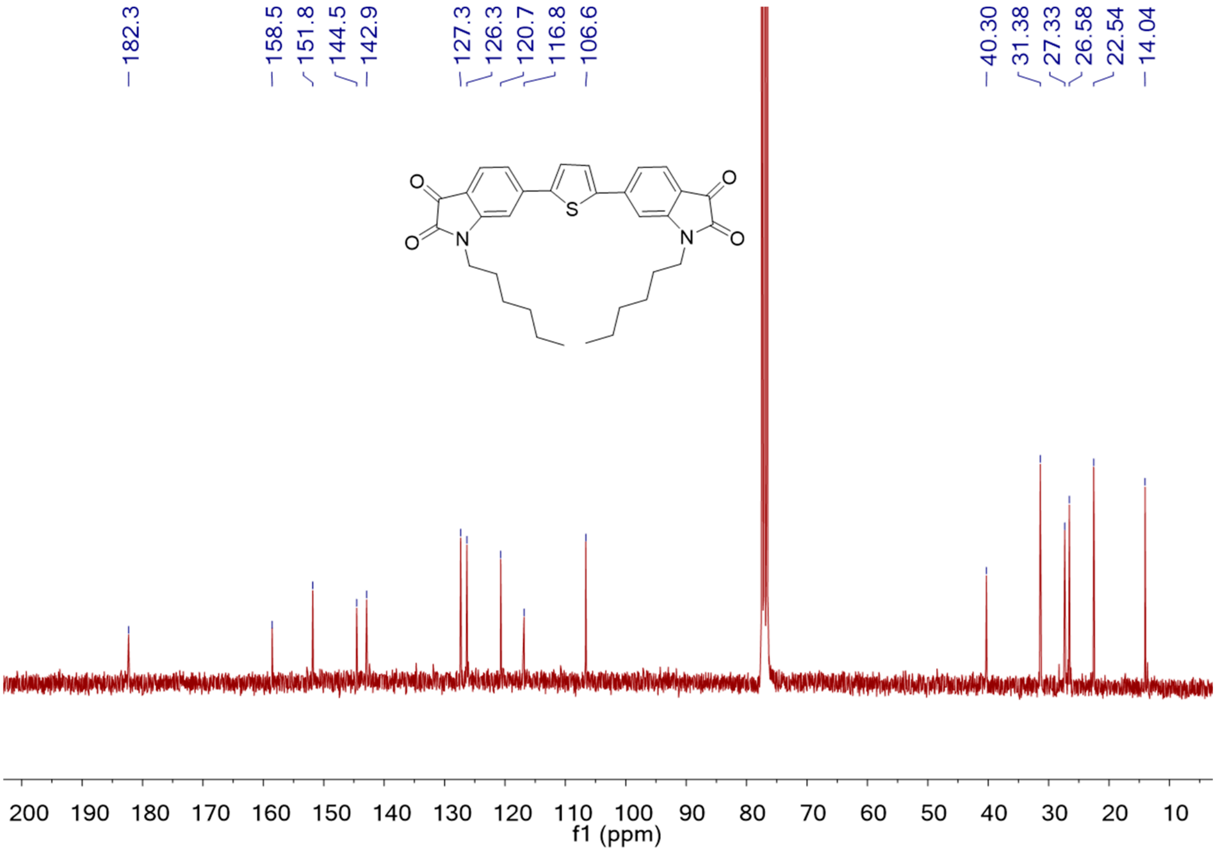

182.3
158.5
151.8
144.5
142.9
127.3
126.3
120.7
116.8
106.6
40.30
31.38
27.33
26.58
22.54
14.04
O
N
S
200 190 180 170 160 150 140 130 120 110 100 90 80 70 60 50 40 30 20 10
f1 (ppm)

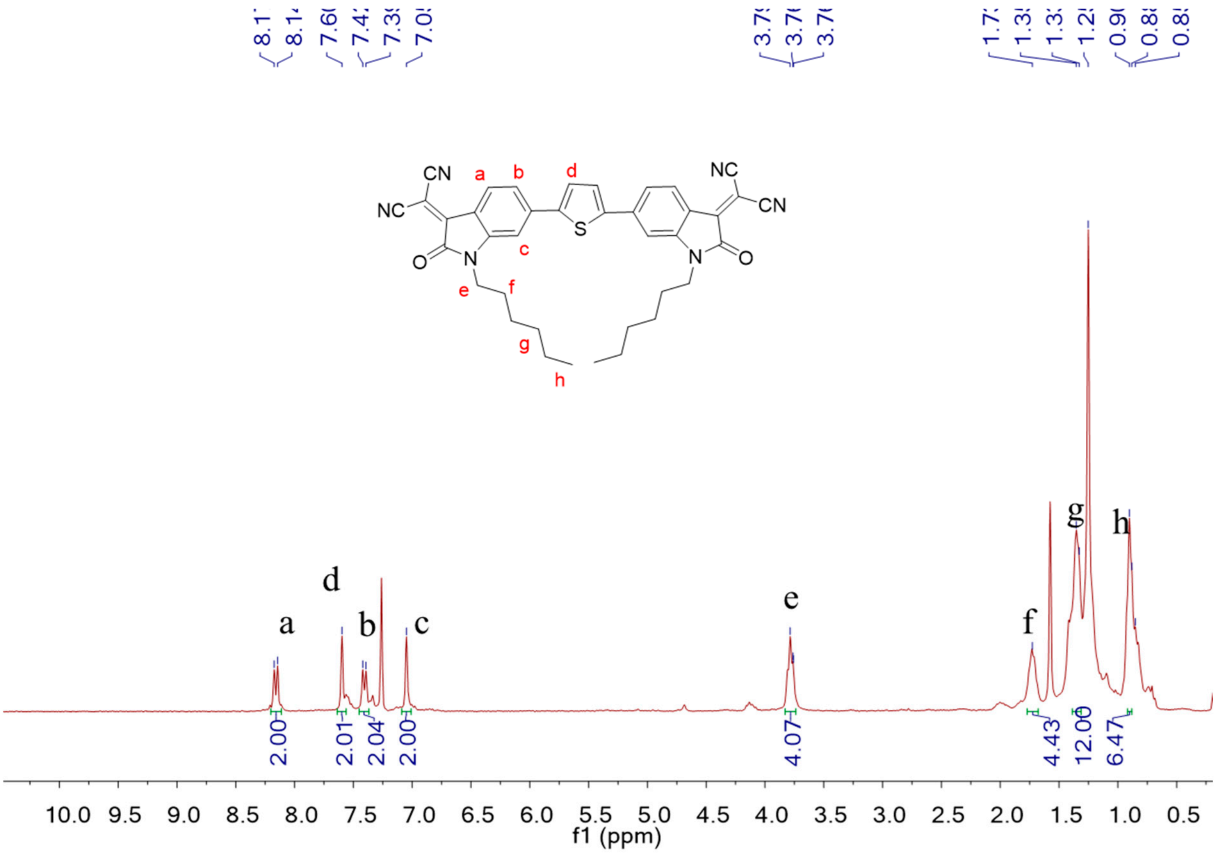
CN
NC
a
b
c
d
S
N
O
e
f
g
h
2.00
2.01
2.04
2.00
4.07
4.43
12.00
6.47
10.0 9.5 9.0 8.5 8.0 7.5 7.0 6.5 6.0 5.5 5.0 4.5 4.0 3.5 3.0 2.5 2.0 1.5 1.0 0.5
f1 (ppm)

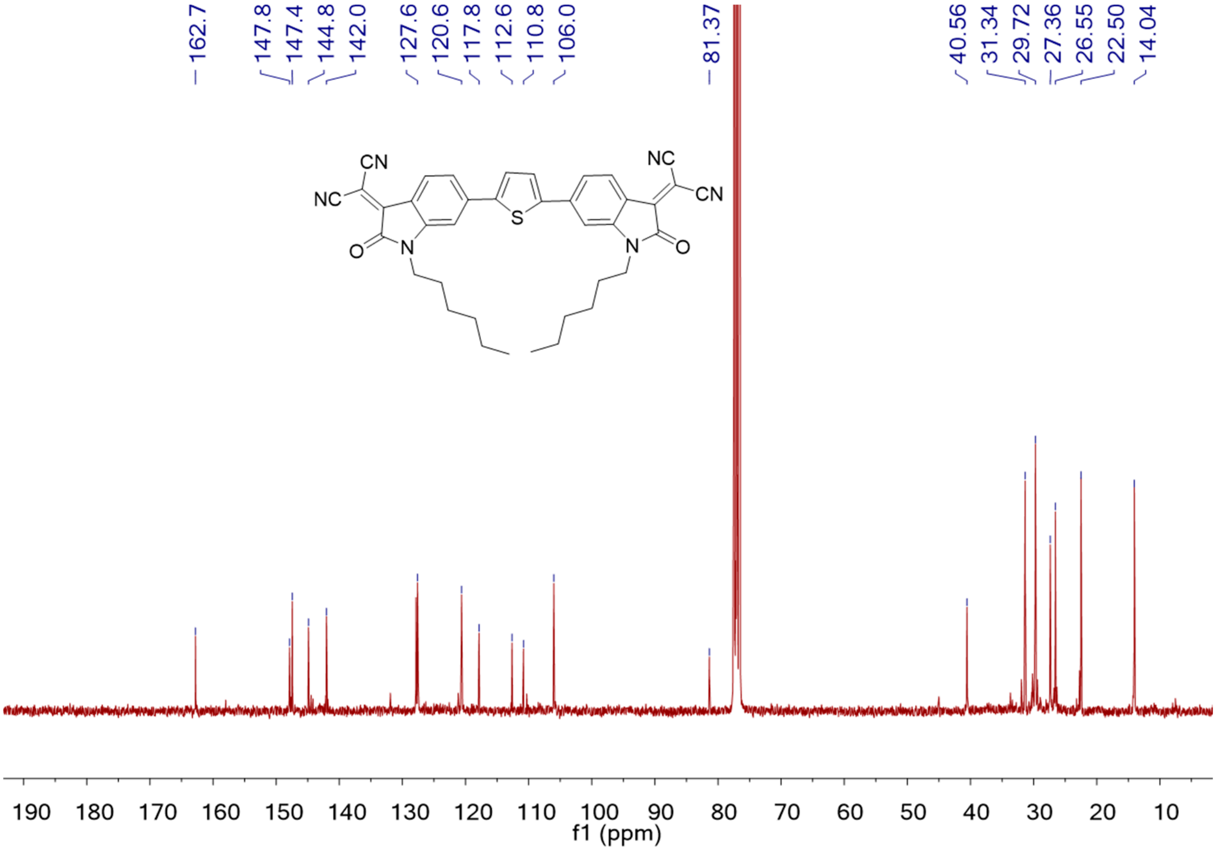
162.7
147.8
147.4
144.8
142.0
127.6
120.6
117.8
112.6
110.8
106.0
81.37
40.56
31.34
29.72
27.36
26.55
22.50
14.04
CN
NC
S
N
O
190 180 170 160 150 140 130 120 110 100 90 80 70 60 50 40 30 20 10
f1 (ppm)

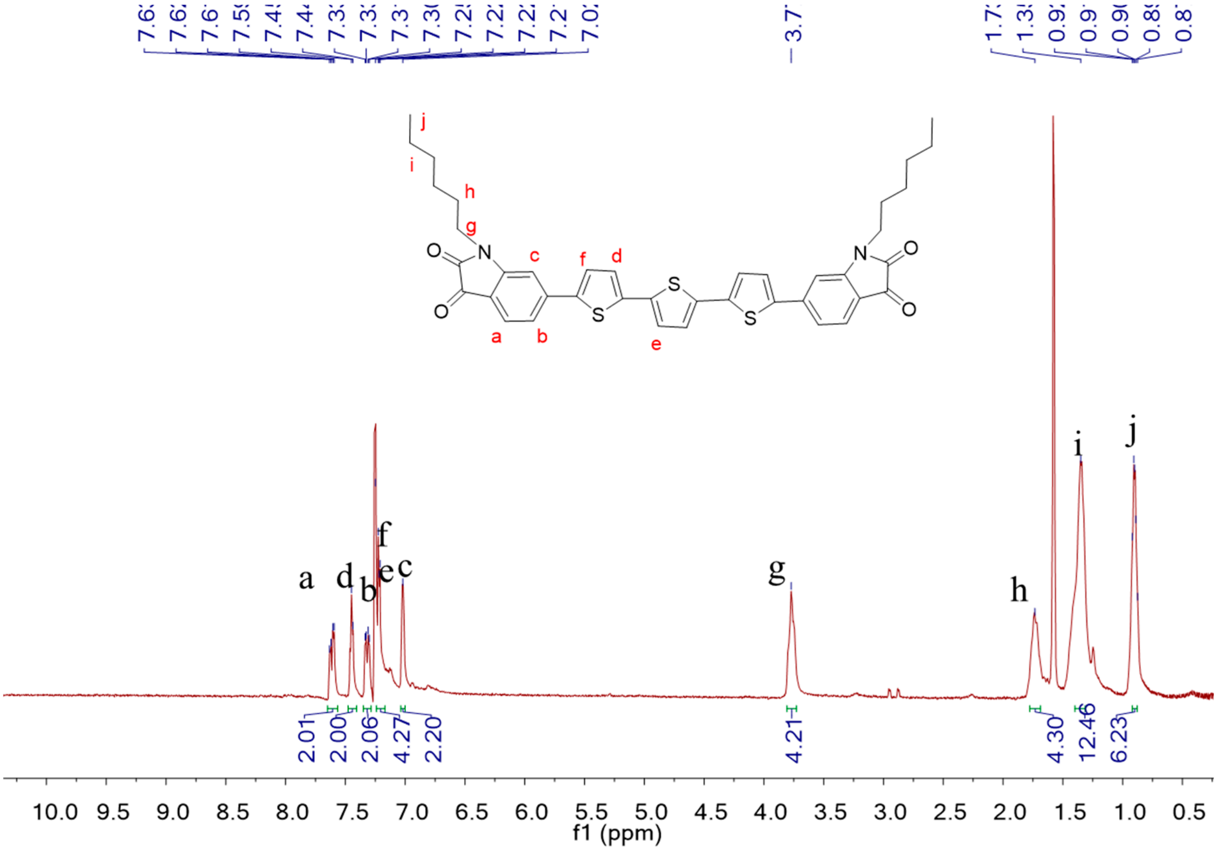

3.7
O
N
O
c
f
d
S
S
S
a
b
e
j
i
h
g
a
d
b
f
e
c
g
h
i
j
2.01
2.00
2.06
4.27
2.20
4.21
4.30
12.46
6.23
10.0 9.5 9.0 8.5 8.0 7.5 7.0 6.5 6.0 5.5 5.0 4.5 4.0 3.5 3.0 2.5 2.0 1.5 1.0 0.5
f1 (ppm)

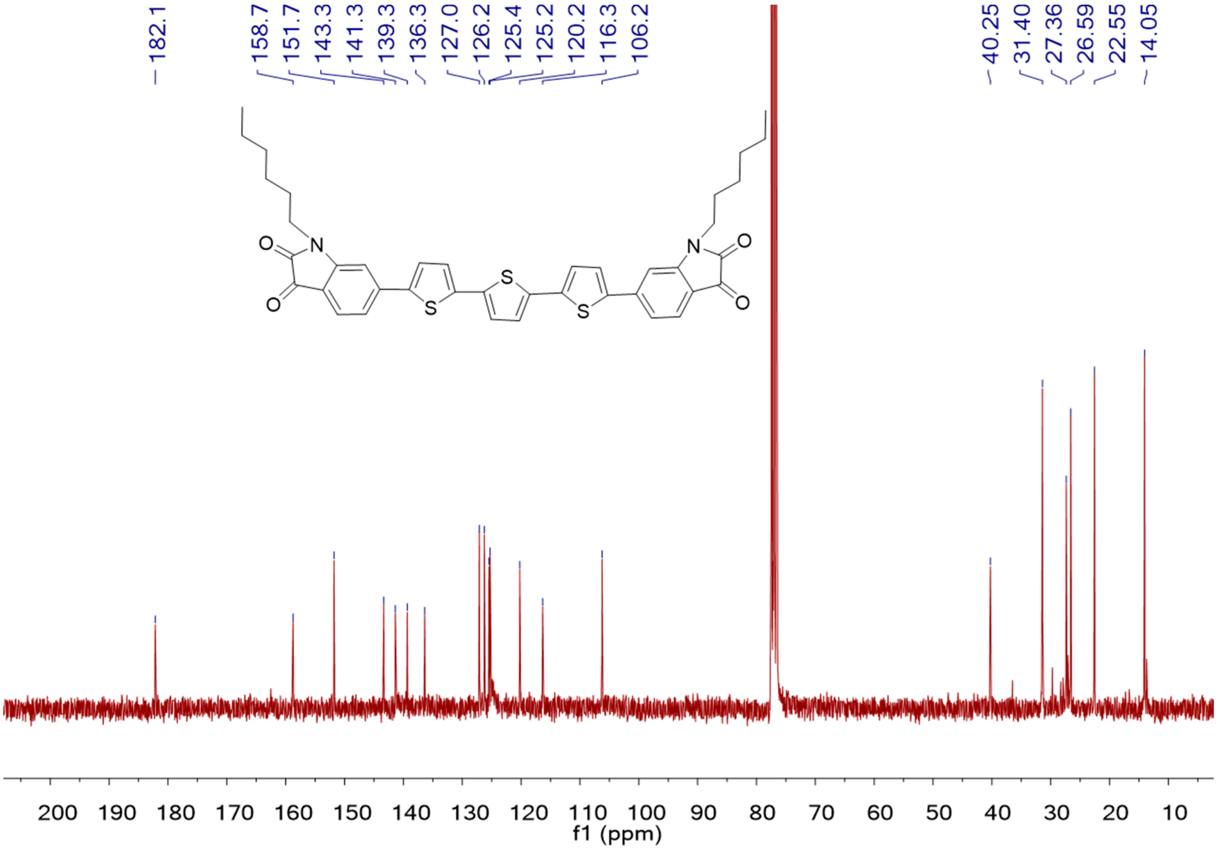

182.1
158.7
151.7
143.3
141.3
139.3
136.3
127.0
126.2
125.4
125.2
120.2
116.3
106.2
40.25
31.40
27.36
26.59
22.55
14.05
O
N
S
200 190 180 170 160 150 140 130 120 110 100 90 80 70 60 50 40 30 20 10
f1 (ppm)

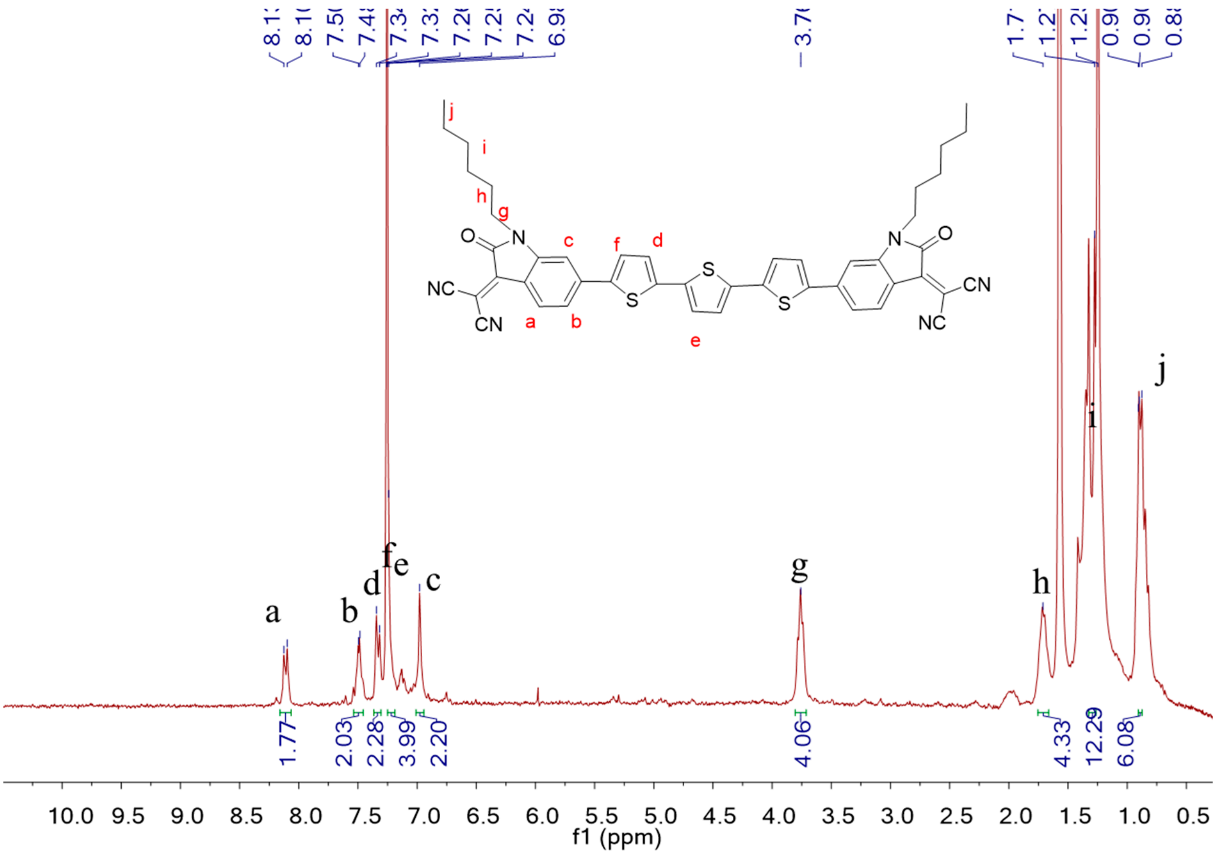
a
b
d
fe
c
g
h
i
j
1.77
2.03
2.28
3.99
2.20
4.06
4.33
12.29
6.08
f1 (ppm)

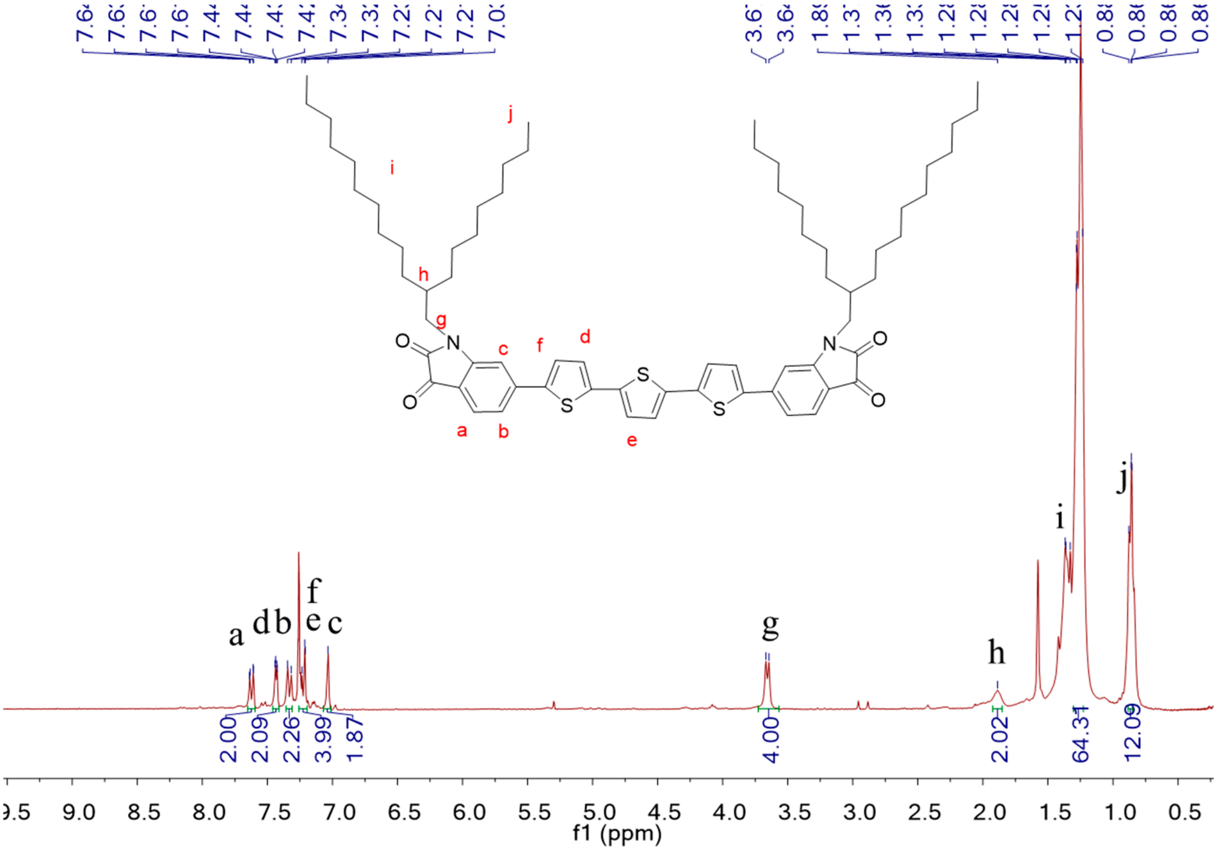
a
d b
f
e
c
g
h
i
j
2.00
2.09
2.26
3.99
1.87
4.00
2.02
64.31
12.09
f1 (ppm)

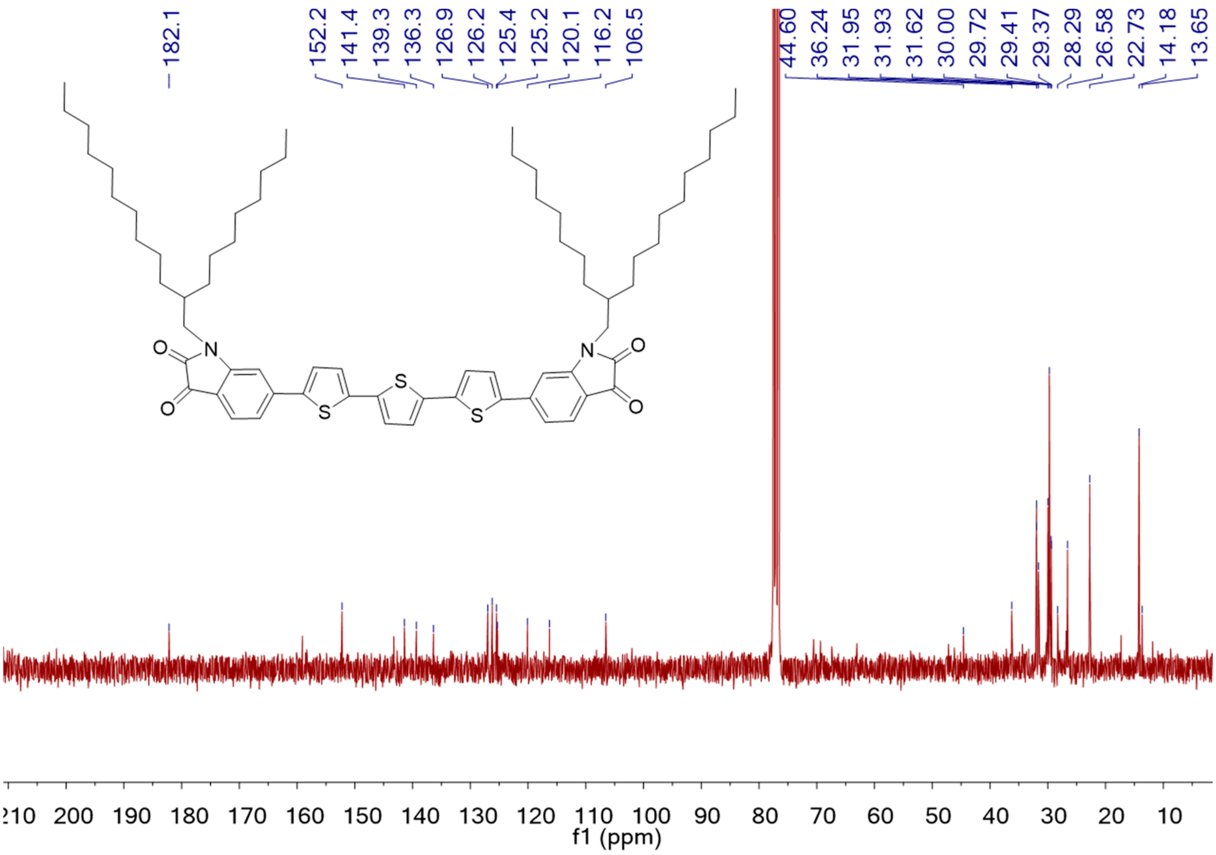
182.1
152.2
141.4
139.3
136.3
126.9
126.2
125.4
125.2
120.1
116.2
106.5
44.60
36.24
31.95
31.93
31.62
30.00
29.72
29.41
29.37
28.29
26.58
22.73
14.18
13.65
O
N
O
S
S
S
N
O
O
210 200 190 180 170 160 150 140 130 120 110 100 90 80 70 60 50 40 30 20 10
f1 (ppm)

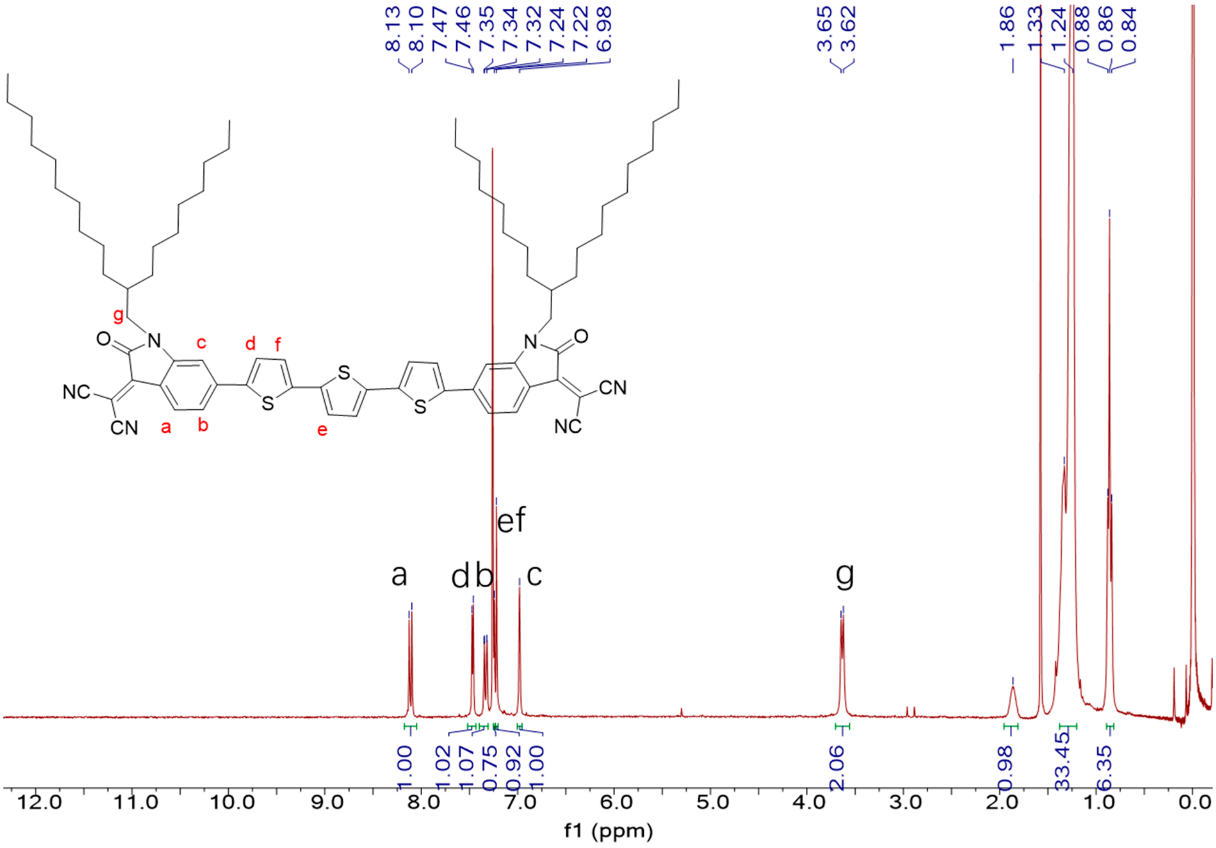
8.13
8.10
7.47
7.46
7.35
7.34
7.32
7.24
7.22
6.98
3.65
3.62
1.86
1.33
1.24
0.88
0.86
0.84
g
O
N
c
d
f
S
S
S
N
O
NC
CN
a
b
e
CN
NC
a
d b
ef
c
g
1.00
1.02
1.07
0.75
0.92
1.00
2.06
0.98
33.45
6.35
12.0 11.0 10.0 9.0 8.0 7.0 6.0 5.0 4.0 3.0 2.0 1.0 0.0
f1 (ppm)

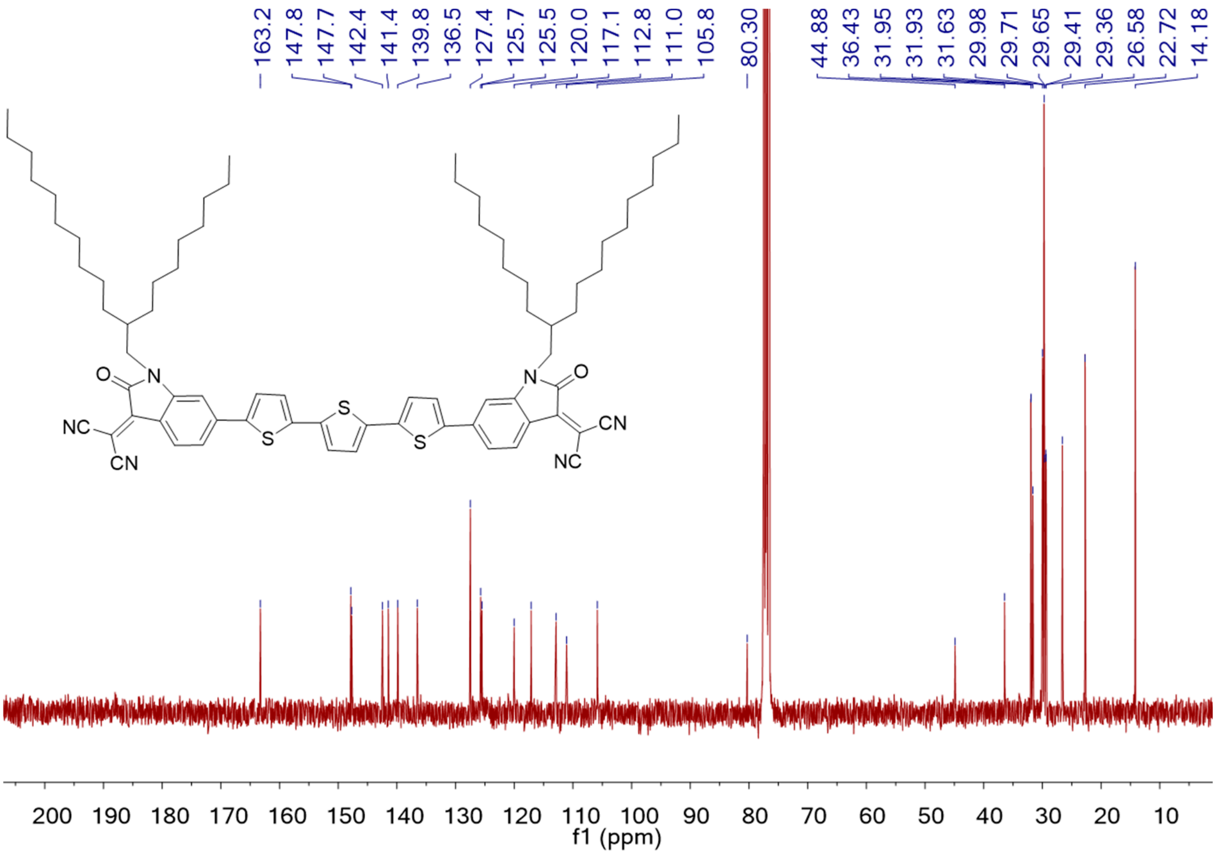
163.2
147.8
147.7
142.4
141.4
139.8
136.5
127.4
125.7
125.5
120.0
117.1
112.8
111.0
105.8
80.30
44.88
36.43
31.95
31.93
31.63
29.98
29.71
29.65
29.41
29.36
26.58
22.72
14.18
NC
CN
O
N
S
200 190 180 170 160 150 140 130 120 110 100 90 80 70 60 50 40 30 20 10
f1 (ppm)

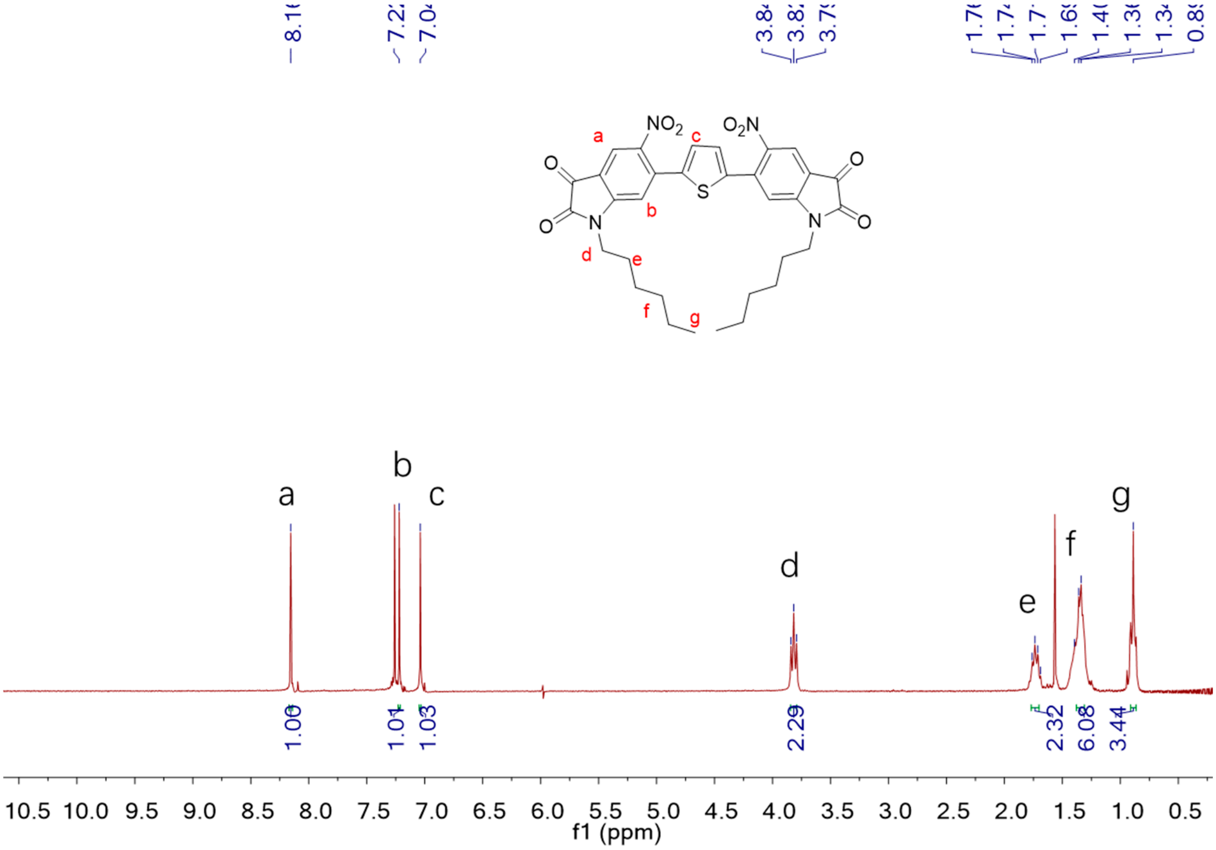
NO2
O2N
a
b
c
d
e
f
g
O
N
S
1.00
1.04
1.03
2.29
2.32
6.08
3.44
10.5 10.0 9.5 9.0 8.5 8.0 7.5 7.0 6.5 6.0 5.5 5.0 4.5 4.0 3.5 3.0 2.5 2.0 1.5 1.0 0.5
f1 (ppm)

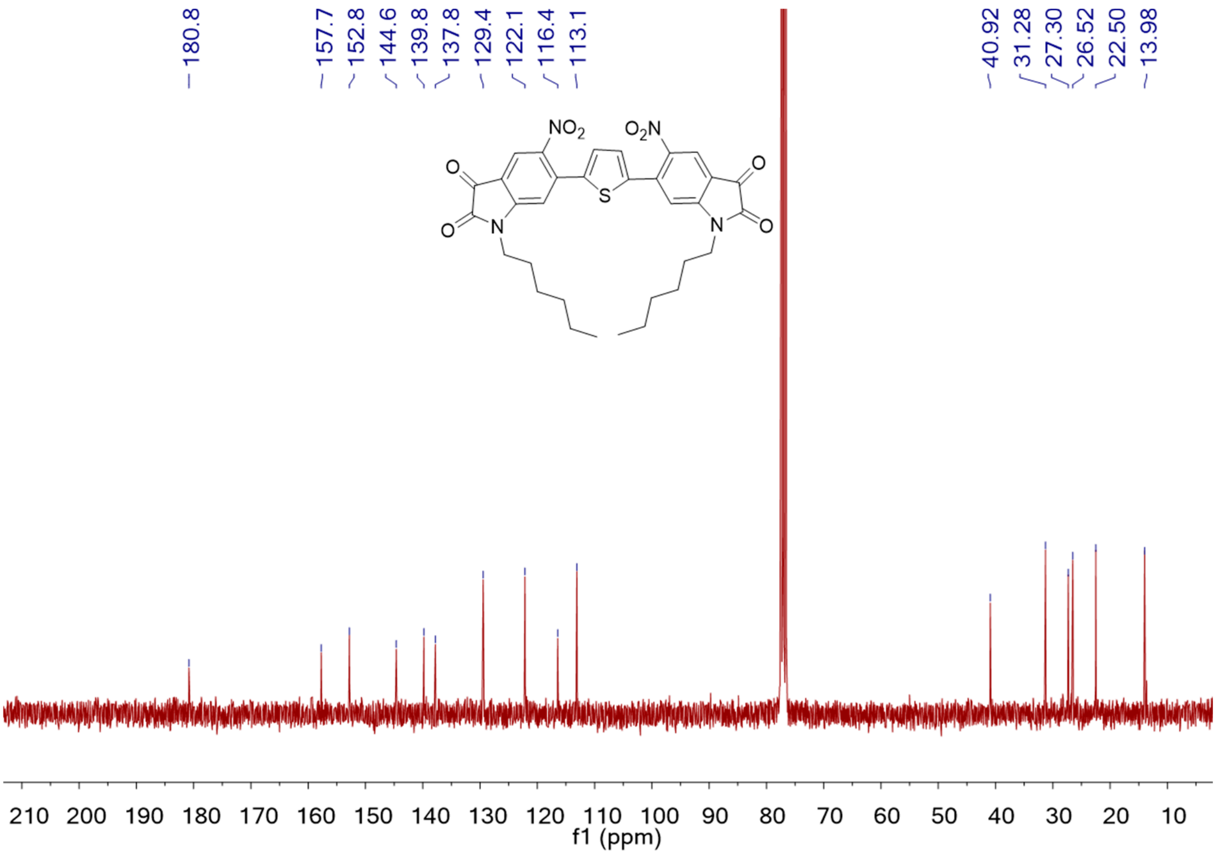
180.8
157.7
152.8
144.6
139.8
137.8
129.4
122.1
116.4
113.1
40.92
31.28
27.30
26.52
22.50
13.98
NO2
O2N
S
N
O
210 200 190 180 170 160 150 140 130 120 110 100 90 80 70 60 50 40 30 20 10
f1 (ppm)

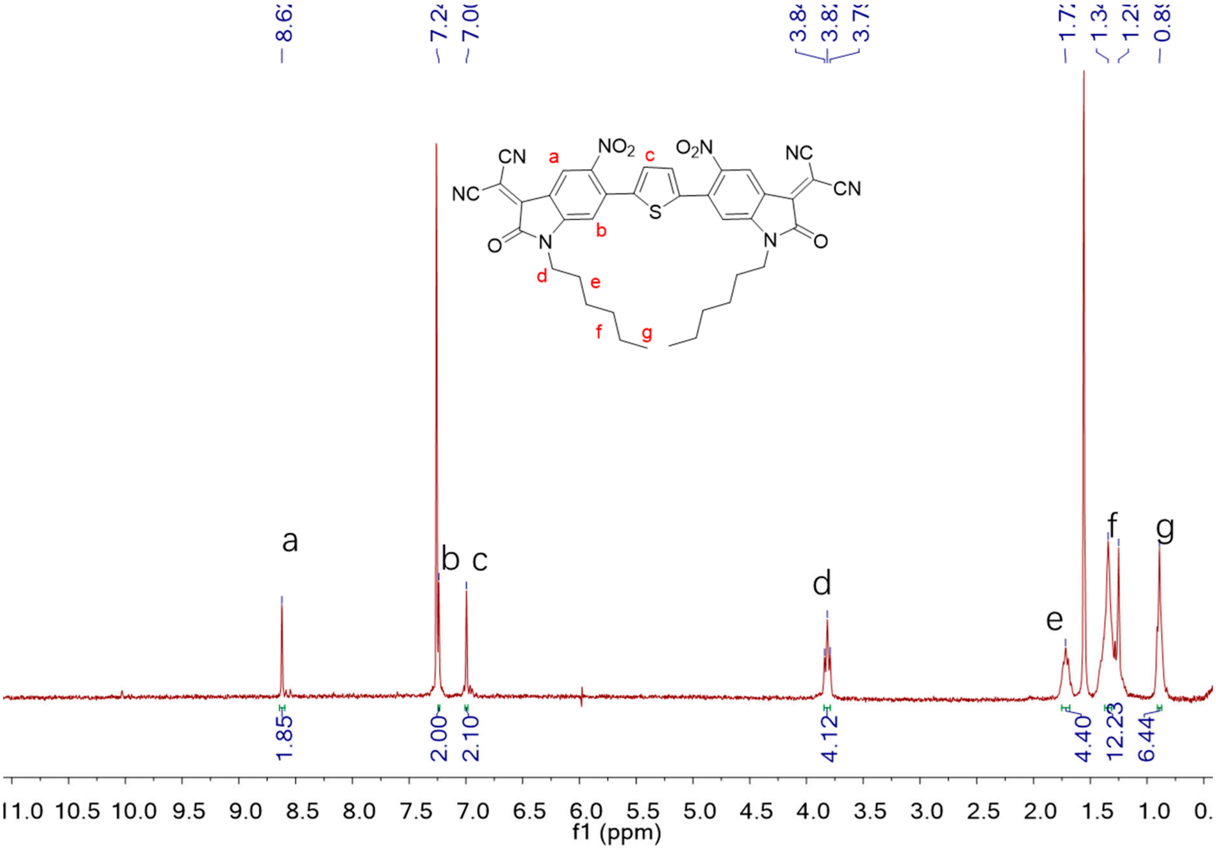
CN
NC
NO2
O2N
S
N
O
a
b
c
d
e
f
g
1.85
2.00
2.10
4.12
4.40
12.23
6.44
10.5 10.0 9.5 9.0 8.5 8.0 7.5 7.0 6.5 6.0 5.5 5.0 4.5 4.0 3.5 3.0 2.5 2.0 1.5 1.0
f1 (ppm)

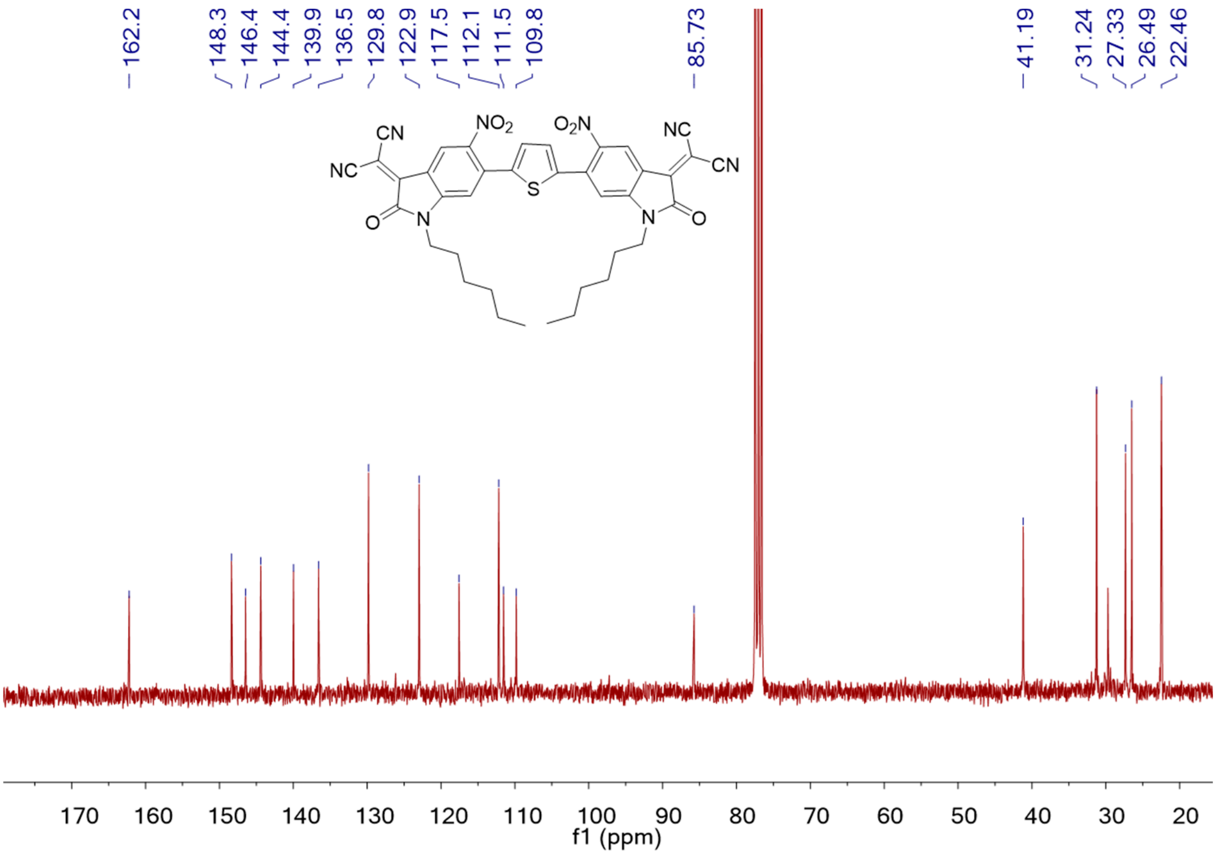
162.2
148.3
146.4
144.4
139.9
136.5
129.8
122.9
117.5
112.1
111.5
109.8
85.73
41.19
31.24
27.33
26.49
22.46
CN
NC
NO2
O2N
NC
CN
S
O
N
N
O
170 160 150 140 130 120 110 100 90 80 70 60 50 40 30 20
f1 (ppm)
